# Formic Acid as Carbon Monoxide Source in the Palladium-Catalyzed N-Heterocyclization of *o*-Nitrostyrenes to Indoles

**DOI:** 10.1021/acs.joc.2c02613

**Published:** 2023-01-19

**Authors:** Manar Ahmed Fouad, Francesco Ferretti, Fabio Ragaini

**Affiliations:** † Dipartimento di Chimica, Università degli Studi di Milano, Via C. Golgi 19, 20133 Milano, Italy; ‡ Chemistry Department, Faculty of Science, Alexandria University, P.O. Box 426, Alexandria 21321, Egypt

## Abstract

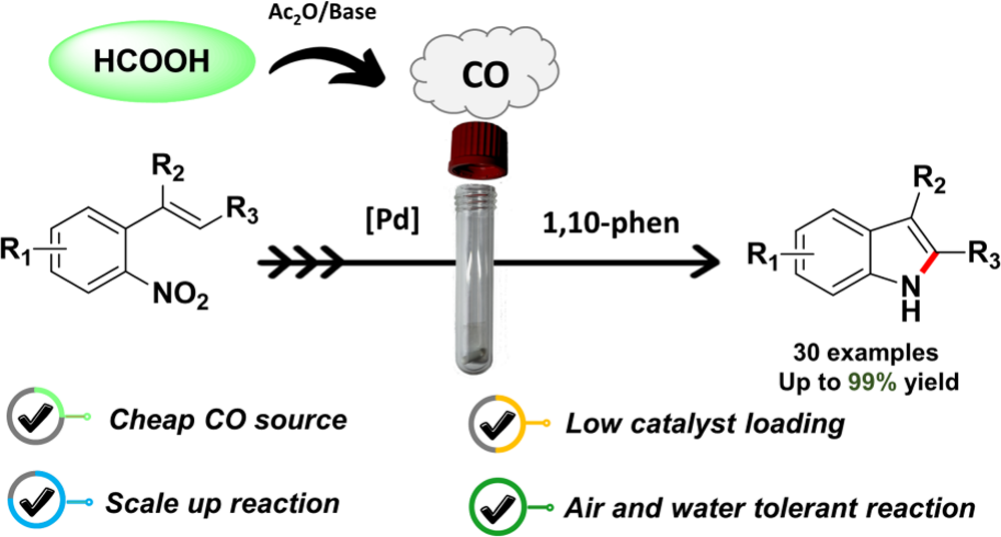

The reductive cyclization reaction of *o*-nitrostyrenes to generate indoles has been investigated for three decades using CO as a cheap reducing agent, but it remains an interesting area of research and improvements. However, using toxic CO gas has several drawbacks. As a result, it is highly preferable to use safe and efficient surrogates for *in situ* generation of CO from nontoxic and affordable sources. Among several CO sources that have been previously explored for the generation of gaseous CO, here we report the use of cheap and readily available formic acid as an effective reductant for the reductive cyclization of *o*-nitrostyrenes. The reaction is air and water tolerant and provides the desired indoles in yields up to 99%, at a low catalyst loading (0.5 mol %) and without generating toxic or difficult to separate byproducts. A cheap glass thick-walled “pressure tube” can be used instead of less available autoclaves, even on a 2 g scale, thus widening the applicability of our protocol.

## Introduction

**Scheme 1:**
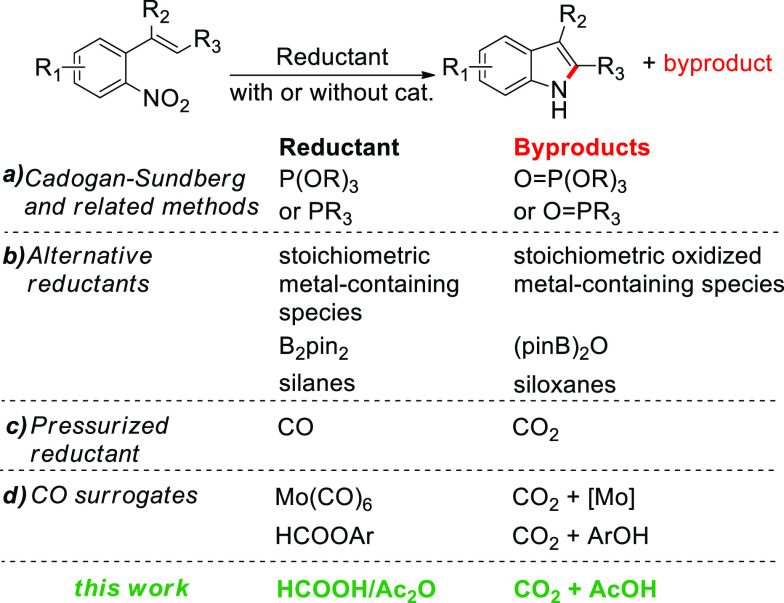
Reductive N-Heterocyclization of *o*-Nitrostyrenes to Indoles: Terminal Reductants and Their Oxidized Counterparts

The reductive N-heterocyclization of *ortho*-nitroaryl-substituted olefins to yield indoles was first investigated by Cadogan^1^ and Sundberg^2^ more than 60 years ago, employing triethyl phosphite both as the reductant and the solvent (Scheme 1a). The protocol has widespread use in synthetic organic laboratories despite its requirement for harsh conditions (reaction temperatures >156 °C) and the fact that it produces a stoichiometric amount of phosphorus wastes and in some cases leads to reduced indole yields due to the formation of *N*-ethoxyindoles as side products.^2,3^ Since then, a large number of different methodologies have been reported to achieve the reductive cyclization of *o*-nitrostyrenes. The use of phosphines as the reductant in uncatalyzed,^4^ metal-catalyzed,^5^ or photochemical^6^ reactions allowed to get rid of the N-ethoxylated side-products. However, the importance of this class of reaction pushed synthetic chemists to develop methodologies employing greener reductants with respect to P(III) compounds and milder reaction conditions. Uncatalyzed electrochemical syntheses,^7^ methods employing stoichiometric metal-containing compounds,^8^ and transition-metal-catalyzed and organocatalyzed reactions employing silanes^9^ or diborane^10^ as terminal reductant have been reported so far (Scheme 1b). Among the various alternatives to P(III) reducing agents, the use of pressurized carbon monoxide has been studied by several different groups in transition-metal^11^ or chalcogen-^12^ catalyzed reactions (Scheme 1c). The first report on this transformation by Cenini and co-workers^11a^ employed harsh conditions (220 °C, 80 bar of CO) and metal carbonyl clusters as the precatalyst. Nicely, subsequent studies on Pd-based catalysts allowed the use of much milder conditions.^11b,11e,11f^ The use of CO as the reductant was also efficiently applied to a number of other reductive cyclization reactions of nitro compounds to yield different heterocyclic scaffolds.^13^ Among those, it is worth mentioning the reductive cyclization of β-nitrostyrenes,^14^ the intramolecular cyclization of 2-alkynyl nitroarenes,^15^ and the intermolecular cyclizations of nitroarenes with alkynes^16^ to indoles that are complementary to the methodology employing *o*-nitrostyrenes as the substrate.

Despite the high efficiency, selectivities, and yields of the reactions and the availability (or easy preparation) of the starting materials, the methodologies employing CO as a reductant for the preparation of heterocycles have been hardly used in synthetic organic laboratories other than those where they were developed. The main obstacle to the diffuse use of these methods is the need for handling pressurized CO and the connected need for high pressure and safety equipment. This problem is not limited to N-heterocyclization reactions but is a common issue for many synthetic protocols for fine chemical production in which CO is employed. A shared problem is a halved problem; thus, researchers developed alternative methods in which molecules (CO surrogates) able to release CO under the reaction conditions are used instead of pressurized CO.^17^ In this context, Mo(CO)_6_,^18^ aryl formates,^19^ and CO_2_/silanes^20^ have been used in the synthesis of indoles and related five-membered ring heterocycles from nitro compounds (Scheme 1d). Drawbacks to the use of surrogates are in some cases the relatively high cost, the toxicity of some compounds, the need for specific multichamber reactors, or the difficult separation from the product. During our previous studies on the use of phenyl formate as CO source for the synthesis of different heterocycles, we found that in a few cases, the coproduced phenol might be annoying to separate by column chromatography from the product. Aiming to circumvent the mentioned problems, we wanted to develop a method based on the use of reagents commonly available in all synthetic laboratories. The most convenient molecules in terms of cost, availability, and atom-economy are formaldehyde^21^ and formic acid.^17c,22^ Herein, we describe a method for the synthesis of indoles by reductive cyclization of *ortho*-nitroaryl-substituted olefins that makes use of HCOOH as the source of the reductant and that can be performed in inexpensive reactors.

## Results and Discussion

For our initial investigations, we took the reductive cyclization reaction of methyl 2-nitrocinnamate as the model reaction. The tests were performed in screw-cap thick-walled glass tubes (pressure tubes) using HCOOH as the CO source in the presence of 1 mol % of Pd(CH_3_CN)_2_Cl_2_ and 5 mol % of phenanthroline as the catalyst system, under conditions previously optimized for the use of phenyl formates as the CO surrogate.^19c^ Despite the fact that HCOOH can decompose under acidic conditions to CO and H_2_O without the need of an activator under relatively mild conditions,^22b,23^ an activator is needed in most cases.^17c^ Acetic anhydride is particularly convenient because it reacts with HCOOH at low temperatures to give a mixed anhydride, which in turn decomposes releasing CO and acetic acid as the only byproduct.^24^ Attracted by the possibility of using a solid source of the HCOO- fragment, some preliminary tests were performed using formate salts (Table 1, entries 1–4) in combination with acetic anhydride. All tests led to low indole yields regardless of the temperature, reaction time and presence of further base.

**Table 1:** Optimization of the Reaction Conditions for the Reductive Cyclization of Methyl 2-Nitrocinnamate (**1a**) to Methyl 2-Indolecarboxylate (**2a**) Using HCOOH (FA) as the CO Source^a^

Entry	FA derivative	FA/Ac_2_O/Et_3_N to **1a** mol ratio	Solvent	Time (h)	Temp (°C)	Conv. (%)^b^	Yield (%)^b^
1^c^	HCOONH_4_	4.4/4.4/–	CH_3_CN	20	100	4	<1
2^c^	HCOONH_4_	4.4/4.4/–	CH_3_CN	6	140	<1	<1
3^c^	HCOONa	4.4/4.4/–	CH_3_CN	6	120	8	3
4	HCOONa	3/3/0.2	CH_3_CN	6	120	2	3
5	HCOOH	4.4/4.4/4.4	CH_3_CN	4	100	86	64
6	HCOOH	4/4/4	CH_3_CN	6	120	100	87
7	HCOOH	4/4/2	CH_3_CN	6	120	100	88
8	HCOOH	4/4/1	CH_3_CN	6	120	100	69
9	HCOOH	3/3/3	CH_3_CN	6	120	100	92
10	HCOOH	2.1/2.1/2.1	CH_3_CN	6	120	90	73
11	HCOOH	3/3/3	CH_3_CN	4	140	100	89
12	HCOOH	3/3/3	CH_3_CN	8	100	84	71
13	HCOOH	3/3/3	CH_3_CN/DMF^d^	8	100	95	79
14	HCOOH	3/3/3	CH_3_CN	10	100	91	77
15	HCOOH	3/3/3	CH_3_CN	8	110	100	91
16	HCOOH	2.5/2.5/2.5	CH_3_CN	8	110	100	91
17^e^	HCOOH	2.5/2.5/2.5	CH_3_CN	8	110	82	76
**18**^e^	**HCOOH**	**2.5/2.5/2.5**	**Acetone**	**8**	**110**	**100**	**94**
19^e^	HCOOH	2.5/2.5/2.5	MEK^f^	8	110	100	89
20^e^	HCOOH	2.5/2.5/2.5	AcOEt	8	110	54	33
21^e^^,^^g^	HCOOH	2.5/2.5/2.5	Acetone	8	110	100	94
**22^h^**	**HCOOH**	**2.5/2.5/2.5**	**Acetone**	**8**	**110**	**100**	**93**
23^h^^,^^i^	HCOOH	2.5/2.5/2.5	Acetone	10	110	100	93
24^h^^,^^j^	HCOOH	2.5/2.5/2.5	Acetone	10	110	100	94
25^h^^,^^l^	HCOOH	2.5/2.5/–	Acetone	10	110	55	13

a Reaction conditions: 0.50 mmol of **1a**, 1 mol % Pd(CH_3_CN)_2_Cl_2_, 5 mol % 1,10-phenanthroline, HCOOH, Ac_2_O, Et_3_N, solvent 10 mL, in a pressure tube.

b Conversion and yields were determined by GC analysis using biphenyl as the internal standard.

c No Et_3_N was added.

d CH_3_CN/DMF ratio 9:1.

e 0.5 mol % of Pd(CH_3_CN)_2_Cl_2_.

f MEK = methyl ethyl ketone.

g Deoxygenated, but undried, solvent was used.

h 0.5 mol % of Pd(acac)_2_ instead of Pd(CH_3_CN)_2_Cl_2_.

i Aqueous formic acid (85 wt %) was used.

j Pressure tube was assembled in the air.

l Aqueous ammonia (25 wt %) was used.

Using a 1:1 ratio of HCOOH and Ac_2_O in a 4.4-fold excess with respect to **1a** under the experimental conditions previously employed with phenyl formate^19c^ led to a fair **2a** yield (Table 1, entry 5). In all successful reactions, the presence of a base was needed to ensure a fast release of CO, which indeed starts even at room temperature (see Experimental Section). Triethylamine was chosen due to its low cost, low toxicity, and easy separation (bp 89 °C). The amount of base could be decreased with respect to those of HCOOH and Ac_2_O, but a less selective reaction was observed when it was lowered under 2 equiv with respect to the substrate (Table 1, entries 6–9). Even if in previous reports Ac_2_O has been used as a catalytic activator, in our case halving its amount led to indole yields lower than 50%. The cyclization reaction is fast and affords high yields between 140 and 110 °C, whereas it becomes less selective when the temperature is further lowered. Nicely, the amount of HCOOH/Ac_2_O/Et_3_N, and thus of released CO, needed for an effective cyclization is only in slight excess with respect to the 2 equiv required by the stoichiometry of the reaction (Table 1, entry 16).

Further optimization of the catalytic system concerned the solvent. Although reductive cyclization of nitrocompounds to heterocycles is known to perform better in DMF or CH_3_CN, both of these solvents are expensive and toxic,^25^ and the former has a high boiling point that makes its evaporation difficult. Looking for an alternative, using a 0.5 mol % Pd loading to better evidence the different performances, we found that acetone ensures a higher yield by both accelerating the reaction rate and increasing the selectivity toward indole, even when not dried before use (Table 1, entries 17–21). According to this unexpected water tolerance, 85 wt % aqueous HCOOH could also be used, obtaining virtually the same yield (entry 23). However, unfortunately, aqueous ammonia could not be used in place of triethylamine (entry 25).

Having an optimized set of reaction conditions to perform the reductive cyclization of the benchmark substrate in our hands, we started to explore the reactivity of different *o*-nitrostyrenes. Disappointingly, when those conditions were employed in the cyclization of methyl 4-bromo-2-nitrocinnamate **1b** precipitation of palladium black, usually occurring only when complete conversion of the nitro compound is approached, took place before full conversion (77% after 10 h). Indeed, deactivation of the catalyst could be visually detected after a few hours from the start of the reaction, indicating a low stability of the catalytic system. The use of a precatalyst containing a chelating anionic ligand such as Pd(acac)_2_ instead of Pd(CH_3_CN)_2_Cl_2_ was sufficient to stabilize the catalyst and avoid early palladium black formation (Table 2, **2b** and Table 1, entry 22). Worthwhile, the system was also stable to dioxygen, thus allowing to set up the reaction in the air. On the contrary, a strict exclusion of dioxygen was necessary when phenyl formate was used as the CO source.^19c^

**Table 2:**
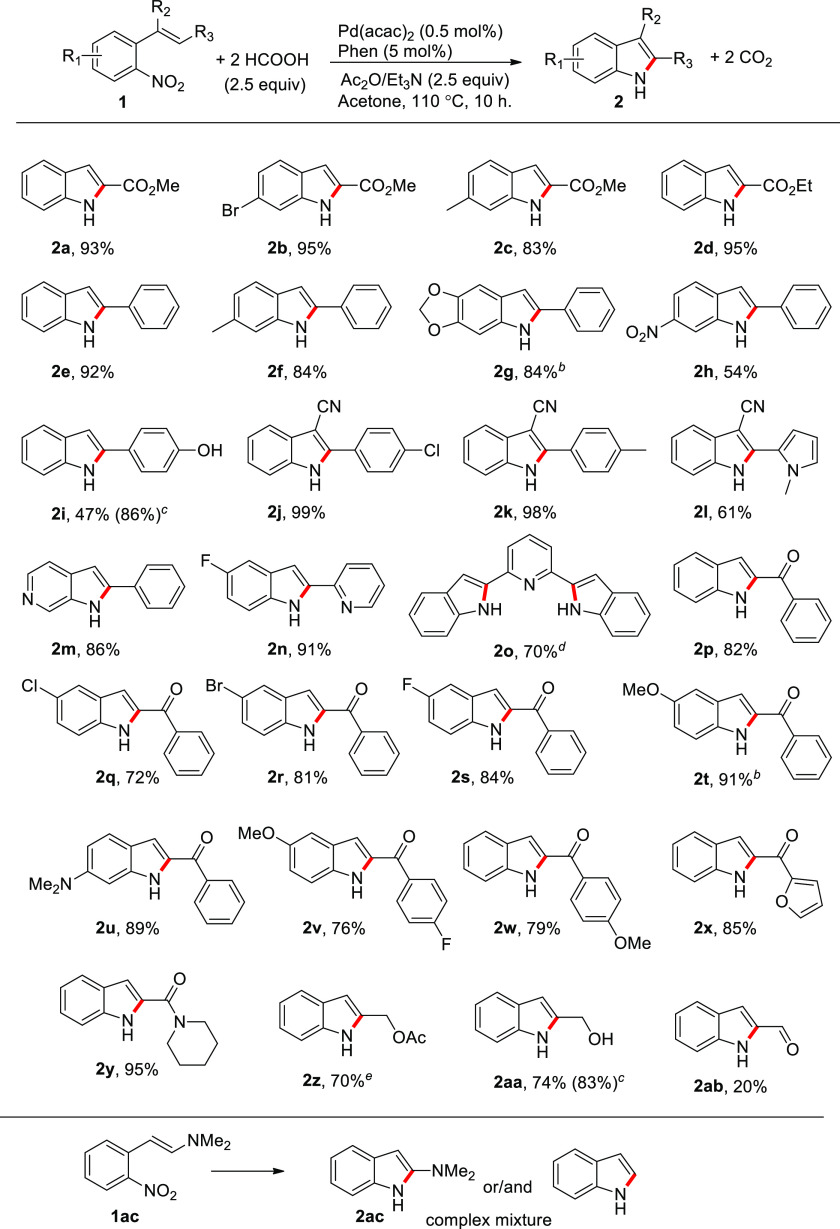
Substrate Scope for the Synthesis of Indoles from *ortho*-Nitroaryl-Substituted Olefins Using HCOOH as the CO Source^a^

a Reaction conditions: 0.50 mmol of **1**, 0.5 mol % Pd(acac)_2_, 5 mol % 1,10-phenanthroline, HCOOH (1.25 mmol), Ac_2_O (1.25 mmol), Et_3_N (1.25 mmol) in 10 mL of acetone, for 10 h. Percentages refer to isolated yields.

b Reaction time 16 h.

c Acetylation of the −OH group was detected. Value in parentheses refers to the overall yield of cyclized products.

d 0.25 mmol of **1o** was used to keep constant the concentration of the nitro groups.

e 1 mol % of Pd(acac)_2_ was used.

Finally, we investigated the substrate scope (Table 2). The reductive cyclization of *o*-nitroaryl-substituted olefins using CO as the reductant was reported to be tolerant of a large number of substituents.^11f,11h^ In our previous studies on the use of phenyl formate as the carbon monoxide source, we demonstrated that the use of surrogates can lead to the use of milder reaction conditions and to yields that in most cases are higher than those obtained using gaseous CO.^19c^ However, the presence of Ac_2_O and formic acid, two quite reactive molecules, made us not take for granted the extension of the high tolerance of the reaction to the present method. However, to our delight, in most cases the cyclization of the substrates to indoles took place in yields comparable or better than those obtained using HCOOPh as the CO source. In most cases 2-nitrocinnamates (**1a-d**), 2-nitrostilbenes (**1e**–**o**), and 2-nitrochalcones (**1p**–**x**) were cyclized to the corresponding indoles in good to excellent yields, regardless of the presence of electron-donating groups (**2c**, **2f**, **2t**–**v**), electron-withdrawing groups (**2b**, **2q**, **2r**), or electron-poor heterocycles (**2m**) on the ring bearing the nitro group. Although electron-donor groups are known to deactivate the nitro group toward reduction, leading to decreased yield in reductive cyclization reactions, we still got very good to excellent yields in all cases. Worth noting, the dioxymethylene-substituted stilbene afforded the corresponding indole (**2g**) in a 12% increased yield with respect to the one previously reported by us.^19c^ The result is interesting since obtaining a good yield for that indole during our previous work was found to be challenging and was possible only by using mild conditions. An opposite trend has been instead obtained for the stilbene bearing two nitro groups on the same aryl ring (**1h**) for which the cyclized product (**2h**) was obtained only in fair yield. In the case of stilbenes, the presence of the electron-poor heterocyclic pyridine ring in position β of the double bond afforded the corresponding indole (**2n**) in high yield, even when a double cyclization was involved (**2o**). The presence of a sensitive pyrrole ring in the substrate (**1l**) was previously found to be problematic when HCOOPh was used as the CO source, leading to a 77% yield only when mild reaction conditions were used.^19c^ A good yield, although slightly lower, was also obtained using HCOOH as the CO source (**2l**). The less reactive furane ring, in the remote position of the nitrochalcone (**1x**), was instead excellently tolerated.

All the cyclization of 2-nitrochalcones (**1p**–**x**) afforded selectively 2-aroyl indoles, which are a class of compounds with anticancer activity.^26^ We did not detect any formation of the corresponding quinoline that was a major side product in several cases in which either CO^11c,27^ or triethyl phosphite^28^ as the reductants were employed for effecting the cyclization.

Remarkably better results than those previously obtained using HCOOPh as the CO surrogate were achieved for the cyclization of 4-(2-nitrostyryl)phenol **1i** bearing an unprotected −OH group, for 2-nitrocinnamyl alcohol (**1aa**) and for 2-nitrocinnamylacetate (**1z**). In all the three cases, using HCOOPh as the CO source, it was not possible to isolate a product due to the formation of a complex mixture of side-products.^19c^ Instead, the use of HCOOH/Ac_2_O mixture allowed us to isolate the cyclization products in >70% yield for the three substrates. However, when a free −OH group was present (**2i**, **2aa**), the obtained product was partially acetylated (39 and 9%, respectively). It is worth pointing out that for the two allylic compounds (**1z** and **1aa**), the only previous successful cyclization to indoles reported in the literature employed 10 mol % of a rhodium complex as the catalyst and the yields were low (26 and 11%, respectively, for **2z** and **2aa**).^11h^

Though the method is quite general and leads to high yields in most cases, a poor yield was obtained in the cyclization of 2-nitrocinnamaldehyde for which HCOOPh was instead effective, allowing the isolation of indole **2ab** in good yield. An increase of the catalyst loading to 1 mol % did not substantially improve the yield (26%). In addition, the cyclization of *trans*-β-dimethylamino-2-nitrostyrene (**1ac**), which was expected to yield either 2-dimethylaminoindole or, more likely, unsubstituted indole,^29^ afforded a complex mixture of products in which the unsubstituted indole was present only in traces.

Finally, a gram-scale reaction was conducted using a *Z*/*E* mixture (2:1 ratio) of 2-nitrostylbene **1e** (2.0 g, 8.8 mmol) under conditions close to the standard ones (see footnote in Table 2). Modifications consisted of the use of half the amount of the required solvent (88 mL instead of 176 mL) and the elongation of the reaction time from 10 h to 12 h. However, since the chromatographic isolation of a multigram amount of product is expensive and time-consuming, the mixture was filtered over Celite to remove the metallic palladium precipitated at full conversion, and the indole was isolated by precipitation with water and filtration. The product was isolated in almost the same yield (90%) as that obtained by the 0.5 mmol-scale reaction (92%). Such an easy workup increases the utility of our protocol.

The mechanism of the reaction was not investigated in the present work, but a mechanism can be proposed on the basis of previous evidence,^27b,30^ and studies are ongoing in our laboratories. Formation of the acetic formic anhydride takes place readily, even at room temperature, and in the presence of the base, it immediately starts to decompose. The formed carbon monoxide reduces the starting Pd(II)/phenanthroline complex to a Pd(0) one, which is responsible for the reduction, operated by one CO molecule, of the nitro group on the *o*-nitrostyrene. The so-formed nitroso group makes an *intra*-molecular electrophilic attack on the double bond, leading to the formation of an *N*-hydroxyindole intermediate, which is in turn reduced by a Pd(0) complex and CO to the indole. Although the general mechanistic scenario is likely very similar to that proposed for the same reaction and catalyst when either compressed CO or phenyl formate are employed as reductant, some small differences must be present. At least in the case of phenyl formate as a CO surrogate, some evidence exists that the initially formed palladium(0) complex is stabilized by coordination of the double bond of the *ortho*-nitroaryl-substituted olefins substrate.^19c^ This feature justifies why a good selectivity in indole **2h** could be obtained in this case despite the fact that the nitro group in the *para* position should be more easily accessible and reactive. If it is further assumed that this olefin-coordinated zerovalent palladium complex is the resting state of the catalytic cycle, even the very high sensitivity to air would be explained. When the protocol described in this paper is applied, the cyclization of **1h** is less selective, though still being favored over the reduction of the nitro group in the *para* position, and the reaction is not sensitive to air, although it is also slower. These observations point to a different resting state of the catalytic system and to lower importance of the olefin-coordinated complex as an intermediate. Whether the latter is anyway formed or not will require a more detailed mechanistic study, which is in progress in our laboratories.

## Conclusions

In summary, we have demonstrated that HCOOH is a convenient CO surrogate for the reductive cyclization of nitro compounds to heterocycles. The new protocol herein presented allows to isolate the desired indoles in most cases with yields comparable to those previously obtained using HCOOPh as the CO source. The use of HCOOPh and HCOOH is found to be complementary for the cyclization of some nitroaryl-substituted olefins, *ortho*-nitroaryl-substituted olefins. The unquestionable advantage of employing HCOOH/Ac_2_O instead of other CO surrogates is the availability in all synthetic organic laboratories, the low cost, and the easy separation of the coproducts formed from the reagents. The reaction well tolerates water and air, thus does not require a strictly oxygen-free environment. The only constraint for the reaction is the need for a (cheap) thick-walled glass pressure tube.

## Experimental Section

### General Information

Unless otherwise stated, all the reactions were carried out under dinitrogen atmosphere using standard Schlenk apparatus. Acetone was degassed and dried over molecular sieves (4 Å) and stored under dinitrogen atmosphere. Formic acid (≥99% purity) and acetic anhydride (≥99% purity) were purchased from Sigma-Aldrich, and formic acid (85% purity) was purchased from Carlo Erba Reagents. All were degassed by freeze–pump–thaw cycles and stored under dinitrogen atmosphere. Triethylamine was distilled from CaH_2_ and kept under dinitrogen atmosphere. 1,10-Phenanthroline (Phen) was purchased as hydrate (TCI Europe NV). It was dissolved in CH_2_Cl_2_, dried over Na_2_SO_4_, followed by filtration under a dinitrogen atmosphere and evaporation of the solvent *in vacuo*. Phen was weighed in the air but stored under dinitrogen to avoid water absorbance. Deuterated solvents were purchased from Sigma-Aldrich: DMSO-*d*_6_ (commercially available in 0.75 mL vials under dinitrogen atmosphere) was used as purchased, while CDCl_3_ was filtered on basic alumina and stored under dinitrogen over 4 Å molecular sieves. All the other reagents were purchased from Merck (Sigma-Aldrich), TCI Europe NV, or Fluorochem and used without further purifications. ^1^H NMR and ^13^C NMR spectra were recorded on a Bruker Avance DRX 400 or Avance NEO 400. Chemical shifts are reported in ppm relative to tetramethylsilane. Thin-layer chromatography (TLC) was performed using precoated silica gel 60 F254 MACHEREY-NAGEL plates. TLC plates were visualized by exposing UV light. Flash column chromatography was performed on MACHEREY-NAGEL flash silica gel 0.04–0.063 mm size. Gas chromatographic analyses were performed using a Shimadzu 2010Pro gas chromatograph equipped with a Supelco SLB-5 ms capillary column (L × I.D. 10 m × 0.10 mm, df 0.10 μm). A standard analysis involves the preparation of a sample solution in ethyl acetate (conc. 0.3 mg/mL calculated with respect to biphenyl used as the internal standard). The Pd complexes used in this work were prepared starting from commercially available PdCl_2_ following procedures reported in the literature.^31^ Compounds **1a**–**m**, **1o**–**p**, **1y-aa**,^19c^ and **1ac**(32) were prepared according to previously reported procedures. Procedures for the preparation of compounds **1n** and **1q**–**x** are reported in the Supporting Information.

### General Procedure for the Preparation of Indoles (2**a**–**ab**)

Stock solutions of the Pd-catalyst and Phen were prepared separately under dinitrogen in acetone to avoid weighing errors. In a typical catalytic reaction, the substrate *o*-nitrostyrene (0.5 mmol) was weighed in the air and placed in a 23 mL thick-walled glass pressure tube (Figure S1) with screw thread (Duran) containing a magnetic stirring bar. (For a more detailed discussion of the different kinds of pressure tubes that can be employed see ref (19c)). The tube was placed inside a Schlenk tube with a wide mouth, evacuated, and filled three times with dinitrogen. The proper volume of stock solutions of the catalysts and Phen was added, and the mixture was stirred (10 min) to enable the formation of the Pd/Phen complex. Subsequently, triethylamine (1.25 mmol) and acetic anhydride (1.25 mmol) were added without stirring, and then the acetone (10 mL total volume) was layered. Finally, formic acid (1.25 mmol) was added, and the pressure tube sealed under dinitrogen. The order of addition of the reagents and solvent layering is crucial to avoid loss of CO that starts to evolve, even at room temperature, as soon as HCOOH, Ac_2_O, and the base are mixed. The pressure tube was then placed and heated while stirring in a custom-made aluminum block preheated to 110 °C. At the end of the reaction, the pressure tube was removed from the aluminum block, allowed to cool to room temperature, and slowly opened under a fume hood. Acetone was evaporated, and the crude was subjected to silica-gel column chromatography using hexane/ethyl acetate as the eluent with the addition of 1 or 2% of Et_3_N to partly deactivate acidic sites of silica gel.

#### Methyl 1H-Indole-2-carboxylate (**2a**)

Obtained as a white solid after purification by flash column chromatography (hexane/ethyl acetate = 9/1 + 1% Et_3_N), (81 mg, 93% yield). ^1^H NMR (400 MHz, CDCl_3_) δ 9.04 (s, 1H), 7.70 (dd, *J* = 8.1, 0.8 Hz, 1H), 7.44 (dd, *J* = 8.3, 0.8 Hz, 1H), 7.33 (ddd, *J* = 8.3, 7.0, 1.1 Hz, 1H), 7.24 (dd, *J* = 2.0, 0.8 Hz, 1H), 7.16 (ddd, *J* = 8.0, 7.0, 1.0 Hz, 1H), 3.96 (s, 3H); ^13^C {^1^H} NMR (100 MHz, CDCl_3_) δ 162.6, 137.0, 127.6, 127.3, 125.6, 122.8, 121.0, 112.0, 109.0, 52.2. Analytical data are consistent with literature values.^19c^

#### Methyl 6-Bromo-1H-indole-2-carboxylate (**2b**)

Obtained as a white solid after purification by flash column chromatography (hexane/ethyl acetate = 9.5/0.5 to 9/1 + 1% Et_3_N), (121 mg, 95% yield). ^1^H NMR (400 MHz, CDCl_3_) δ 9.05 (s, 1H), 7.60 (s, 1H), 7.55 (d, *J* = 8.6 Hz, 1H), 7.26 (dd, *J* = 8.5, 1.7 Hz, 1H, overlapped with CDCl_3_ signal), 7.19 (dd, *J* = 2.1, 0.9 Hz, 1H), 3.96 (s, 3H); ^13^C {^1^H} NMR (100 MHz, CDCl_3_) δ 162.3, 137.6, 127.9, 126.4, 124.6, 124.0, 119.4, 114.9, 109.0, 52.3. Analytical data are consistent with literature values.^19c^

#### Methyl 6-Methyl-1H-indole-2-carboxylate (**2c**)

Obtained as a white solid after purification by flash column chromatography (hexane/ethyl acetate = 9/1 + 1% Et_3_N), (79 mg, 83% yield). ^1^H NMR (400 MHz, CDCl_3_) δ 8.97 (s, 1H), 7.58 (d, *J* = 8.2 Hz, 1H), 7.20 (d, *J* = 3.4 Hz, 2H), 7.00 (d, *J* = 8.2 Hz, 1H), 3.96 (d, *J* = 0.8 Hz, 3H), 2.48 (s, 3H); ^13^C {^1^H} NMR (100 MHz, CDCl_3_) δ 162.7, 137.6, 135.8, 126.7, 125.5, 123.1, 122.3, 111.7, 109.0, 52.0, 22.1. Analytical data are consistent with literature values.^19c^

#### Ethyl 1H-Indole-2-carboxylate (**2d**)

Obtained as a white solid after purification by flash column chromatography (hexane/ethyl acetate = 9/1 + 1% Et_3_N), (89 mg, 95% yield). ^1^H NMR (400 MHz, CDCl_3_) δ 8.93 (s, 1H), 7.70 (dd, *J* = 8.1, 0.9 Hz, 1H), 7.43 (dd, *J* = 8.3, 0.9 Hz, 1H), 7.33 (ddd, *J* = 8.2, 7.1, 1.1 Hz, 1H), 7.24 (dd, *J* = 2.0, 0.9 Hz, 1H), 7.16 (ddd, *J* = 8.0, 7.0, 0.9 Hz, 1H), 4.42 (q, *J* = 7.1 Hz, 2H), 1.42 (t, *J* = 7.1 Hz, 3H); ^13^C {^1^H} NMR (100 MHz, CDCl_3_) δ 162.2, 136.9, 127.8, 127.7, 125.5, 122.8, 120.9, 112.0, 108.8, 61.2, 14.5. Analytical data are consistent with literature values.^19c^

#### 2-Phenyl-1H-indole (**2e**)

Obtained as a shiny colorless solid after purification by flash column chromatography (hexane/ethyl acetate = 9/1 + 1% Et_3_N), (89 mg, 92% yield). ^1^H NMR (400 MHz, CDCl_3_) δ 8.31 (s, 1H), 7.70–7.62 (m, 3H), 7.46 (t, *J* = 7.7 Hz, 2H), 7.41 (dd, *J* = 8.1, 0.7 Hz, 1H), 7.34 (t, *J* = 7.4 Hz, 1H), 7.21 (t, *J* = 7.5 Hz, 1H), 7.14 (t, *J* = 7.5 Hz, 1H), 6.84 (d, *J* = 1.4 Hz, 1H); ^13^C {^1^H} NMR (100 MHz, CDCl_3_) δ 138.0, 137.0, 132.5, 129.4, 129.2, 127.9, 125.3, 122.5, 120.8, 120.4, 111.0, 100.2. Analytical data are consistent with literature values.^19c^

#### 6-Methyl-2-phenyl-1H-indole (**2f**)

Obtained as a white solid after purification by flash column chromatography (hexane/ethyl acetate = 9.5/0.5 + 1% Et_3_N), (87 mg, 84% yield). ^1^H NMR (400 MHz, CDCl_3_) δ 8.17 (s, 1H), 7.65 (d, *J* = 7.7 Hz, 2H), 7.53 (d, *J* = 8.0 Hz, 1H), 7.44 (t, *J* = 7.7 Hz, 2H), 7.32 (t, *J* = 7.4 Hz, 1H), 7.19 (s, 1H), 6.98 (d, *J* = 8.0 Hz, 1H), 6.80 (d, *J* = 1.3 Hz, 1H), 2.49 (s, 3H); ^13^C {^1^H} NMR (100 MHz, CDCl_3_) δ 137.5, 137.4, 132.7, 132.4, 129.1, 127.6, 127.2, 125.1, 122.2, 120.4, 111.0, 100.0, 21.9. Analytical data are consistent with literature values.^19c^

#### 6-Phenyl-5H-[1,3]dioxolo[4,5-*f*]indole (**2g**)

Obtained as a white solid after purification by flash column chromatography (hexane/ethyl acetate = 7/3 + 1% Et_3_N), (101 mg, 84% yield). ^1^H NMR (400 MHz, CDCl_3_) δ 8.25 (s, 1H), 7.64 (d, *J* = 8.0 Hz, 2H), 7.47 (t, *J* = 7.7 Hz, 2H), 7.33 (t, *J* = 7.4 Hz, 1H), 7.06 (s, 1H), 6.92 (s, 1H), 6.76 (d, *J* = 2.0 Hz, 1H), 6.00 (s, 2H); ^13^C {^1^H} NMR (100 MHz, CDCl_3_) δ 145.3, 143.5, 136.8, 132.7, 132.0, 129.2, 127.3, 124.7, 123.4, 100.8, 100.4, 99.3, 92.0. Analytical data are consistent with literature values.^19c^

#### 6-Nitro-2-phenyl-1H-indole (**2h**)

Obtained as an orange solid after purification by flash column chromatography (hexane/ethyl acetate = 7/3 + 1% Et_3_N), (62 mg, 54% yield). ^1^H NMR (400 MHz, DMSO-*d*_6_) δ 12.32 (s, 1H), 8.29 (d, *J* = 1.3 Hz, 1H), 7.98–7.86 (m, 3H), 7.70 (d, *J* = 8.8 Hz, 1H), 7.53 (t, *J* = 7.5 Hz, 2H), 7.43 (t, *J* = 7.3 Hz, 1H), 7.13 (s, 1H); ^13^C {^1^H} NMR (100 MHz, DMSO-*d*_6_) δ 144.2, 141.9, 135.4, 133.7, 130.8, 129.1, 128.9, 125.7, 120.1, 114.8, 107.8, 99.8. Analytical data are consistent with literature values.^19c^

#### 4-(1H-Indol-2-yl)phenol (**2i**)

Obtained as a white solid after purification by flash column chromatography (hexane/ethyl acetate = 8.5/1.5 to 7/3 + 1% Et_3_N), (53 mg, 47% yield). ^1^H NMR (400 MHz, DMSO-*d*_6_) δ 11.29 (s, 1H), 9.59 (s, 1H), 7.67 (d, *J* = 8.6 Hz, 2H), 7.47 (d, *J* = 7.7 Hz, 1H), 7.35 (d, *J* = 7.9 Hz, 1H), 7.03 (t, *J* = 7.3 Hz, 1H), 6.95 (t, *J* = 7.4 Hz, 1H), 6.84 (d, *J* = 8.6 Hz, 2H), 6.67 (d, *J* = 1.2 Hz, 1H); ^13^C {^1^ H} NMR (100 MHz, DMSO-*d*_6_) δ 157.1, 138.3, 136.8, 128.9, 126.4, 123.3, 120.7, 119.5, 119.1, 115.6, 110.9, 96.7. Analytical data are consistent with literature values.^33^

#### 4-(1H-Indol-2-yl)phenyl acetate (**2i′**)

Obtained as a white solid after purification by flash column chromatography (hexane/ethyl acetate = 8.5/1.5 to 7/3 + 1% Et_3_N), (44 mg, 39% yield). ^1^H NMR (400 MHz, DMSO-*d*_6_) δ 11.52 (s, 1H), 7.89 (d, *J* = 8.6 Hz, 2H), 7.53 (d, *J* = 7.8 Hz, 1H), 7.40 (d, *J* = 8.0 Hz, 1H), 7.23 (d, *J* = 8.6 Hz, 2H), 7.10 (t, *J* = 7.3 Hz, 1H), 7.00 (t, *J* = 7.3 Hz, 1H), 6.88 (d, *J* = 1.5 Hz, 1H), 2.29 (s, 3H); ^13^C {^1^H} NMR (100 MHz, DMSO-*d*_6_) δ 169.2, 149.7, 137.1, 136.9, 129.9, 128.6, 126.0, 122.3, 121.6, 120.0, 119.4, 111.3, 98.8, 20.9. Anal. calcd for C_16_H_13_NO_2_: C, 76.48; H, 5.21; N, 5.57; Found: C, 76.59; H, 5.56; N, 5.58.

#### 2-(4-Chlorophenyl)-1H-indole-3-carbonitrile (**2j**)

Obtained as a white solid after purification by flash column chromatography (hexane/ethyl acetate = 7/3 + 1% Et_3_N), (125 mg, 99% yield). ^1^H NMR (400 MHz, DMSO-*d*_6_) δ 12.65 (s, 1H), 8.03–7.96 (m, 2H), 7.75–7.68 (m, 2H), 7.65 (d, *J* = 7.9 Hz, 1H), 7.56 (d, *J* = 8.0 Hz, 1H), 7.33 (t, *J* = 7.6 Hz, 1H), 7.27 (t, *J* = 7.5 Hz, 1H); ^13^C {^1^H} NMR (100 MHz, DMSO-*d*_6_) δ 143.3, 135.6, 134.6, 129.4, 128.6, 128.20, 128.18, 124.1, 122.2, 118.4, 116.7, 112.7, 81.7. Analytical data are consistent with literature values.^19c^

#### 2-(*p*-Tolyl)-1H-indole-3-carbonitrile (**2k**)

Obtained as a white solid after purification by flash column chromatography (hexane/ethyl acetate = 8/2 to 7/3 + 1% Et_3_N), (114 mg, 98% yield). ^1^H NMR (400 MHz, DMSO-*d*_6_) δ 12.51 (s, 1H), 7.89 (d, *J* = 8.2 Hz, 2H), 7.63 (d, *J* = 7.6 Hz, 1H), 7.55 (d, *J* = 8.0 Hz, 1H), 7.43 (d, *J* = 8.2 Hz, 2H), 7.30 (td, *J* = 8, 1.2 Hz, 1H), 7.25 (td, *J* = 7.7, 1.0 Hz, 1H), 2.40 (s, 3H); ^13^C {^1^H} NMR (100 MHz, DMSO-*d*_6_) δ 144.9, 139.8, 135.4, 129.8, 128.3, 126.8, 126.6, 123.7, 121.9, 118.2, 117.1, 112.5, 80.9, 20.9. Analytical data are consistent with literature values.^19c^

#### 2-(1-Methyl-1H-pyrrol-2-yl)-1H-indole-3-carbonitrile (**2l**)

Obtained as a yellow solid after purification by flash column chromatography (hexane/ethyl acetate = 7.5/2.5 to 7/3 + 1% Et_3_N), (67 mg, 61% yield). ^1^H NMR (400 MHz, DMSO-*d*_6_) δ 12.17 (s, 1H), 7.61 (d, *J* = 7.4 Hz, 1H), 7.52 (d, *J* = 7.8 Hz, 1H), 7.30 (td, *J* = 7.5, 1.2 Hz, 1H), 7.25 (td, *J* = 7.5, 0.9 Hz, 1H), 7.11–7.03 (m, 1H), 6.59 (dd, *J* = 3.7, 1.7 Hz, 1H), 6.23 (dd, *J* = 3.5, 2.8 Hz, 1H), 3.80 (s, 3H); ^13^C {^1^H} NMR (100 MHz, DMSO-*d*_6_) δ 138.4, 135.4, 127.6, 126.7, 123.5, 122.1, 121.8, 118.1, 116.8, 112.8, 112.5, 108.3, 82.8, 35.1. Analytical data are consistent with literature values.^19c^

#### 2-Phenyl-1H-pyrrolo[2,3-*c*]pyridine (**2m**)

Obtained as a white solid after purification by flash column chromatography (hexane/ethyl acetate = 2/8 to 1/9 + 2% Et_3_N), (83 mg, 86% yield). ^1^H NMR (400 MHz, DMSO-*d*_6_) δ 12.02 (s, 1H), 8.75 (s, 1H), 8.10 (d, *J* = 5.4 Hz, 1H), 7.93 (d, *J* = 7.6 Hz, 2H), 7.51 (t, *J* = 7.3 Hz, 3H), 7.40 (t, *J* = 7.0 Hz, 1H), 6.97 (s, 1H); ^13^C {^1^H} NMR (100 MHz, DMSO-*d*_6_) δ 141.3, 138.2, 134.22, 134.19, 132.7, 131.3, 129.0, 128.5, 125.8, 114.4, 97.9. Analytical data are consistent with literature values.^19c^

#### 5-Fluoro-2-(2′-pyridinyl)-1H-indole (**2n**)

Obtained as a white solid after purification by flash column chromatography (hexane/ethyl acetate = 8/2 + 1% Et_3_N), (97 mg, 91% yield). ^1^H NMR (400 MHz, DMSO-*d*_6_) δ 11.79 (s, 1H), 8.63 (d, *J* = 4.2 Hz, 1H), 7.96 (d, *J* = 8.0 Hz, 1H), 7.84 (t, *J* = 7.7 Hz, 1H), 7.48 (dd, *J* = 8.5, 4.3 Hz, 1H), 7.39–7.24 (m, 2H), 7.12 (d, *J* = 1.0 Hz, 1H), 7.06–6.90 (m, 1H); ^13^C {^1^H} NMR (100 MHz, DMSO-*d*_6_) δ 157.2 (d, *^*1*^*J**_*C–F*_ = 231.9 Hz), 150.0, 149.2, 138.9, 137.0, 133.9, 128.5 (d, ^3^*J*_*C–F*_ = 10.5 Hz), 122.5, 120.0, 113.0 (d, ^3^*J*_*C–F*_ = 9.8 Hz), 110.6 (d, ^2^*J*_*C–F*_ = 26.3 Hz), 104.9 (d, ^2^*J*_*C–F*_ = 23.1 Hz), 100.6 (d, ^4^*J*_*C–F*_ = 4.9 Hz); ^19^F NMR (376 MHz, DMSO-*d*_6_) δ −124.4 (s). Analytical data are consistent with literature values.^34^

#### 2,6-Di(1H-indol-2-yl)pyridine (**2o**)

Obtained as a white solid after purification by flash column chromatography (hexane/ethyl acetate = 8/2 + 1% Et_3_N), (54 mg, 70% yield). ^1^H NMR (400 MHz, DMSO-*d*_6_) δ 11.71 (s, 2H), 7.92–7.85 (m, 3H), 7.63 (d, *J* = 7.9 Hz, 2H), 7.58 (dd, *J* = 8.0, 0.7 Hz, 2H), 7.28 (dd, *J* = 2.0, 0.7 Hz, 2H), 7.22 (ddd, *J* = 8.1, 7.0, 1.0 Hz, 2H), 7.07 (ddd, *J* = 7.9, 7.0, 0.8 Hz, 2H); ^13^C {^1^H} NMR (100 MHz, DMSO-*d*_6_) δ 149.4, 137.8, 137.0, 136.7, 128.6, 122.7, 120.9, 119.6, 117.8, 111.5, 100.8. Analytical data are consistent with literature values.^19c^

#### (1H-Indol-2-yl)(phenyl)methanone (**2p**)

Obtained as a white solid after purification by flash column chromatography (hexane/ethyl acetate = 9/1 + 1% Et_3_N), (90 mg, 82% yield). ^1^H NMR (400 MHz, CDCl_3_) δ 9.37 (s, 1H), 8.01 (d, *J* = 7.3 Hz, 2H), 7.73 (d, *J* = 8.1 Hz, 1H), 7.63 (t, *J* = 7.4 Hz, 1H), 7.54 (t, *J* = 7.5 Hz, 2H), 7.49 (d, *J* = 8.3 Hz, 1H), 7.39 (t, *J* = 7.5 Hz, 1H), 7.21–7.15 (m, 2H); ^13^C {^1^H} NMR (100 MHz, CDCl_3_) δ 187.3, 138.2, 137.7, 134.5, 132.5, 129.4, 128.6, 127.9, 126.7, 123.4, 121.2, 112.9, 112.3. Analytical data are consistent with literature values.^19c^

#### (5-Chloro-1H-indol-2-yl)(phenyl)methanone (**2q**)

Obtained as a white solid after purification by flash column chromatography (hexane/ethyl acetate = 8.5/1.5 + 1% Et_3_N). (92 mg, 72% yield). ^1^H NMR (400 MHz, CDCl_3_) δ 9.58 (s, 1H), 8.00 (d, *J* = 7.2 Hz, 2H), 7.69 (s, 1H), 7.65 (t, *J* = 7.4 Hz, 1H), 7.55 (t, *J* = 7.5 Hz, 2H), 7.43 (d, *J* = 8.8 Hz, 1H), 7.33 (dd, *J* = 8.8, 1.8 Hz, 1H), 7.10 (d, *J* = 1.0 Hz, 1H); ^13^C {^1^H} NMR (100 MHz, CDCl_3_) δ 187.3, 137.8, 135.9, 135.5, 132.8, 129.4, 128.8, 128.7, 127.1, 126.8, 122.4, 113.5, 111.9. Analytical data are consistent with literature values.^35^

#### (5-Bromo-1H-indol-2-yl)(phenyl)methanone (**2r**)

Obtained as a white solid after purification by flash column chromatography (hexane/ethyl acetate = 8.5/1.5 + 1% Et_3_N), (122 mg, 81% yield). ^1^H NMR (400 MHz, DMSO-*d*_6_) δ 12.16 (s, 1H), 7.96–7.88 (m, 3H), 7.69 (t, *J* = 7.4 Hz, 1H), 7.59 (t, *J* = 7.5 Hz, 2H), 7.47 (d, *J* = 8.8 Hz, 1H), 7.41 (dd, *J* = 8.8, 1.9 Hz, 1H), 7.10 (s, 1H); ^13^C {^1^H} NMR (100 MHz, DMSO-*d*_6_) δ 186.5, 137.7, 136.5, 135.2, 132.5, 128.9, 128.7, 128.6, 128.2, 125.0, 114.8, 112.7, 111.2. Analytical data are consistent with literature values.^36^

#### (5-Fluoro-1H-indol-2-yl)(phenyl)methanone (**2s**)

Obtained as a white solid after purification by flash column chromatography (hexane/ethyl acetate = 9.5/0.5 + 1% Et_3_N), (100 mg, 84% yield). ^1^H NMR (400 MHz, CDCl_3_) δ 9.94 (s, 1H), 8.02 (d, *J* = 7.1 Hz, 2H), 7.65 (t, *J* = 7.4 Hz, 1H), 7.56 (t, *J* = 7.5 Hz, 2H), 7.47 (dd, *J* = 9.0, 4.3 Hz, 1H), 7.35 (dd, *J* = 9.1, 2.3 Hz, 1H), 7.16 (dd, *J* = 9.1, 2.5 Hz, 1H), 7.13–7.12 (m, 1H); ^13^C {^1^H} NMR (100 MHz, CDCl_3_) δ 187.4, 158.4 (d, ^*1*^*J*_*C–F*_ = 237.4 Hz), 138.0, 135.8, 134.5, 132.7, 129.4, 128.7, 127.9 (d, ^3^*J*_*C–F*_ = 10.3 Hz), 115.9 (d, ^2^*J*_*C–F*_ = 27.1 Hz), 113.5 (d, ^3^*J*_*C–F*_ = 9.5 Hz), 112.7 (d, ^3^*J*_*C–F*_ = 5.7 Hz), 107.3 (d, ^2^*J*_*C–F*_ = 23.2 Hz); ^19^F NMR (376 MHz, CDCl_3_) δ −122.33 (td, *J* = 9.1, 4.3 Hz). Analytical data are consistent with literature values.^37^

#### (5-Methoxy-1H-indol-2-yl)(phenyl)methanone (**2t**)

Obtained as a white solid after purification by flash column chromatography (hexane/ethyl acetate = 9/1 + 1% Et_3_N), (114 mg, 91% yield). ^1^H NMR (400 MHz, CDCl_3_) δ 9.84 (s, 1H), 8.02 (d, *J* = 7.2 Hz, 2H), 7.63 (t, *J* = 7.4 Hz, 1H), 7.54 (t, *J* = 7.5 Hz, 2H), 7.42 (d, *J* = 8.8 Hz, 1H), 7.15–7.01 (m, 3H), 3.86 (s, 3H); ^13^C {^1^H} NMR (100 MHz, CDCl_3_) δ 187.3, 155.0, 138.3, 134.9, 133.5, 132.4, 129.4, 128.6, 128.2, 118.6, 113.5, 112.6, 102.9, 55.8. Analytical data are consistent with literature values.^36^

#### (6-(Dimethylamino)-1H-indol-2-yl)(phenyl)methanone (**2u**)

Obtained as an orange solid after purification by flash column chromatography (hexane/ethyl acetate = 8/2 + 1% Et_3_N), (118 mg, 89% yield). ^1^H NMR (400 MHz, DMSO-*d*_6_) δ 11.46 (s, 1H), 7.87 (d, *J* = 7.0 Hz, 2H), 7.63 (t, *J* = 7.3 Hz, 1H), 7.55 (t, *J* = 7.4 Hz, 2H), 7.50 (d, *J* = 9.0 Hz, 1H), 6.98 (d, *J* = 1.3 Hz, 1H), 6.77 (dd, *J* = 9.0, 2.2 Hz, 1H), 6.60 (d, *J* = 1.4 Hz, 1H), 2.95 (s, 6H); ^13^C {^1^H} NMR (100 MHz, DMSO-*d*_6_) δ 184.9, 149.9, 140.6, 138.7, 132.7, 131.6, 128.6, 128.4, 123.3, 119.3, 113.5, 110.7, 92.5, 40.5. Anal. calcd for C_17_H_16_N_2_O: C, 77.25; H, 6.10; N, 10.60; Found: C, 76.88; H, 6.18; N, 10.29.

#### (4-Fluorophenyl)(5-methoxy-1H-indol-2-yl)methanone (**2v**)

Obtained as a yellow solid after purification by flash column chromatography (hexane/ethyl acetate = 8/2 + 1% Et_3_N), (102 mg, 76% yield). ^1^H NMR (400 MHz, DMSO-*d*_6_) δ 11.86 (s, 1H), 8.00 (dd, *J* = 8.1, 5.8 Hz, 2H), 7.40 (t, *J* = 8.7 Hz, 3H), 7.14 (d, *J* = 1.5 Hz, 1H), 7.03 (d, *J* = 0.8 Hz, 1H), 6.99 (dd, *J* = 9.0, 1.8 Hz, 1H), 3.76 (s, 3H); ^13^C {^1^H} NMR (100 MHz, DMSO-*d*_6_) δ 184.8, 165.5 (d, ^1^*J*_*C–F*_ = 250.3 Hz), 154.1, 134.5 (d, ^4^*J*_*C–F*_ = 2.9 Hz), 134.4, 133.6, 131.5 (d, ^3^*J*_*C–F*_ = 9.2 Hz), 127.3, 117.7, 115.6 (d, ^2^*J*_*C–F*_ = 21.8 Hz), 113.7, 111.5, 102.4, 55.2. ^19^F NMR (376 MHz, DMSO-*d*_6_) δ −107.27 to −107.49 (m). Analytical data are consistent with literature values.^38^

#### (1H-Indol-2-yl)(4-methoxyphenyl)methanone (**2w**)

Obtained as a white solid after purification by flash column chromatography (hexane/ethyl acetate = 8/2 + 1% Et_3_N), (99 mg, 79% yield). ^1^H NMR (400 MHz, DMSO-*d*_6_) δ 11.90 (s, 1H), 7.97 (d, *J* = 8.7 Hz, 2H), 7.71 (d, *J* = 8.0 Hz, 1H), 7.51 (d, *J* = 8.2 Hz, 1H), 7.30 (t, *J* = 7.3 Hz, 1H), 7.15–7.05 (m, 4H), 3.87 (s, 3H); ^13^C {^1^H} NMR (100 MHz, CDCl_3_) δ 185.1, 162.7, 137.7, 134.4, 131.3, 130.4, 127.0, 125.4, 122.7, 120.2, 113.9, 112.6, 111.0, 55.5. Analytical data are consistent with literature values.^39^

#### Furan-2-yl(1H-indol-2-yl)methanone (**2x**)

Obtained as a pale-yellow solid after purification by flash column chromatography (hexane/ethyl acetate = 8.5/1.5 + 1% Et_3_N), (90 mg, 85% yield). ^1^H NMR (400 MHz, DMSO-*d*_6_) δ 11.96 (s, 1H), 8.11 (d, *J* = 0.8 Hz, 1H), 7.75 (d, *J* = 8.0 Hz, 1H), 7.65 (d, *J* = 1.3 Hz, 1H), 7.60 (d, *J* = 3.5 Hz, 1H), 7.51 (d, *J* = 8.3 Hz, 1H), 7.31 (t, *J* = 7.3 Hz, 1H), 7.10 (t, *J* = 7.4 Hz, 1H), 6.80 (dd, *J* = 3.5, 1.6 Hz, 1H); ^13^C {^1^H} NMR (100 MHz, DMSO-*d*_6_) δ 172.1, 151.9, 147.8, 137.8, 133.5, 127.2, 125.7, 122.9, 120.4, 118.9, 112.7, 110.3. Analytical data are consistent with literature values.^39^

#### (1H-Indol-2-yl)(piperidin-1-yl)methanone (**2y**)

Obtained as a white solid after purification by flash column chromatography (hexane/ethyl acetate = 8/2 + 1% Et_3_N), (108 mg, 95% yield). ^1^H NMR (400 MHz, CDCl_3_) δ 9.95 (s, 1H), 7.65 (d, *J* = 8.0 Hz, 1H), 7.45 (d, *J* = 8.3 Hz, 1H), 7.26 (td, *J* = 7.2, 1.1 Hz, 1H), 7.12 (td, *J* = 7.2, 0.8 Hz, 1H), 6.77 (d, *J* = 1.4 Hz, 1H), 3.88 (s, 4H), 1.85–1.54 (m, 6H); ^13^C {^1^H} NMR (100 MHz, CDCl_3_) δ 162.6, 135.9, 129.9, 127.6, 124.1, 121.8, 120.4, 112.0, 104.8, 26.3, 24.8. Analytical data are consistent with literature values.^19c^

#### (1H-Indol-2-yl)methyl acetate (**2z**)

Obtained as a white solid after purification by flash column chromatography (hexane/ethyl acetate/CH_2_Cl_2_ = 8/1/1 + 1%Et_3_N), (66 mg, 70% yield). ^1^H NMR (400 MHz, DMSO-*d*_6_) δ 11.18 (s, 1H), 7.50 (d, *J* = 7.8 Hz, 1H), 7.36 (dd, *J* = 8.1, 0.7 Hz, 1H), 7.09 (td, *J* = 8.2, 1.1 Hz, 1H), 6.98 (td, *J* = 7.9, 0.9 Hz, 1H), 6.44 (d, *J* = 1.3 Hz, 1H), 5.18 (s, 2H), 2.06 (s, 3H); ^13^C {^1^H} NMR (100 MHz, DMSO-*d*_6_) δ 170.1, 136.4, 133.4, 127.4, 121.5, 120.1, 119.0, 111.3, 101.9, 59.0, 20.7. Analytical data are consistent with literature values.^40^

#### (1H-Indol-2-yl)methanol (**2aa**)

Obtained as a yellow solid after purification by flash column chromatography (hexane/ethyl acetate = 8/2 to 6/4 + 1%Et_3_N), (56 mg, 74% yield). ^1^H NMR (400 MHz, DMSO-*d*_6_) δ 10.97 (s, 1H), 7.45 (d, *J* = 7.8 Hz, 1H), 7.32 (d, *J* = 8.1 Hz, 1H), 7.03 (td, *J* = 8, 0.8 Hz 1H), 6.94 (td, *J* = 8, 0.8 Hz 1H), 6.26 (d, *J* = 0.9 Hz, 1H), 5.21 (t, *J* = 5.6 Hz, 1H), 4.61 (d, *J* = 5.6 Hz, 2H); ^13^C {^1^H} NMR (100 MHz, DMSO-*d*_6_) δ 140.1, 136.2, 127.9, 120.5, 119.6, 118.6, 111.0, 98.4, 56.9. Analytical data are consistent with literature values.^40^

#### 1H-Indole-2-carbaldehyde (**2ab**)

Obtained as white solid after purification by flash column chromatography (hexane/ethyl acetate = 8/2 + 1%Et_3_N), (15 mg, 20% yield). ^1^H NMR (400 MHz, CDCl_3_) δ 9.86 (s, 1H), 9.24 (s, 1H), 7.75 (d, *J* = 8.1 Hz, 1H), 7.47 (d, *J* = 8.3 Hz, 1H), 7.40 (t, *J* = 7.6 Hz, 1H), 7.28 (s, 1H), 7.18 (t, *J* = 7.5 Hz, 1H); ^13^C {^1^H} NMR (100 MHz, CDCl_3_) δ 182.3, 138.2, 136.1, 127.50, 127.47, 123.6, 121.4, 115.0, 112.6. Analytical data are consistent with literature values.^19c^

### Procedure for Gram-Scale Reaction

A large-scale reaction was carried out in a 250 mL heavy-walled glass pressure bottle to prepare **2e** under the optimal conditions. The reaction was scaled up increasing the substrate amount 17.6-fold with respect to the standard conditions. The pressure bottle was charged with the solid reagents, substrate **1e** (2.00 g, 8.8 mmol), Pd(acac)_2_ (0.5 mol %), and phenanthroline (5 mol %) and then placed in a Schlenk tube with a large mouth. The tube was evacuated and filled three times with dinitrogen. Acetone (30 mL), triethylamine (3.1 mL, 22 mmol), and acetic anhydride (2.1 mL, 22 mmol) were added, and the mixture stirred for 10 min. The stirring was stopped, and the remaining solvent amount (acetone, 68 mL) was layered. At last, formic acid (0.84 mL, 22 mmol) was added, and the bottle sealed with the screw-cap. The total amount of solvent was only 8.8 times increased instead of 17.6 to facilitate the subsequent workup. The pressure bottle was placed in a preheated (110 °C) oil bath. Despite the possibility that the reaction had already reached full conversion of the substrate, the reaction time was extended from 10 to 12 h to ensure completion. Metallic palladium precipitated on the bottle walls at the end of the reaction. At the end of the reaction, the pressure bottle was raised from the oil bath, allowed to cool to room temperature, and slowly opened under a fume hood. *Attention: scale-up of the reaction should be performed carefully considering the maximum CO pressure developed by HCOOH decomposition and scaling-up the reactor volume accordingly*. Subsequently, the solution was filtered on a short pad of Celite in a Pasteur pipet using cannula technique to get rid of any potential colloidal palladium particles. The product was precipitated with water, collected by filtration on a Buchner funnel, dissolved in ethyl acetate (50 mL), and washed with saturated NaHCO_3_ aqueous solution (3 × 30 mL), brine (50 mL), and water (50 mL). The organic layer was then dried over Na_2_SO_4_ and filtered, and the solvent was evaporated under vacuum to yield the final product as a white, analytically and spectroscopically pure crystalline solid (1.54 g, 90% yield), without the need for any chromatographic purification. Phenanthroline and any other byproducts present in small amounts remained in solution by this procedure.

## Data Availability

The data underlying this study are available in the published article and its online supplementary material.

## Supporting Information Available

Picture of the pressure tubes and pressure bottle employed in the catalytic reactions, procedure for the synthesis and characterization of substrates **1n**, **1q**–**x**, ^1^H NMR, ^13^C {^1^H} NMR, and ^19^F NMR spectra of compounds **1n**, **1q**–**x**, **2a**–**ab** (PDF)
FAIR data, including the primary NMR FID files, for compounds **1n**, **1q**–**x**, **2a**–**ab** (ZIP)
