# Supplementary material for: Formic Acid as Carbon Monoxide Source in the Palladium-Catalyzed N-Heterocyclization of o-Nitrostyrenes to Indoles
Source: J Org Chem. 2023 Jan 19;88(8):5108–17. doi: 10.1021/acs.joc.2c02613 (PMC10127278; doi:10.1021/acs.joc.2c02613)

Supporting Information

Formic Acid as Carbon Monoxide Source in the Palladium-Catalyzed *N*-Heterocyclization of *o*-Nitrostyrenes to Indoles

Manar Ahmed Fouad,[a, b] **Francesco Ferretti,**[a, *] **and Fabio Ragaini**[a]

[a] *Dipartimento di Chimica, Università degli Studi di Milano, Via C. Golgi 19, 20133 Milano, Italy;*

[b] *Chemistry Department, Faculty of Science, Alexandria University, P.O. Box 426, Alexandria 21321, Egypt*

Corresponding author e-mail: francesco.ferretti@unimi.it

Table of contents

1- Picture of the pressure tube and pressure bottle……………………………………… S2
2- Preparation of (*E*)-2-(5-fluoro-2-nitrostyryl)pyridine (**1n**)………………...……... S3
3- General procedure for preparation of chalcones (**1q-x**)…...……………………..… S3
4- Characterization data of compounds (**1q-x**)……………………………………….… S4-S5
5- ^{1}H, ^{13}C and ^{19}F NMR spectra for compounds (**1n, 1q-x, 2a-ab**)…………...…...... S6-S49

1- Picture of the pressure tube and pressure bottle

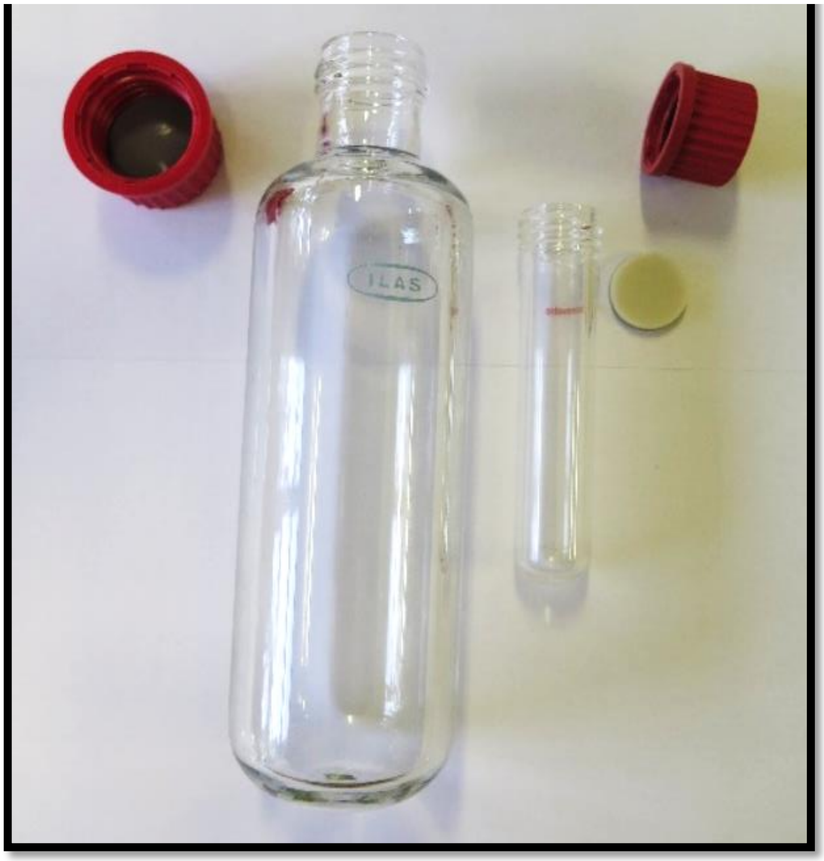

Figure S1. Left: Pressure bottle used for large-scale reaction. Right: Pressure tube used for standard catalytic reactions.

2- Preparation of (*E*)-2-(5-fluoro-2-nitrostyryl)pyridine (1n).

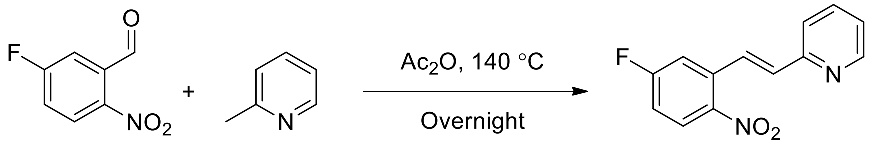

1n, 69%

Compound **1n** was prepared by modification of literature procedure.[1] A Schlenk flask was charged with 5-fluoro-2-nitrobenzaldehyde (1.00 g, 6.0 mmol), 2-picoline (782 mg, 8.4 mmol) and acetic anhydride (2.0 mL) at room temperature. The resulting mixture was refluxed overnight, in an oil bath, under nitrogen atmosphere. After reaction completion, the mixture was distilled at 50 °C under reduced pressure in order to remove acetic anhydride and unreacted 2-picoline. The resulting crude was filtered on silica gel using hexane/ethyl acetate (7/3 to 6/4) as the eluent to give the final product as yellow solid (1.0 g, 69% yield). ^{1}H NMR (400 MHz, $CDCl_3$) δ 8.62 (d, J = 4.1 Hz, 1H), 8.10 – 7.99 (m, 2H), 7.69 (td, J = 7.7, 1.7 Hz, 1H), 7.50 – 7.40 (m, 2H), 7.21 (ddd, J = 7.5, 4.8, 0.8 Hz, 1H), 7.16 – 7.06 (m, 2H); ^{13}C {^{1} H} NMR (100 MHz, $CDCl_3$): δ 164.9 (d, $^1J_{C\text{–}F}$ = 256.3 Hz), 154.5, 150.1, 144.4, 136.8, 136.0 (d, $^3J_{C\text{-}F}$ = 9.3 Hz), 134.4, 127.9 (d, $^3J_{C\text{-}F}$ = 10 Hz), 127.1, 123.3, 122.6, 115.7 (d, $^2J_{C\text{–}F}$ = 23.6 Hz), 115.2 (d, $^2J_{C\text{–}F}$ = 24.2 Hz); ^{19}F NMR (376 MHz, $CDCl_3$) δ -103.78 (s); Anal. Calcd for $C_{13}H_9FN_2O_2$: C, 63.93; H, 3.71; N, 11.47. Found: C, 64.27; H, 3.78; N, 11.39.

3- General procedure for preparation of chalcones (1q-x).[2]

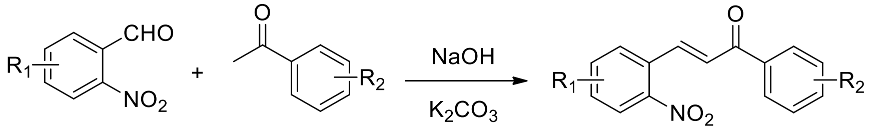

1q-x

In a 10 mL dry round bottomed flask, 2-nitrobenzaldehyde derivative (2.5 mmol) was stirred vigorously with potassium carbonate (0.20 mmol) and sodium hydroxide (0.20 mmol). Acetophenone derivative (2.5 mmol) was added dropwise at room temperature. After 1 hour, water (15 mL) and ethyl acetate (15 mL) were added. The organic layer was separated and the aqueous phase extracted with ethyl acetate (3 × 15 mL). The combined organic layer was washed with water (3 × 50 mL) and brine (50 mL), dried over Na_2SO_4, filtered and the organic solvent removed under reduced pressure to afford the crude product. The crude was recrystallized from ethanol or purified by column chromatography to give the final product.

(E)-3-(5-chloro-2-nitrophenyl)-1-phenylprop-2-en-1-one ***(1q)***. Obtained as a pale-yellow solid after crystallization from ethanol, (398 mg, 55% yield). ^{1}H NMR (400 MHz, $CDCl_3$) δ 8.11 (d, *J* = 15.7 Hz, 1H), 8.06 (d, *J* = 8.8 Hz, 1H), 8.04 – 8.00 (m, 2H), 7.70 (d, *J* = 2.2 Hz, 1H), 7.62 (t, *J* = 7.4 Hz, 1H), 7.57 – 7.49 (m, 3H), 7.33 ppm (d, *J* = 15.6 Hz, 1H); ^{13}C {^{1} H} NMR (100 MHz, $CDCl_3$) δ 190.0, 146.8, 140.3, 139.1, 137.3, 133.5, 133.5, 130.3, 129.3, 129.0, 128.9, 128.3, 126.7; Anal. Calcd for $C_{15}H_{10}ClNO_3$: C, 62.62; H, 3.50; N, 4.87. Found: C, 62.46; H, 3.68; N, 4.72.

(E)-3-(5-bromo-2-nitrophenyl)-1-phenylprop-2-en-1-one ***(1r)***. Obtained as a greenish solid after crystallization from ethanol from ethanol, (413 mg, 50% yield). ^{1}H NMR (400 MHz, $CDCl_3$) δ 8.09 (d, *J* = 15.6 Hz, 1H), 8.05 – 7.99 (m, 2H), 7.96 (d, *J* = 8.7 Hz, 1H), 7.86 (d, *J* = 2.0 Hz, 1H), 7.68 (dd, *J* = 8.7, 2.0 Hz, 1H), 7.61 (t, *J* = 7.4 Hz, 1H), 7.52 (t, *J* = 7.5 Hz, 2H), 7.32 (d, *J* = 15.6 Hz, 1H); ^{13}C {^{1} H} NMR (100 MHz, $CDCl_3$) δ 190.0, 147.2, 138.9, 137.3, 133.5, 133.4, 133.3, 132.3, 128.93, 128.91, 128.6, 128.2, 126.6; Anal. Calcd for $C_{15}H_{10}BrNO_3$: C, 54.24; H, 3.03; N, 4.22. Found: C,54.23; H,3.18; N,4.29.

(E)-3-(5-fluoro-2-nitrophenyl)-1-phenylprop-2-en-1-one ***(1s)***. Obtained as a pale-yellow solid after crystallization from ethanol, (305 mg, 45% yield). ^{1}H NMR (400 MHz, $CDCl_3$) δ 8.20 – 8.09 (m, 2H), 8.05 – 7.98 (m, 2H), 7.64 – 7.59 (m, 1H), 7.52 (t, *J* = 7.5 Hz, 2H), 7.41 (dd, *J* = 8.8, 2.7 Hz, 1H), 7.31 (d, *J* = 15.7 Hz, 1H), 7.26 – 7.19 (m, 1H); ^{13}C {^{1} H} NMR (100 MHz, $CDCl_3$) δ 190.2, 165.0 (d, $^1J_{C–F}$ = 258.0 Hz), 144.7, 139.4, 137.3, 134.9 (d, $^3J_{C-F}$= 9.1 Hz), 133.5, 128.9, 128.3, 128.2 (d, $^3J_{C-F}$= 9.9 Hz), 117.4, 117.3 (d, $^2J_{C–F}$ = 23.4 Hz), 116.2 (d, $^2J_{C–F}$ = 24.3 Hz), 116.1; ^{19}F NMR (376 MHz, $CDCl_3$) δ -102.93 (s); Anal. Calcd for $C_{15}H_{10}FNO_3$: C, 66.42; H, 3.72; N, 5.16. Found: C,66.36; H,3.87; N,5.36.

(E)-3-(5-methoxy-2-nitrophenyl)-1-phenylprop-2-en-1-one ***(1t)***. Obtained as a light-ochre solid after crystallization from ethanol, (413 mg, 50% yield). ^{1}H NMR (400 MHz, $CDCl_3$) δ 8.18 (d, *J* = 15.7 Hz, 1H), 8.13 (d, *J* = 9.1 Hz, 1H), 8.04 – 7.97 (m, 2H), 7.58 (t, *J* = 7.4 Hz, 1H), 7.49 (t, *J* = 7.5 Hz, 2H), 7.21 (d, *J* = 15.6 Hz, 1H), 7.08 (d, *J* = 2.7 Hz, 1H), 6.98 (dd, *J* = 9.1, 2.7 Hz, 1H), 3.93 (s, 3H); ^{13}C {^{1} H} NMR (100 MHz, $CDCl_3$) δ 190.9, 163.6, 141.6, 141.3, 137.5, 134.6, 133.2, 128.9, 128.8, 127.9, 127.4, 114.8, 114.5, 56.2; Anal. Calcd for $C_{16}H_{13}NO_4$: C, 67.84; H, 4.63; N, 4.94. Found: C,67.72; H,4.77; N,4.89.

(E)-3-(4-(dimethylamino)-2-nitrophenyl)-1-phenylprop-2-en-1-one ***(1u)***. Obtained as a red solid after crystallization from ethanol, (482 mg, 65% yield). ^{1}H NMR (400 MHz, $CDCl_3$) δ 8.03 (d, *J* = 15.6 Hz, 1H), 7.98 (d, *J* = 7.8 Hz, 2H), 7.66 (d, *J* = 8.9 Hz, 1H), 7.57 (t, *J* = 7.2 Hz, 1H), 7.49 (t, *J* = 7.5 Hz, 2H), 7.25 (d, *J* = 15.6 Hz, 1H, overlapped with $CDCl_3$ signal), 7.14 (d, *J* = 2.6 Hz, 1H), 6.86 (dd, *J* = 8.9, 2.6 Hz, 1H), 3.08 ppm (s, 6H); ^{13}C {^{1} H} NMR (100 MHz, $CDCl_3$) δ 191.0, 151.5, 150.9, 140.1, 138.3, 132.7,

129.6, 128.7, 128.7, 122.8, 116.6, 115.6, 106.9, 40.3; Anal. Calcd for $C_{17}H_{16}N_2O_3$: C, 68.91; H, 5.44; N, 9.45. Found: C,68.58; H,5.40; N,9.68.

*(E)-1-(4-fluorophenyl)-3-(5-methoxy-2-nitrophenyl)prop-2-en-1-one (**1v**).* Obtained as a off-white solid after purification by crystallization from ethanol, (300 mg, 40% yield). ^{1}H NMR (400 MHz, $CDCl_3$) δ 8.20 (d, *J* = 6.8 Hz, 1H), 8.17 (s, 1H), 8.10 – 8.02 (m, 2H), 7.23 – 7.11 (m, 3H), 7.08 (d, *J* = 2.7 Hz, 1H), 7.01 (dd, *J* = 9.1, 2.8 Hz, 1H), 3.95 (s, 3H); ^{13}C {^{1} H} NMR (100 MHz, $CDCl_3$) δ 189.6, 165.9 (d, $^1J_{C–F}$ = 255.3 Hz), 163.7, 141.9, 134.6, 133.9 (d, $^4J_{C–F}$ = 3 Hz), 131.7 (d, $^3J_{C-F}$ = 9.3 Hz), 128.0, 127.3, 116.0 (d, $^2J_{C–F}$ = 21.9 Hz), 114.8 (d, $^2J_{C–F}$ = 26.5 Hz), 56.3; ^{19}F NMR (376 MHz, $CDCl_3$) δ -104.89 (dq, *J* = 8.4, 5.5 Hz); Anal. Calcd for $C_{16}H_{12}FNO_4$: C, 63.79; H, 4.01; N, 4.65. Found: C,64.03; H,4.26; N,4.84.

*(E)-1-(4-methoxyphenyl)-3-(2-nitrophenyl)prop-2-en-1-one (**1w**).* Obtained as a yellow solid after crystallization from ethanol, (415 mg, 50% yield). ^{1}H NMR (400 MHz, $CDCl_3$) δ 8.10 (d, *J* = 15.6 Hz, 1H), 8.04 (t, *J* = 8.6 Hz, 3H), 7.74 (d, *J* = 7.7 Hz, 1H), 7.67 (t, *J* = 7.5 Hz, 1H), 7.55 (td, *J* = 8.0, 1.3 Hz, 1H), 7.32 (d, *J* = 15.6 Hz, 1H), 6.99 (d, *J* = 8.9 Hz, 2H), 3.89 (s, 3H); ^{13}C {^{1} H} NMR (100 MHz, $CDCl_3$): δ 188.8, 163.9, 148.7, 139.3, 133.6, 131.7, 131.3, 130.5, 130.3, 129.4, 127.5, 125.1, 114.1, 55.7; Anal. Calcd for $C_{16}H_{13}NO_4$: C, 67.84; H, 4.63; N, 4.94. Found: C,67.74; H,4.71; N,4.90.

*(E)-1-(furan-2-yl)-3-(2-nitrophenyl)prop-2-en-1-one (**1x**).* Obtained as a light-green solid after purification by flash column chromatography (hexane/ethyl acetate = 8/2 to 7/3), (261 mg, 43% yield). ^{1}H NMR (400 MHz, $CDCl_3$) δ 8.24 (d, *J* = 15.7 Hz, 1H), 8.05 (dd, *J* = 8.2, 1.1 Hz, 1H), 7.75 (dd, *J* = 7.8, 1.1 Hz, 1H), 7.70 – 7.63 (m, 2H), 7.61 – 7.52 (m, 1H), 7.37 (dd, *J* = 3.6, 0.5 Hz, 1H), 7.29 (d, *J* = 15.7 Hz, 1H), 6.61 (dd, *J* = 3.6, 1.7 Hz, 1H); ^{13}C {^{1} H} NMR (100 MHz, $CDCl_3$) δ 177.5, 153.3, 148.8, 147.1, 139.3, 133.6, 131.3, 130.5, 129.4, 126.5, 125.1, 118.5, 112.9; Anal. Calcd for $C_{13}H_9NO_4$: C, 64.20; H, 3.73; N, 5.76. Found: C,64.31; H,3.75; N,5.81.

References

1. D. H. Leung, J. W. Ziller, Z. Guan, *J. Am. Chem. Soc.* 2008, 130, 24, 7538–7539.

2. G. Zhang, K. Yang, S. Wang, Q. Feng, Q. Song, *Org. Lett.* 2021, 23, 2, 595–600.

^{1}H NMR (400 MHz, $CDCl_3$) of 1n

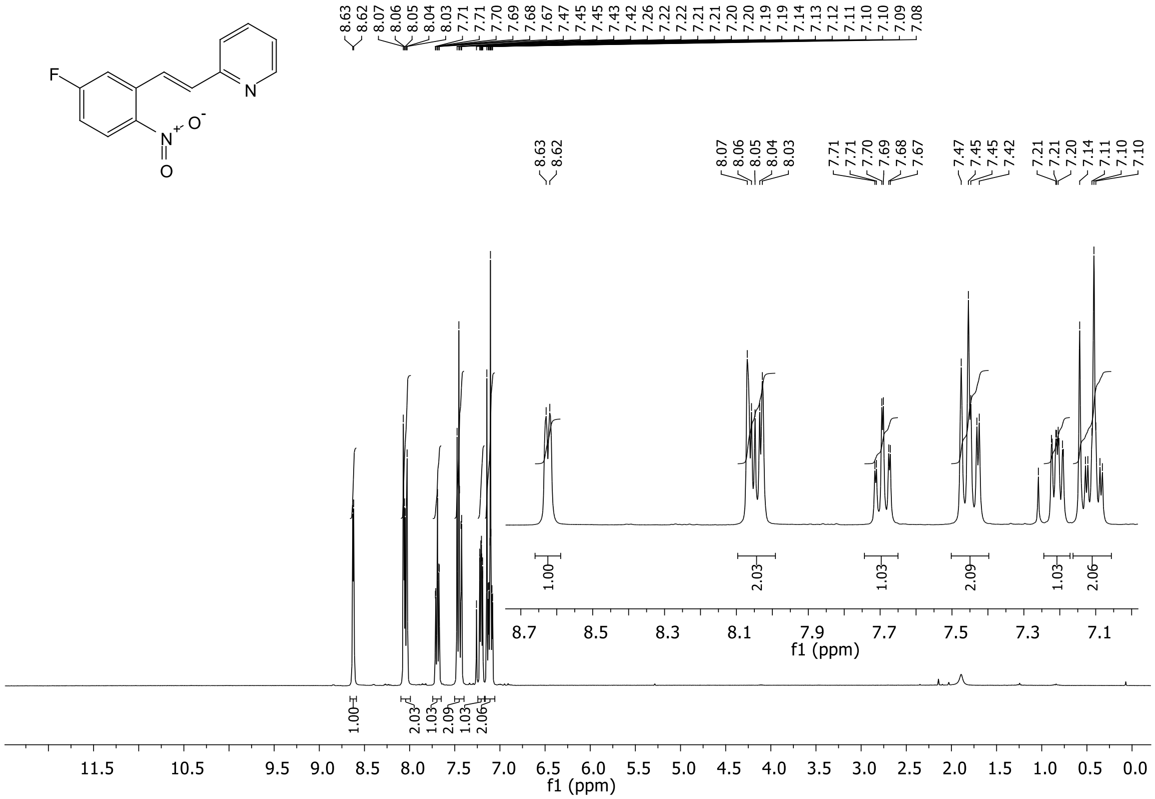

^{13}C {^{1} H} NMR (100 MHz, $CDCl_3$) of 1n

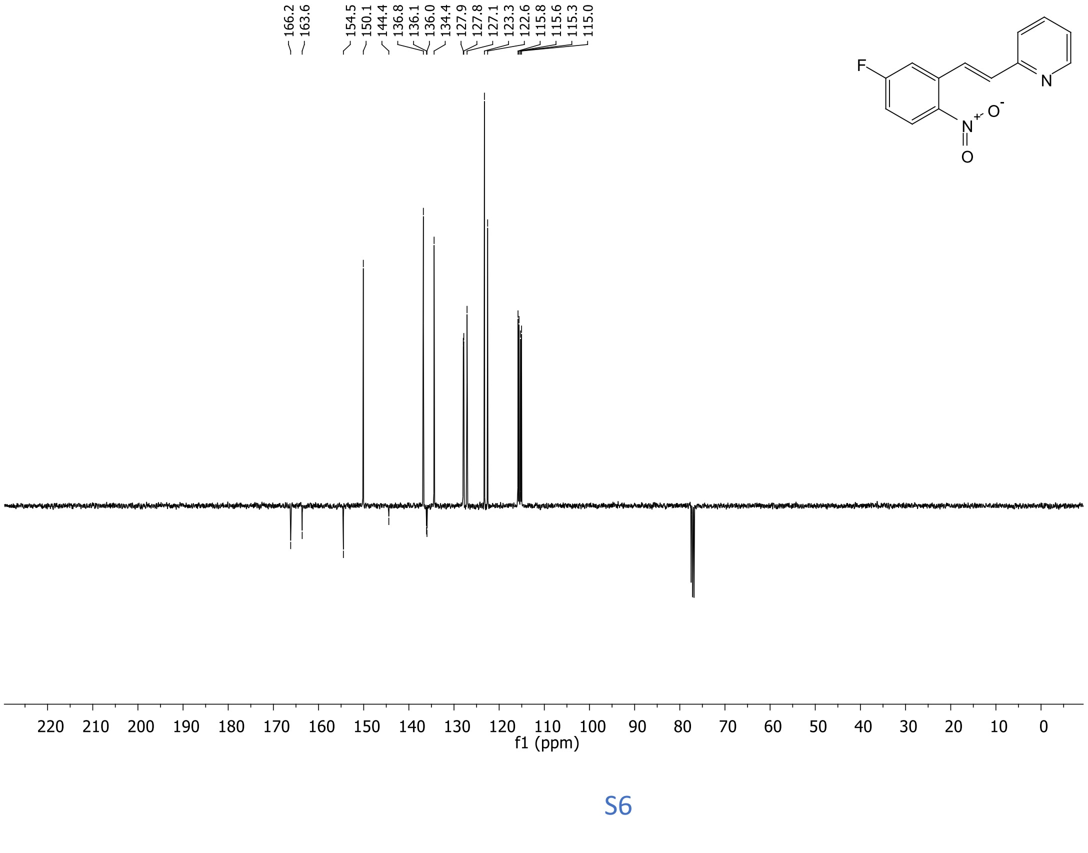

^{19}F NMR (376 MHz, $CDCl_3$) of 1n

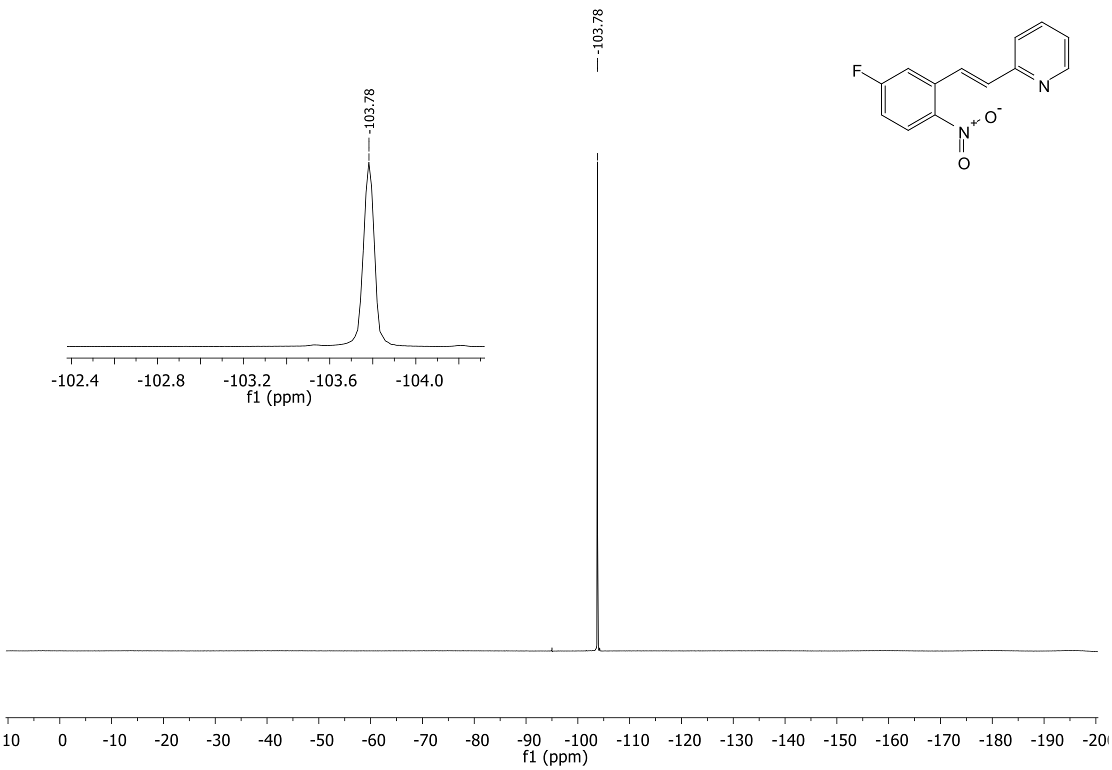

^{1}H NMR (400 MHz, $CDCl_3$) of 1q

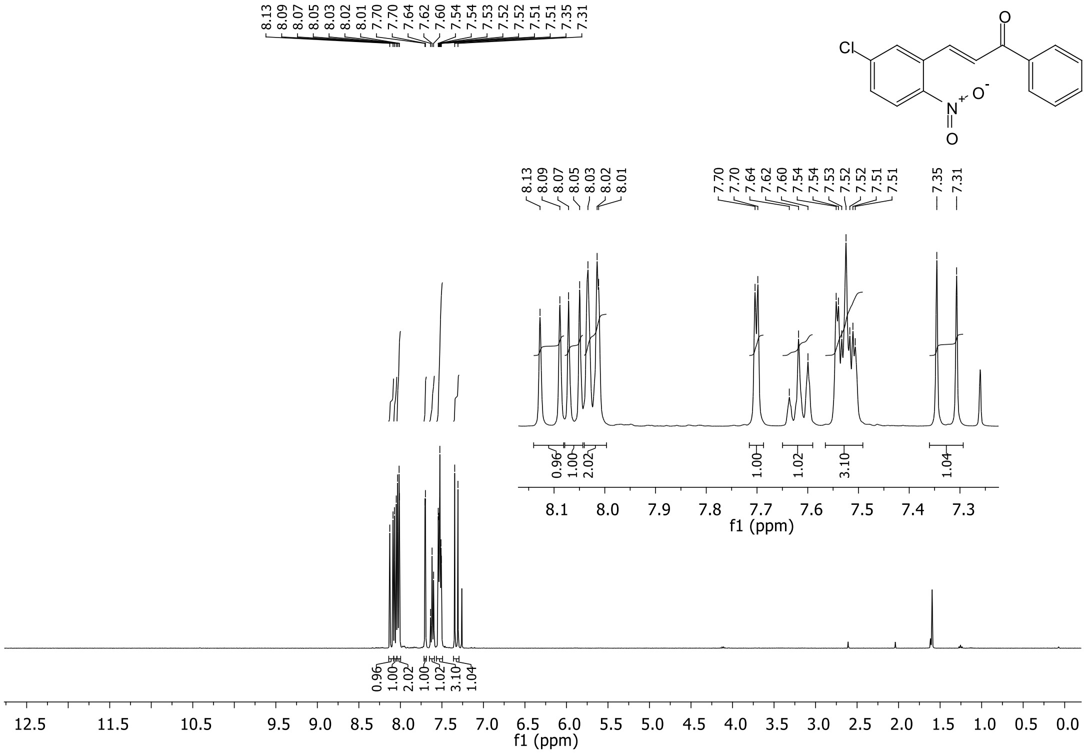

^{13}C {[1] H} NMR (100 MHz, $CDCl_3$) of 1q

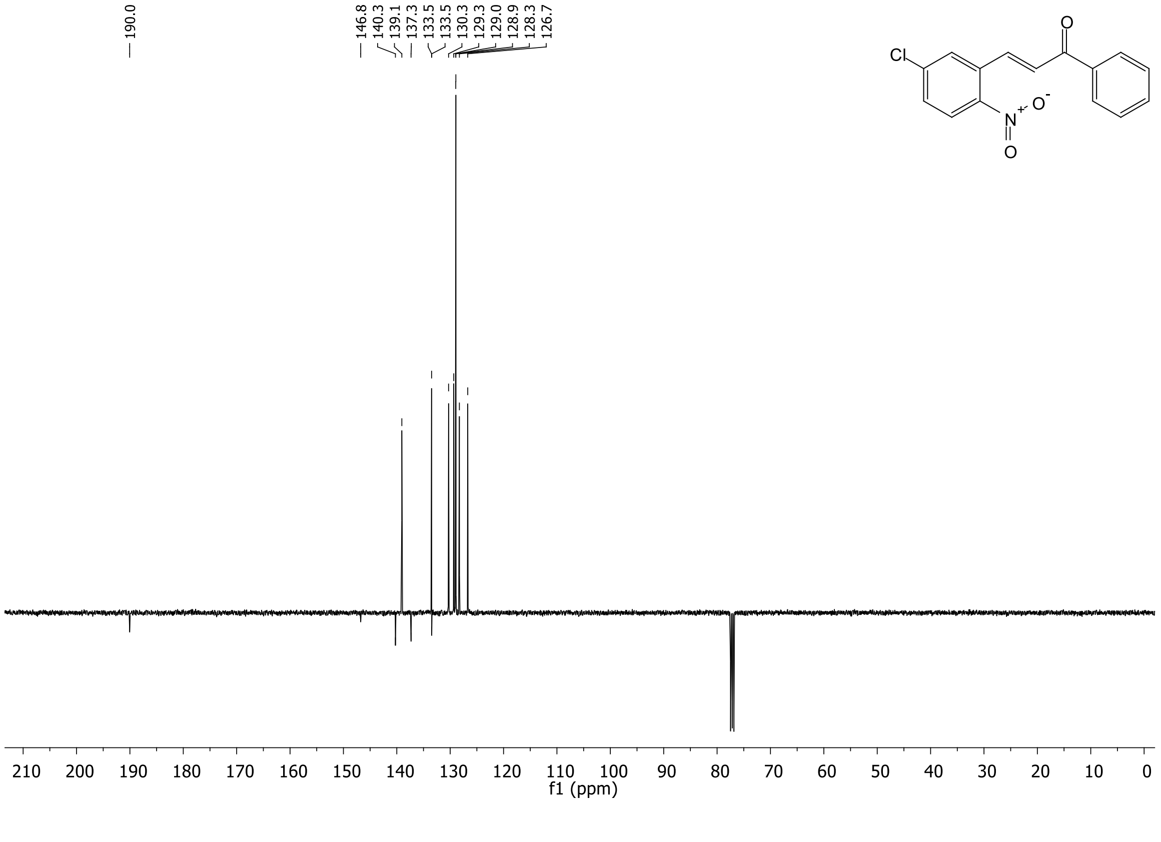

^{1}H NMR (400 MHz, $CDCl_3$) of 1r

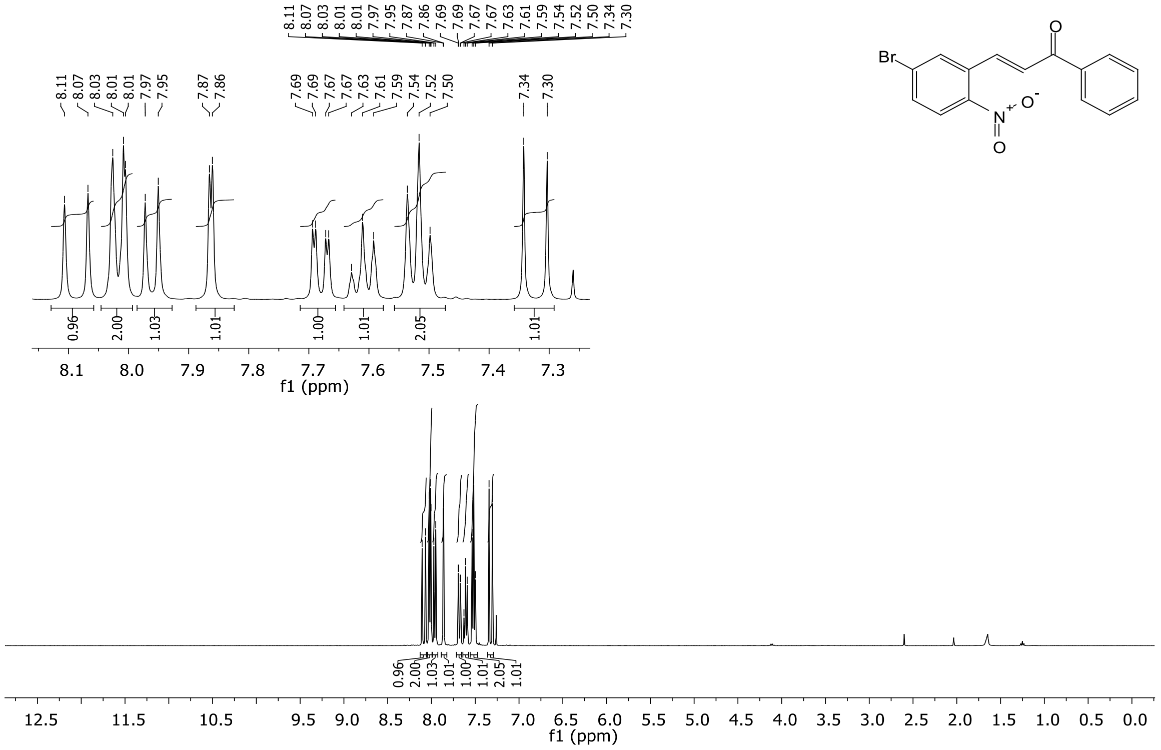

^{13}C {[1] H} NMR (100 MHz, $CDCl_3$) of 1r

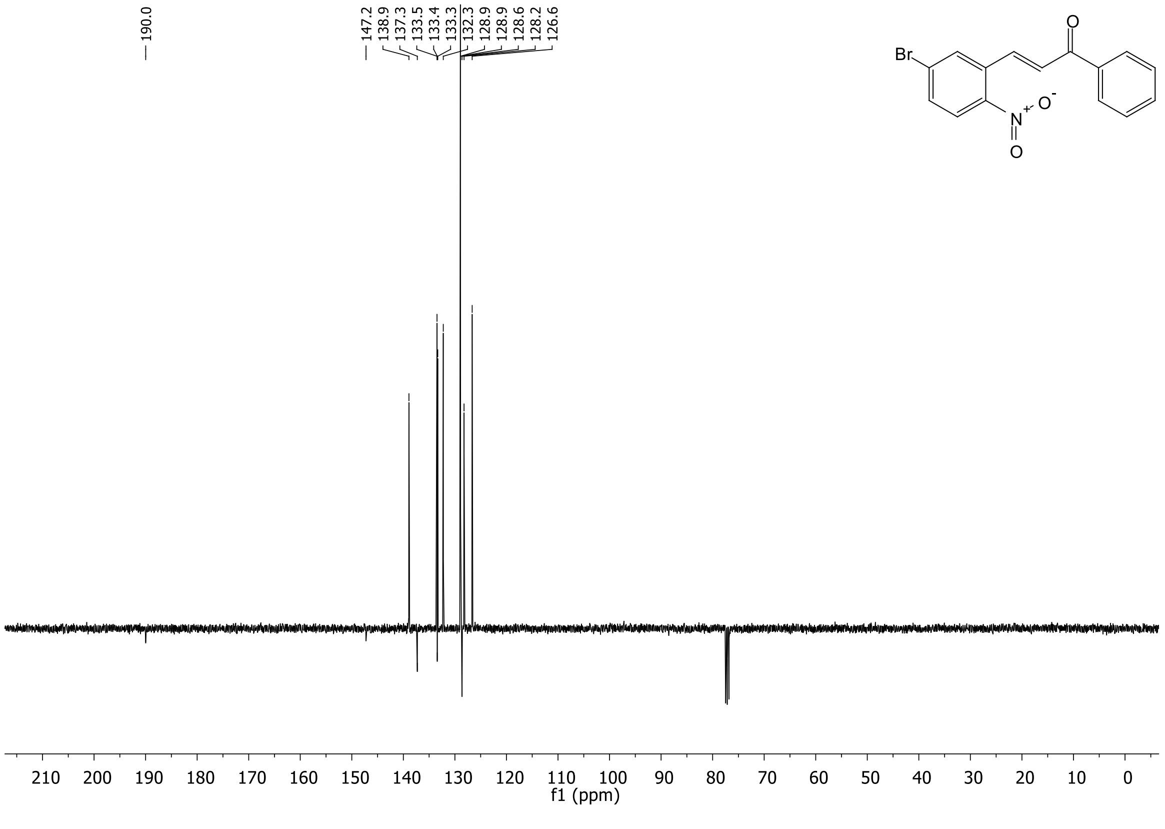

^{1}H NMR (400 MHz, $CDCl_3$) of 1s

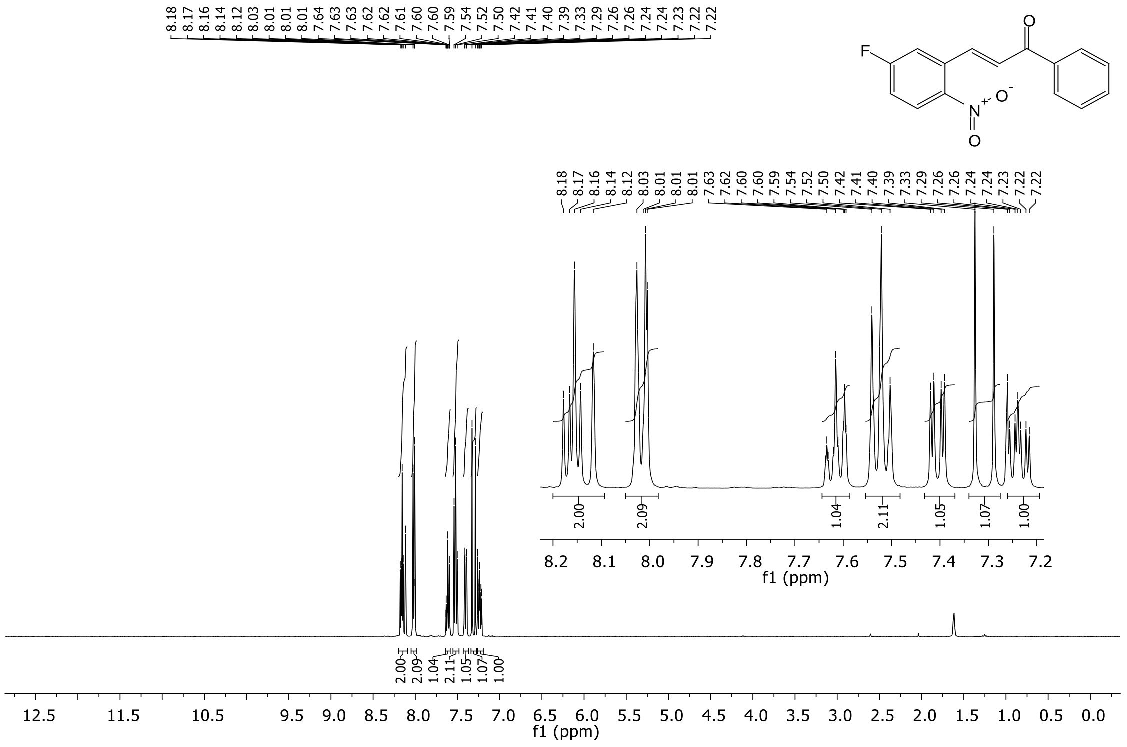

^{13}C {[1] H} NMR (100 MHz, $CDCl_3$) of 1s

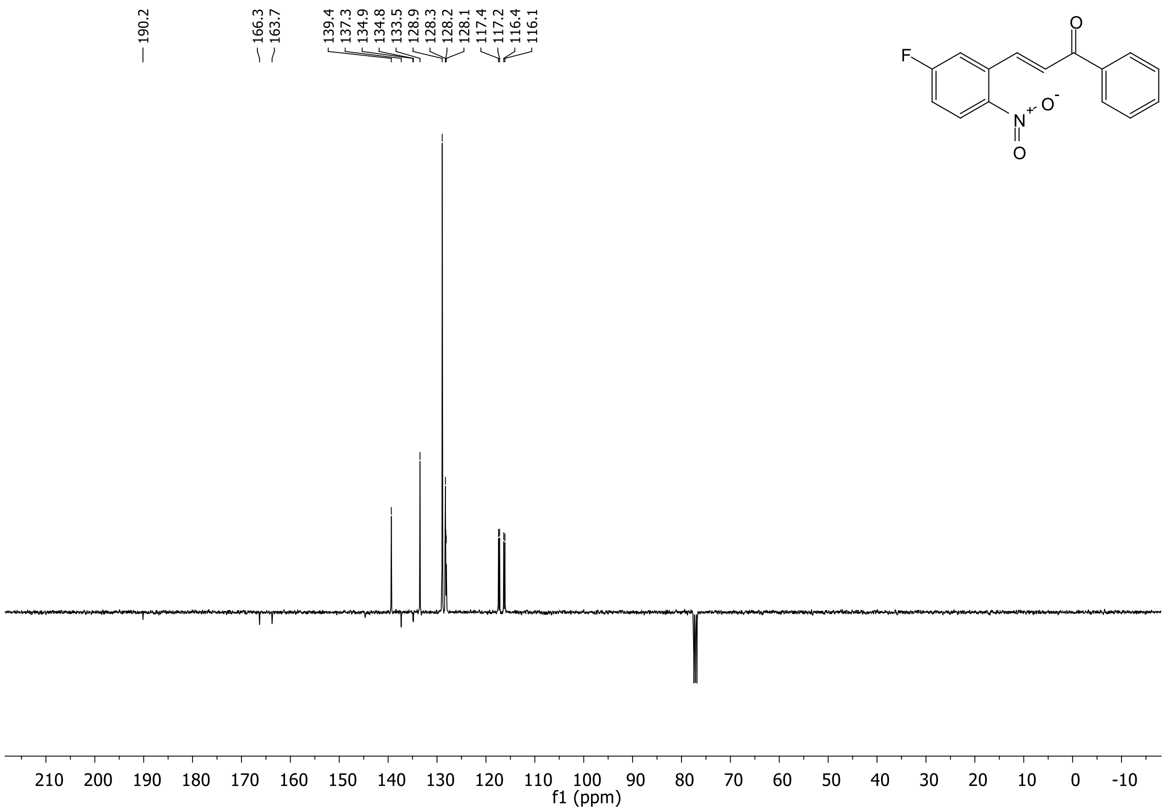

^{19}F NMR (376 MHz, $CDCl_3$) of 1s

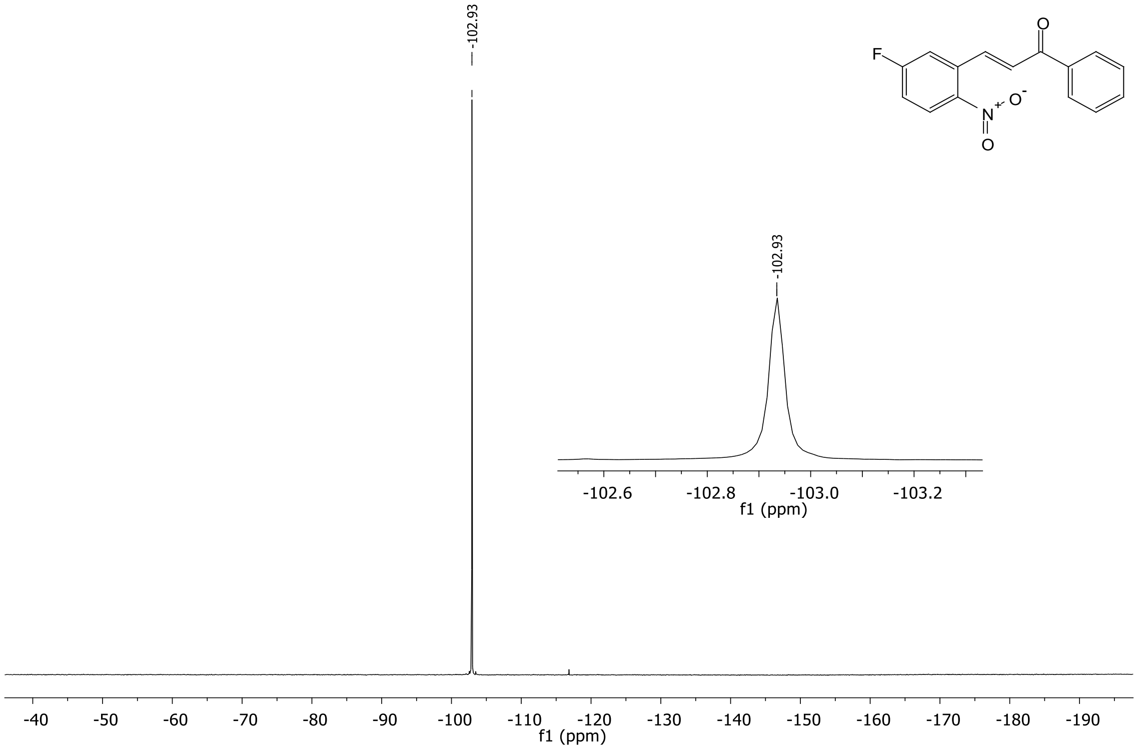

^{1}H NMR (400 MHz, $CDCl_3$) of 1t

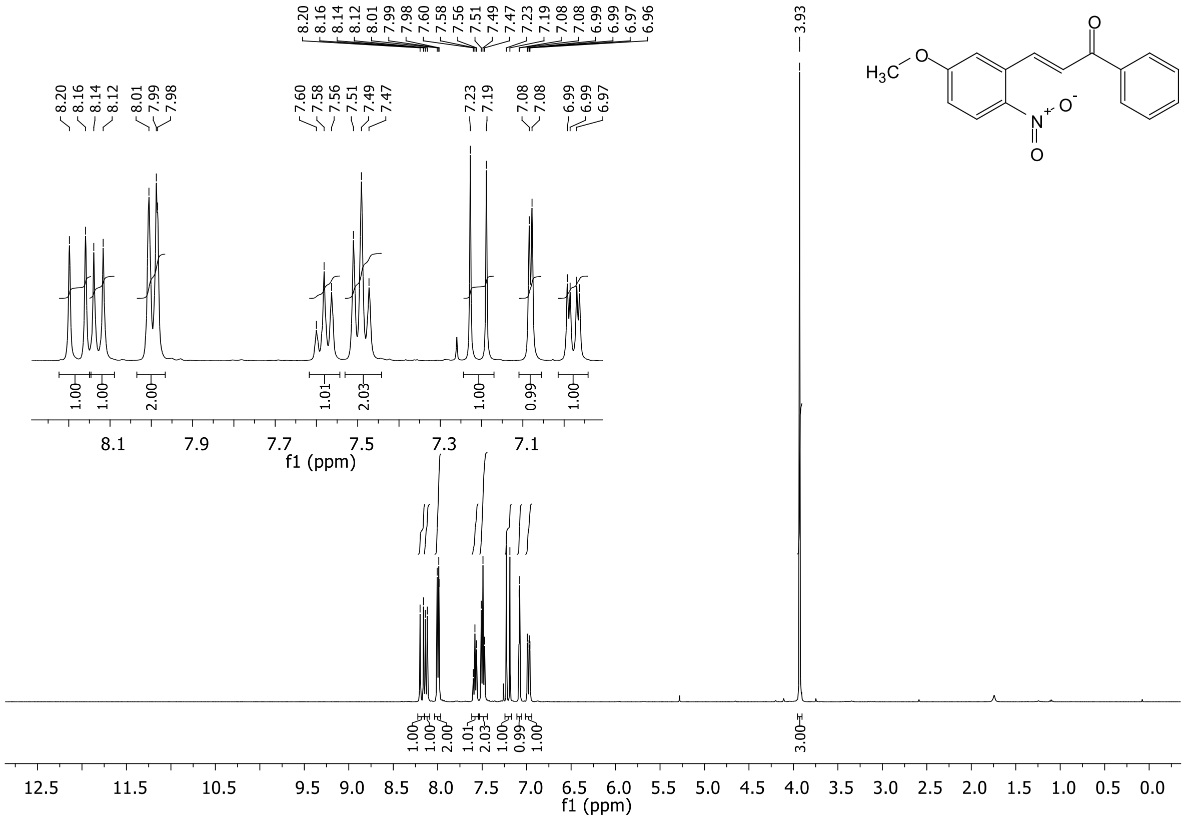

^{13}C {^{1} H} NMR (100 MHz, $CDCl_3$) of 1t

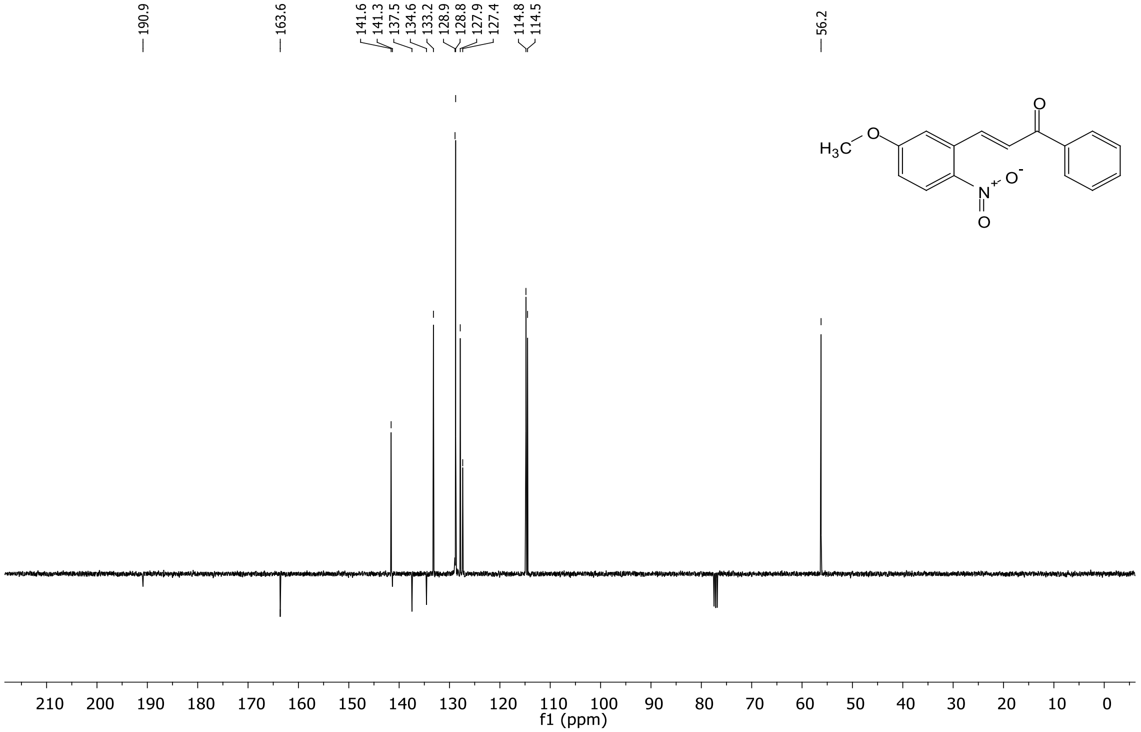

^{1}**H NMR (400 MHz, CDCl$_3$) of 1u**

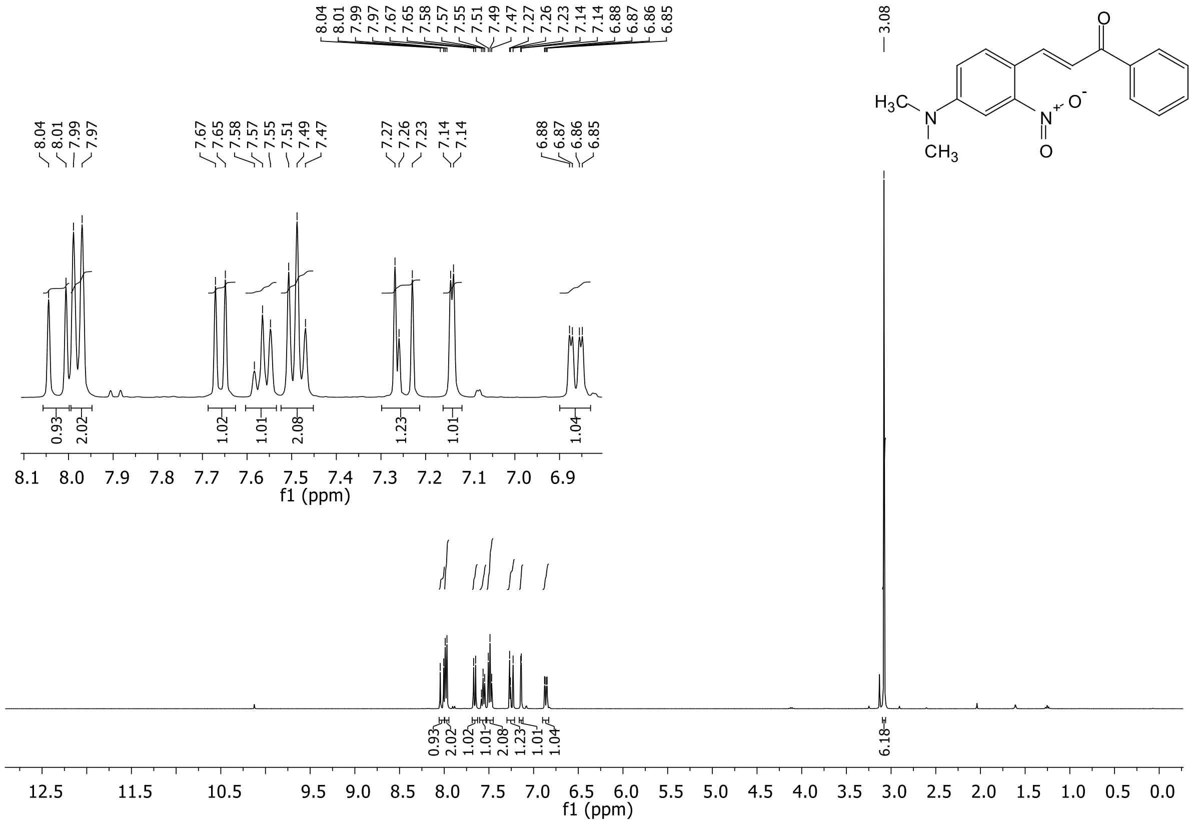

^{13}**C {^{1} H} NMR (100 MHz, CDCl$_3$) of 1u**

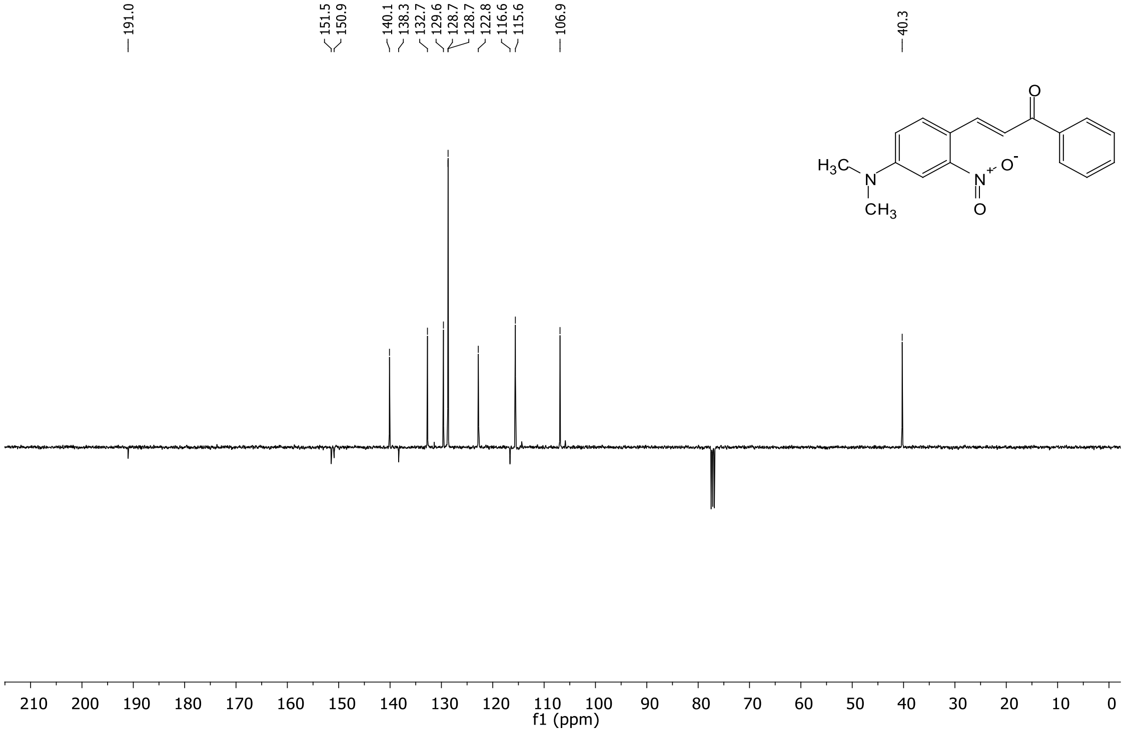

^{1}H NMR (400 MHz, $CDCl_3$) of 1v

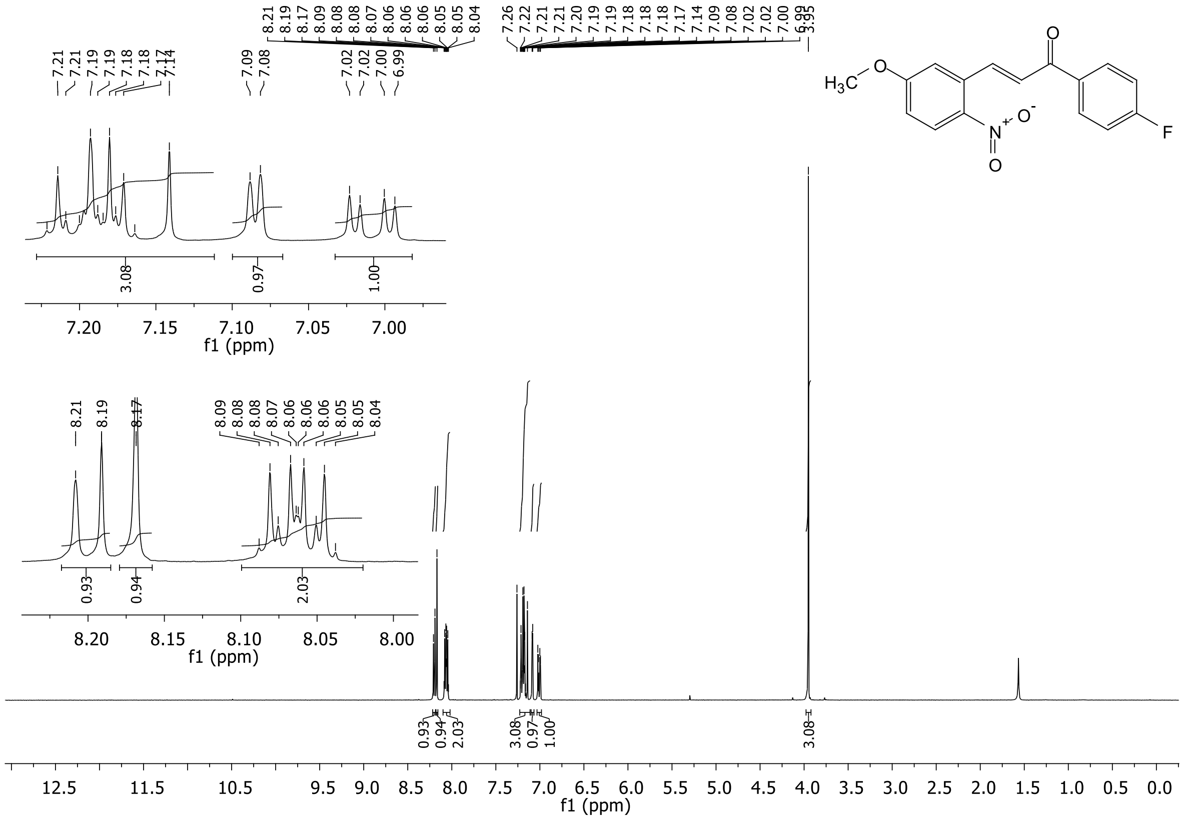

^{13}C {^{1} H} NMR (100 MHz, $CDCl_3$) of 1v

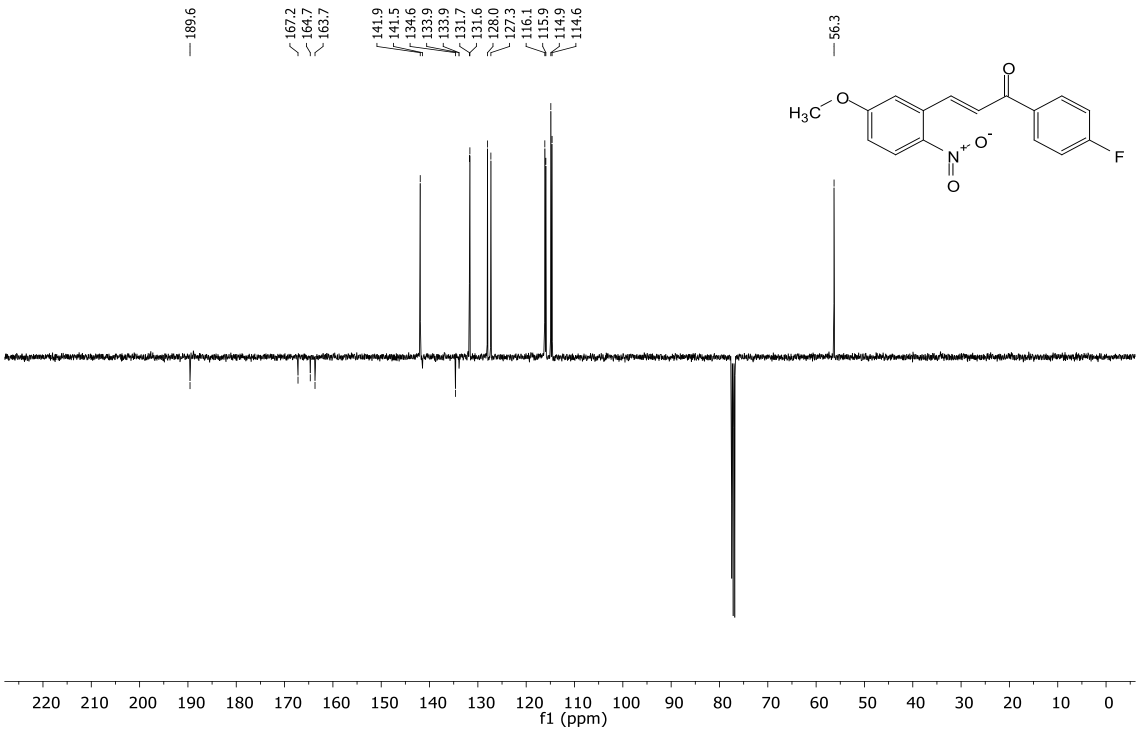

^{19}F NMR (376 MHz, CDCl$_3$) of 1v

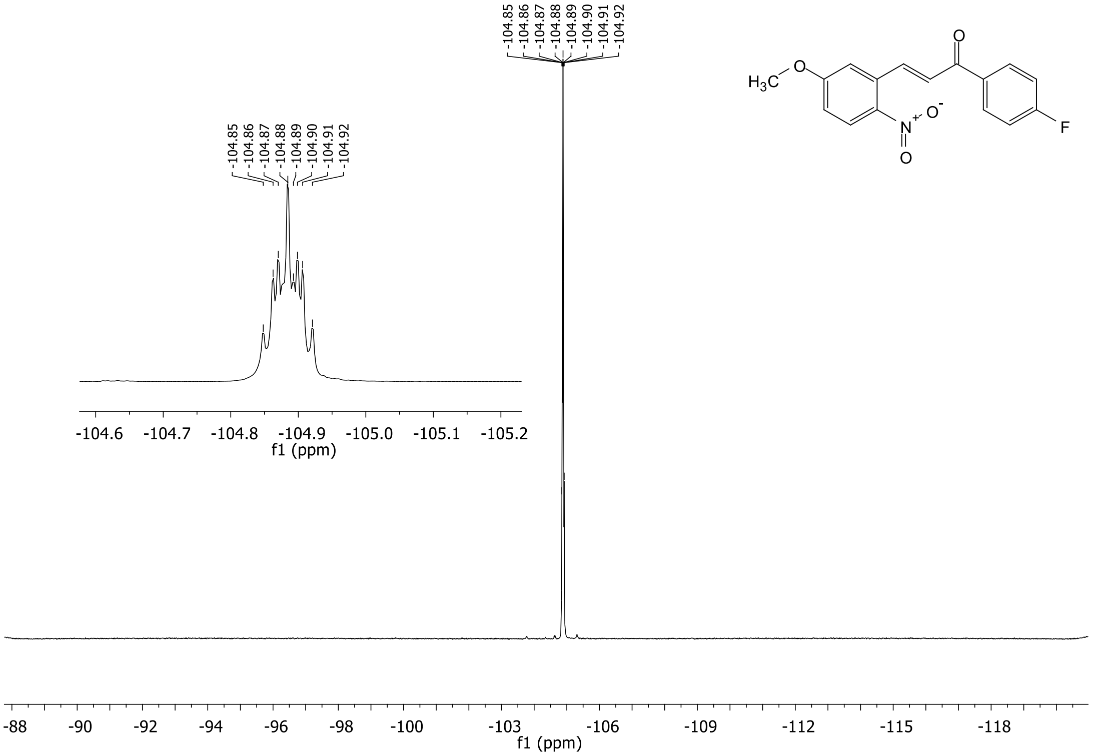

^{1}H NMR (400 MHz, $CDCl_3$) of 1w

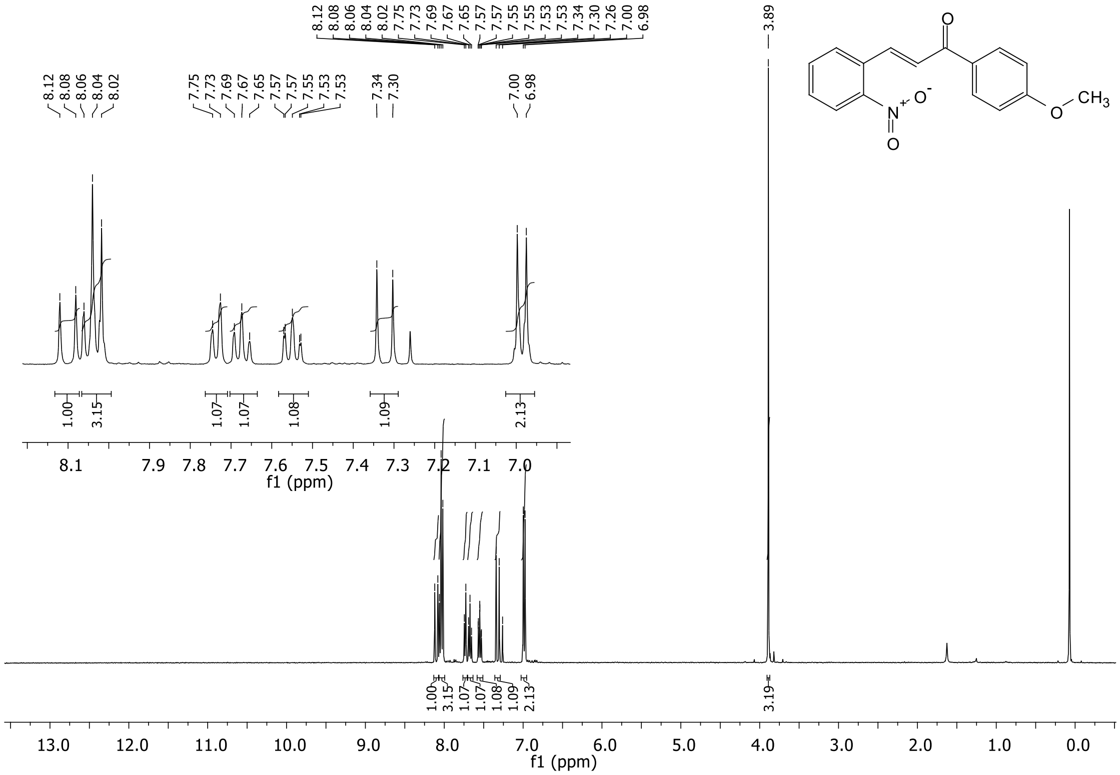

^{13}C {^{1} H} NMR (100 MHz, $CDCl_3$) of 1w

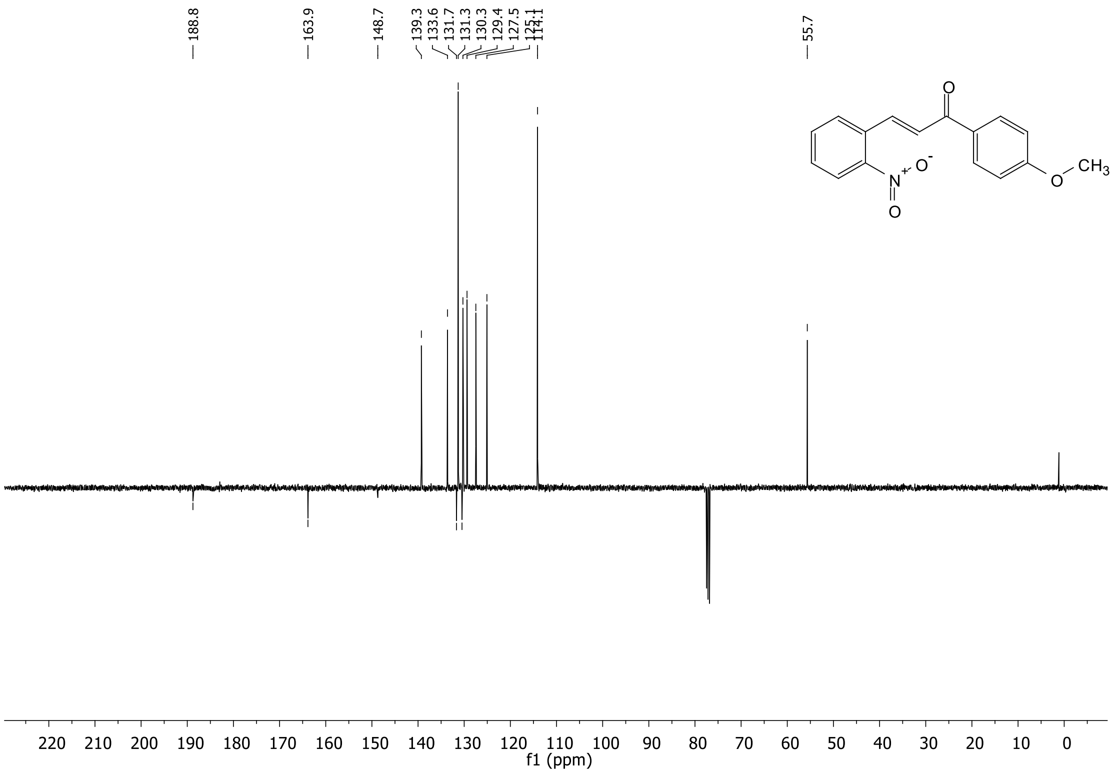

^{1}H NMR (400 MHz, $CDCl_3$) of 1x

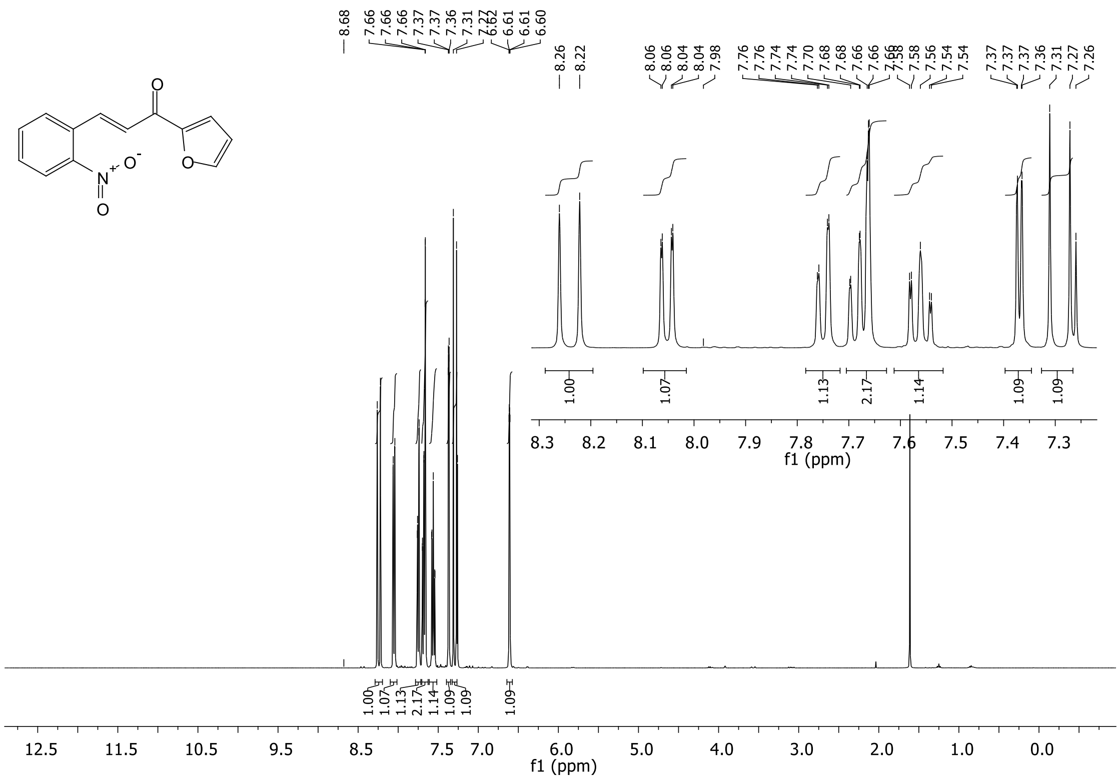

^{13}C {[1] H} NMR (100 MHz, $CDCl_3$) of 1x

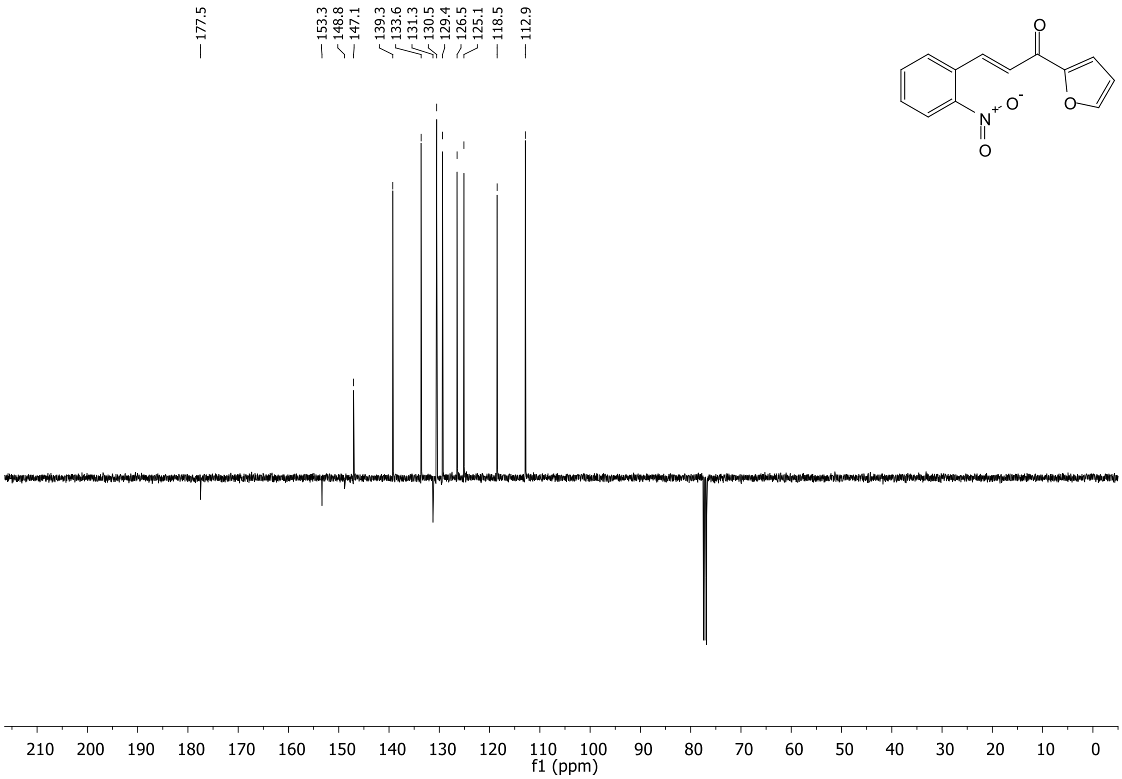

^{1}H NMR (400 MHz, $CDCl_3$) of 2a

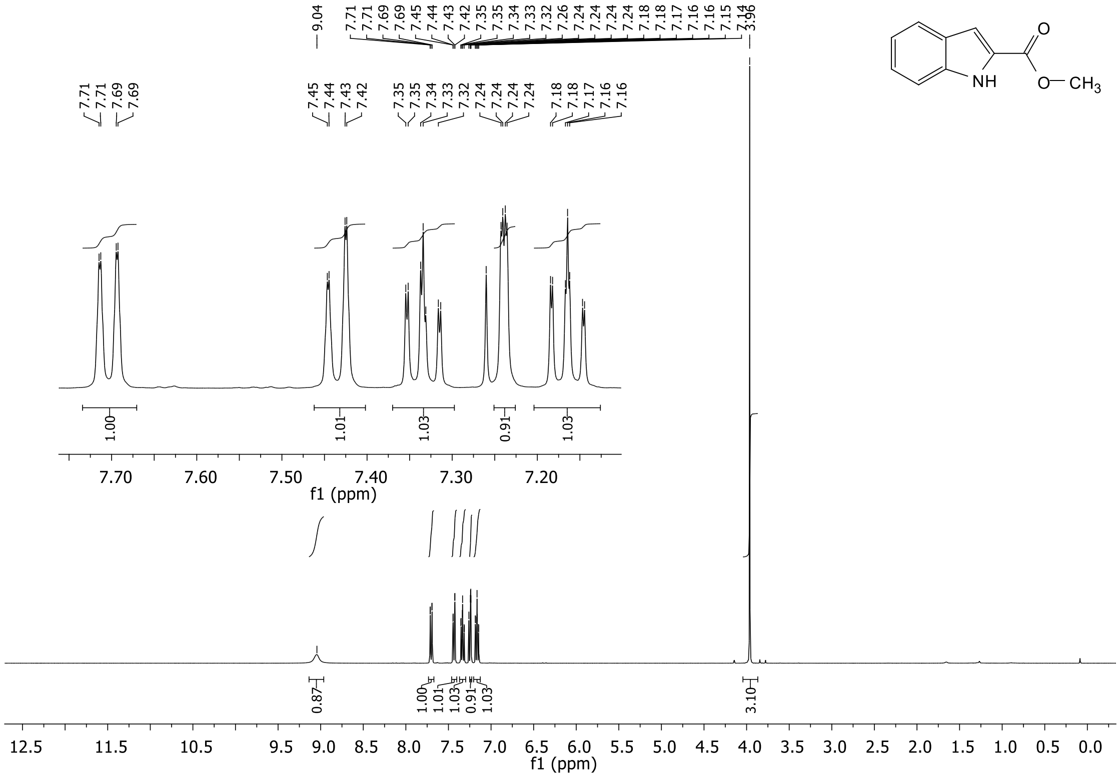

^{13}C {^{1} H} NMR (100 MHz, $CDCl_3$) of 2a

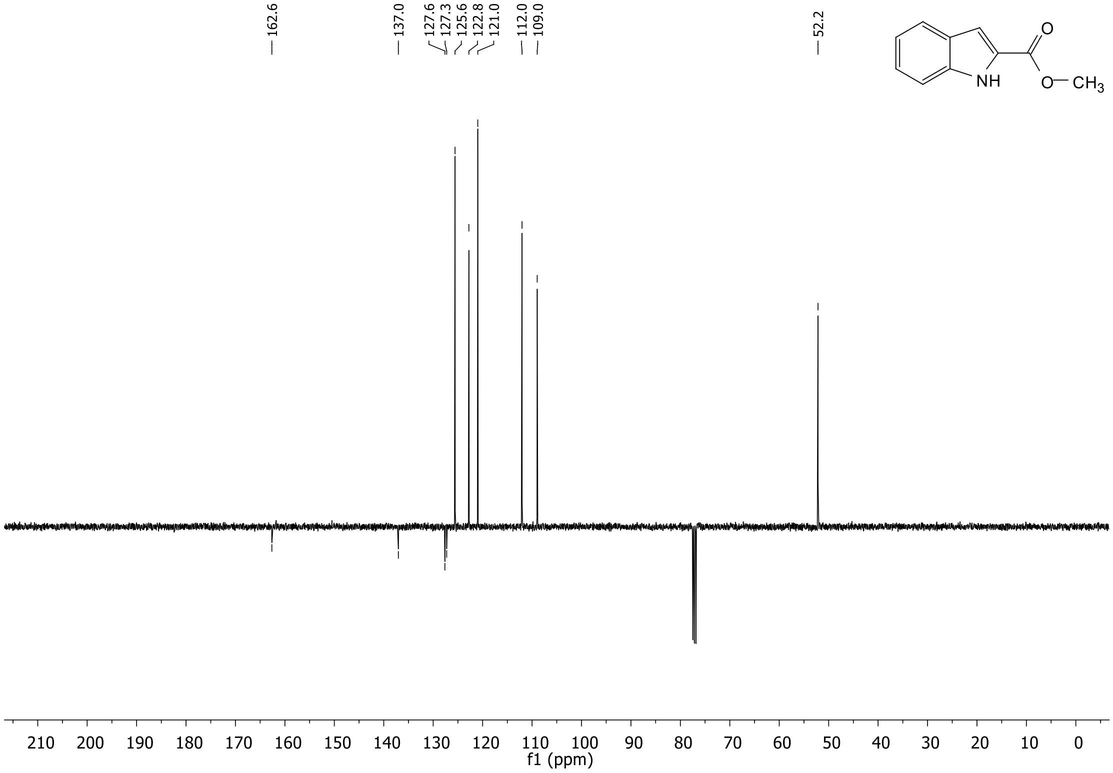

^{1}H NMR (400 MHz, $CDCl_3$) of 2b

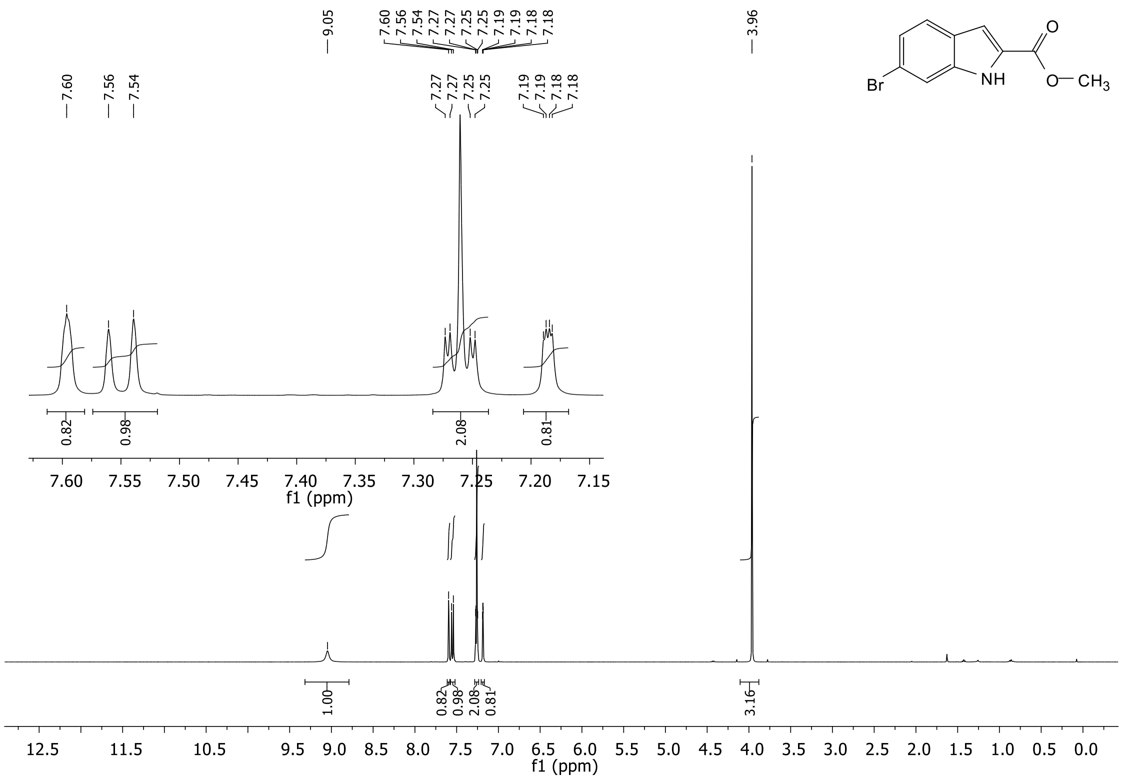

^{13}C {[1] H} NMR (100 MHz, $CDCl_3$) of 2b

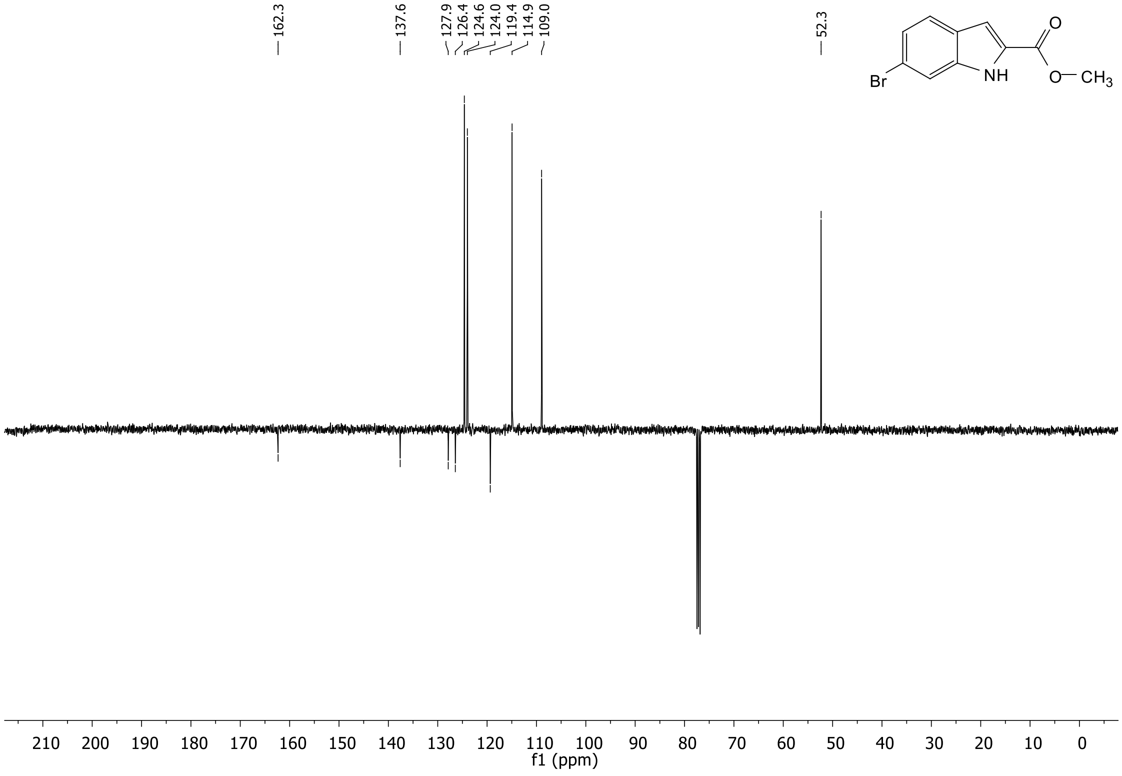

^{1}H NMR (400 MHz, $CDCl_3$) of 2c

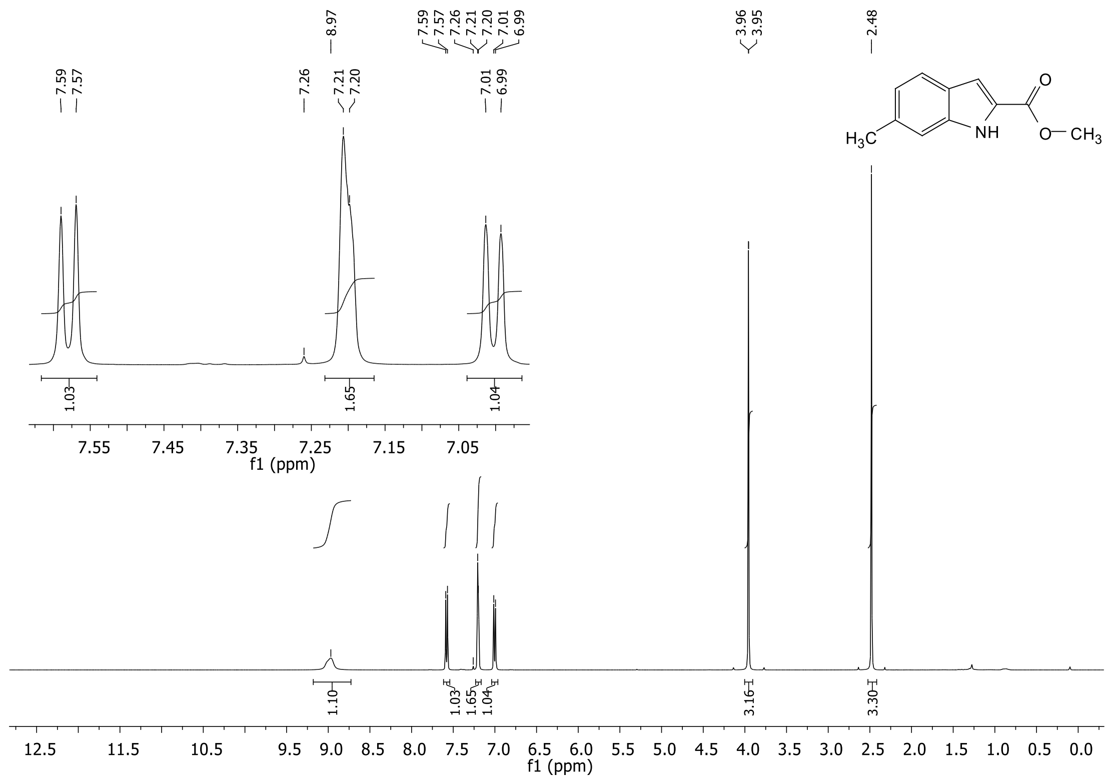

^{13}C {[1] H} NMR (100 MHz, $CDCl_3$) of 2c

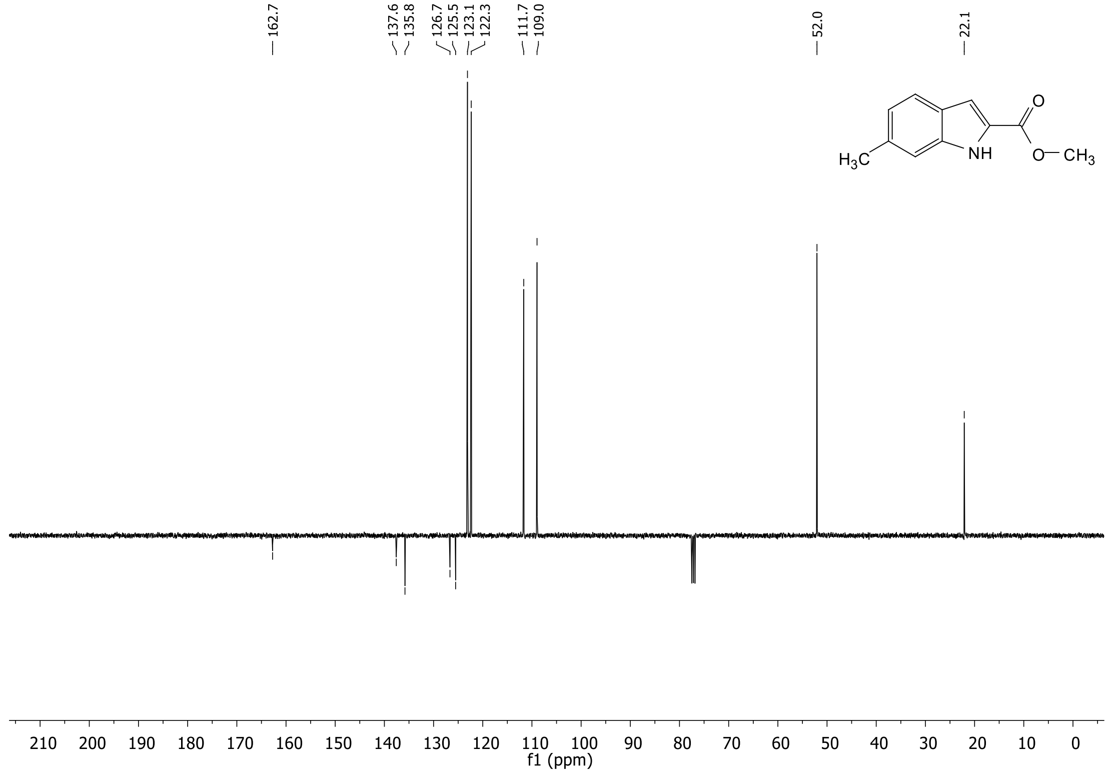

^{1}H NMR (400 MHz, $CDCl_3$) of 2d

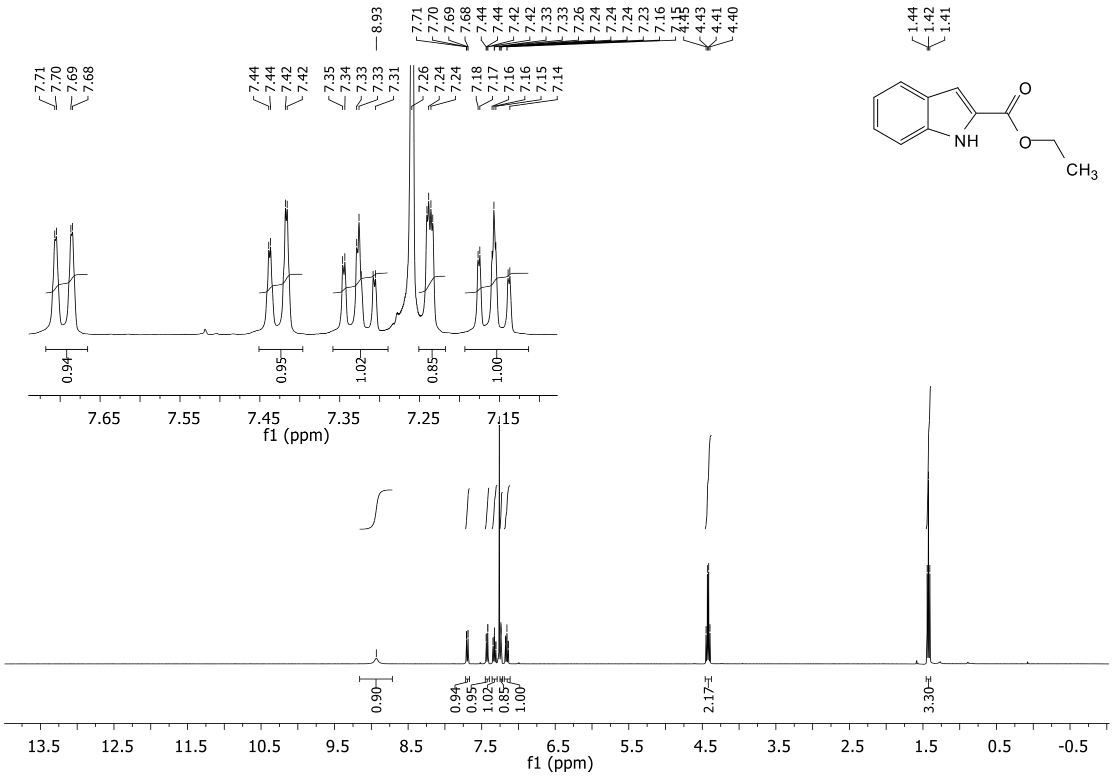

^{13}C {^{1} H} NMR (100 MHz, $CDCl_3$) of 2d

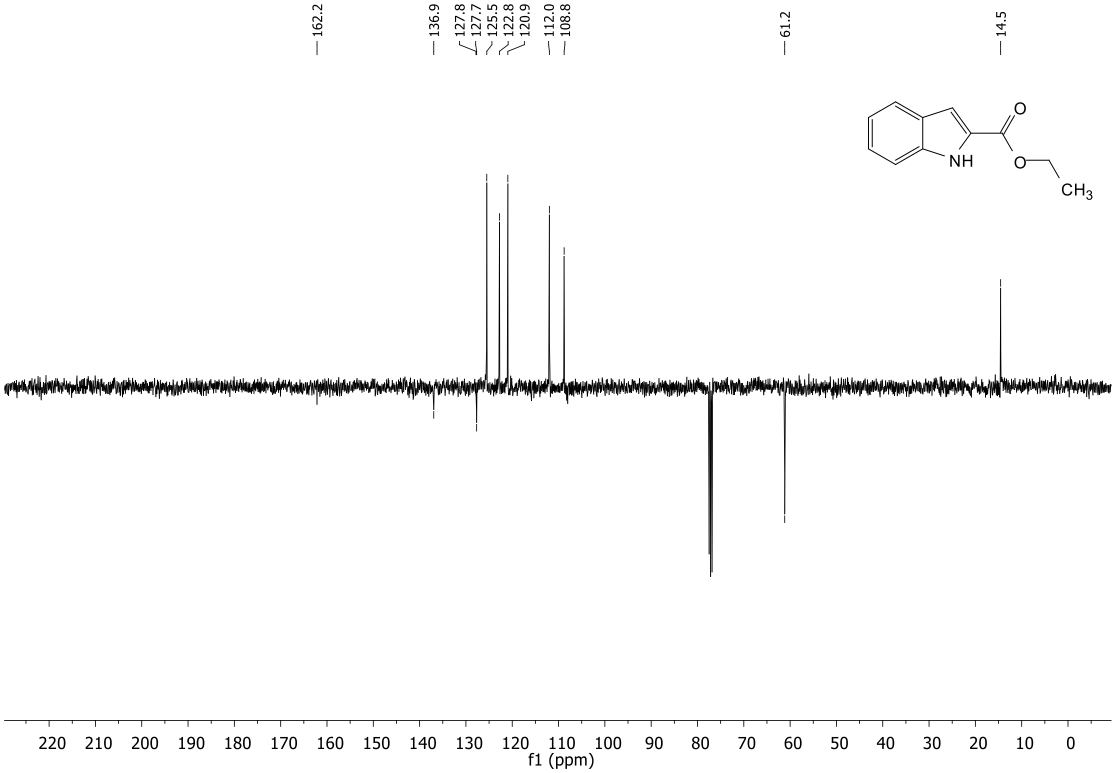

^{1}H NMR (400 MHz, $CDCl_3$) of 2e

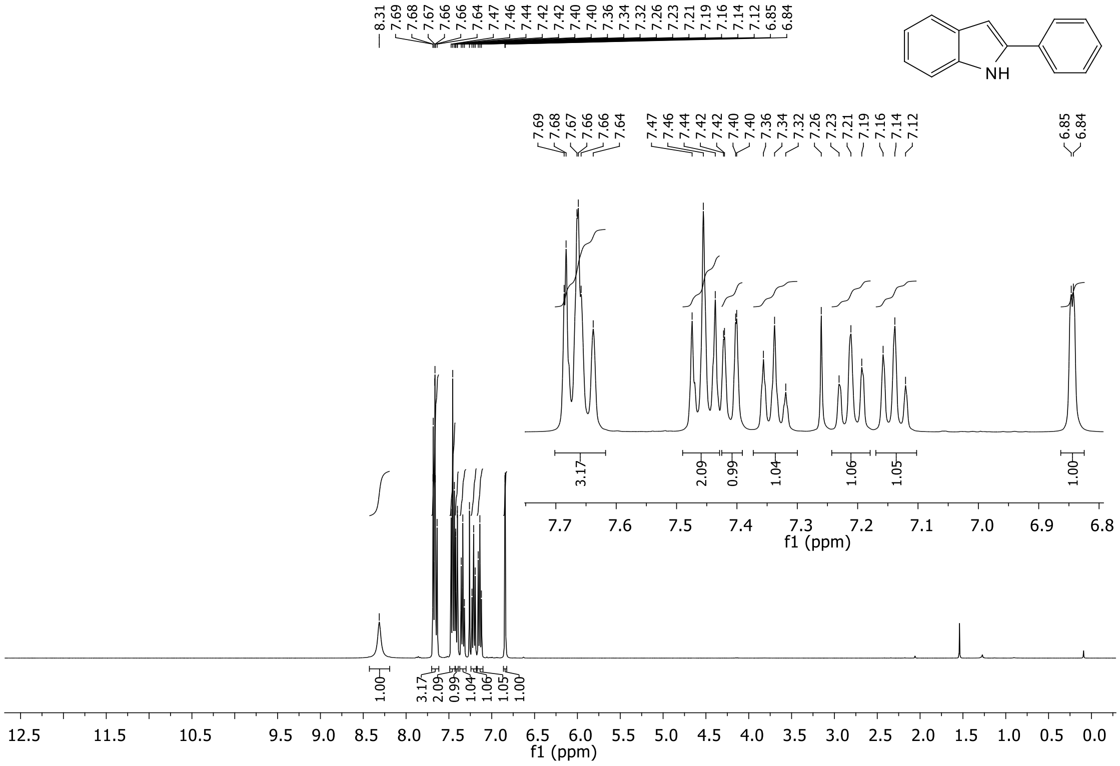

^{13}C {^{1} H} NMR (100 MHz, $CDCl_3$) of 2e

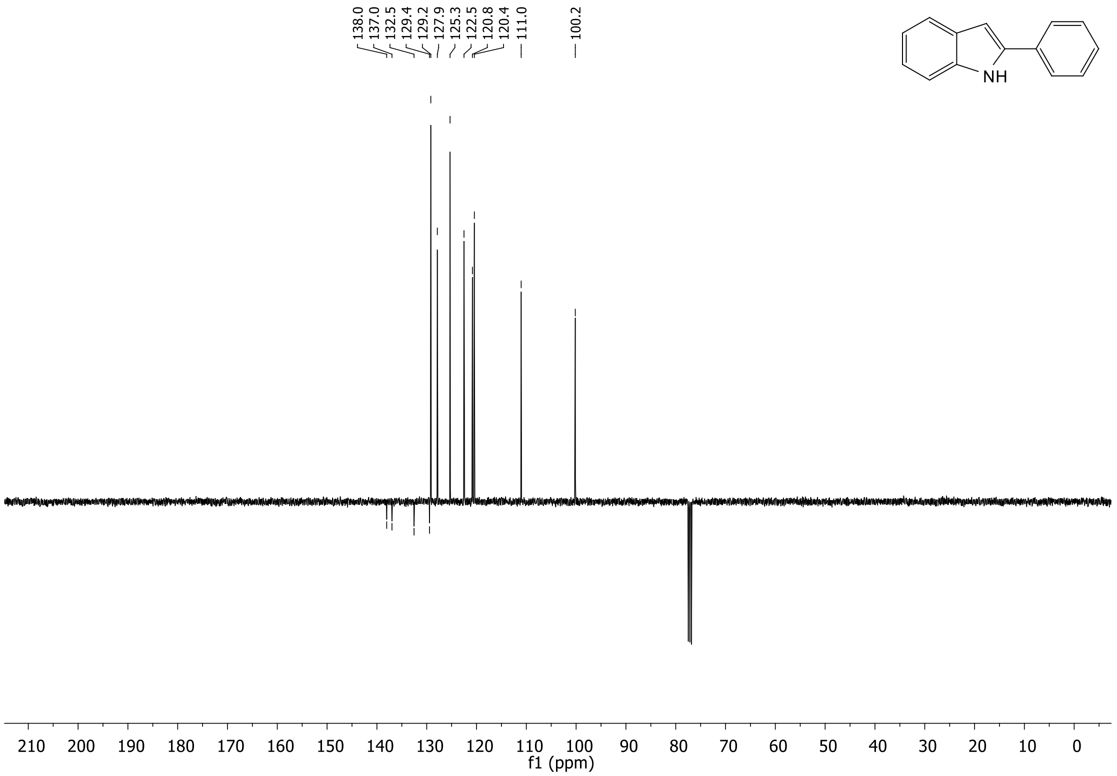

^{1}H NMR (400 MHz, $CDCl_3$) of 2f

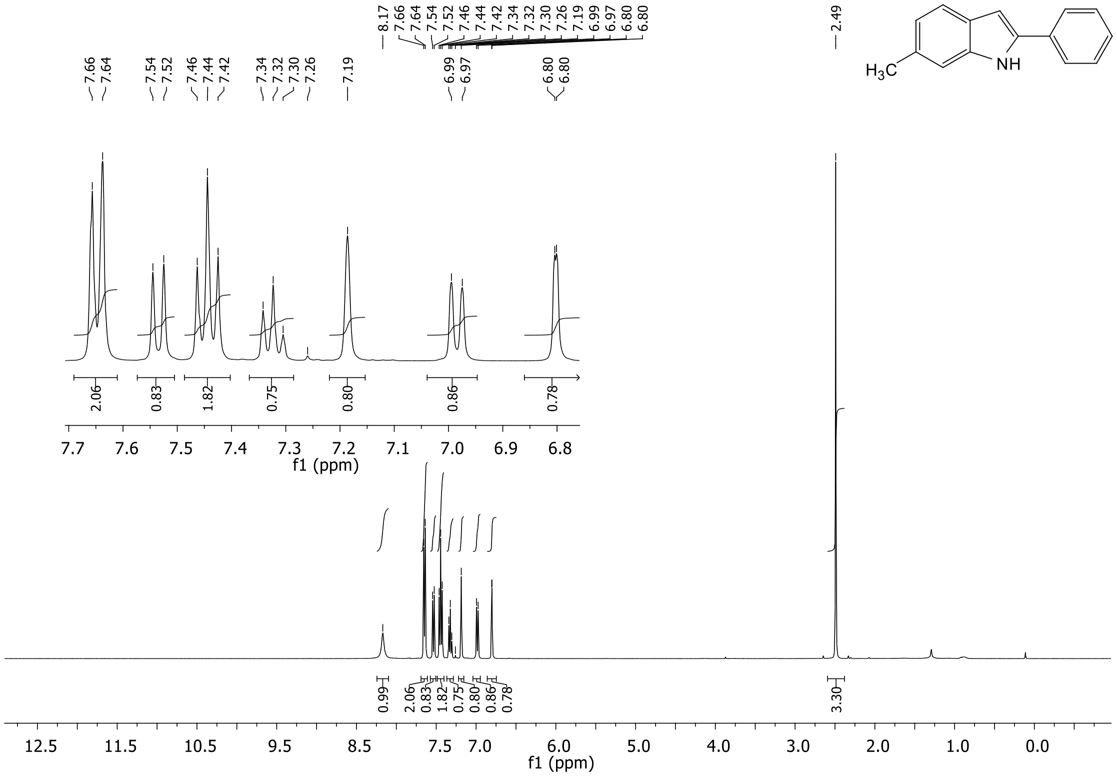

^{13}C {[1] H} NMR (100 MHz, $CDCl_3$) of 2f

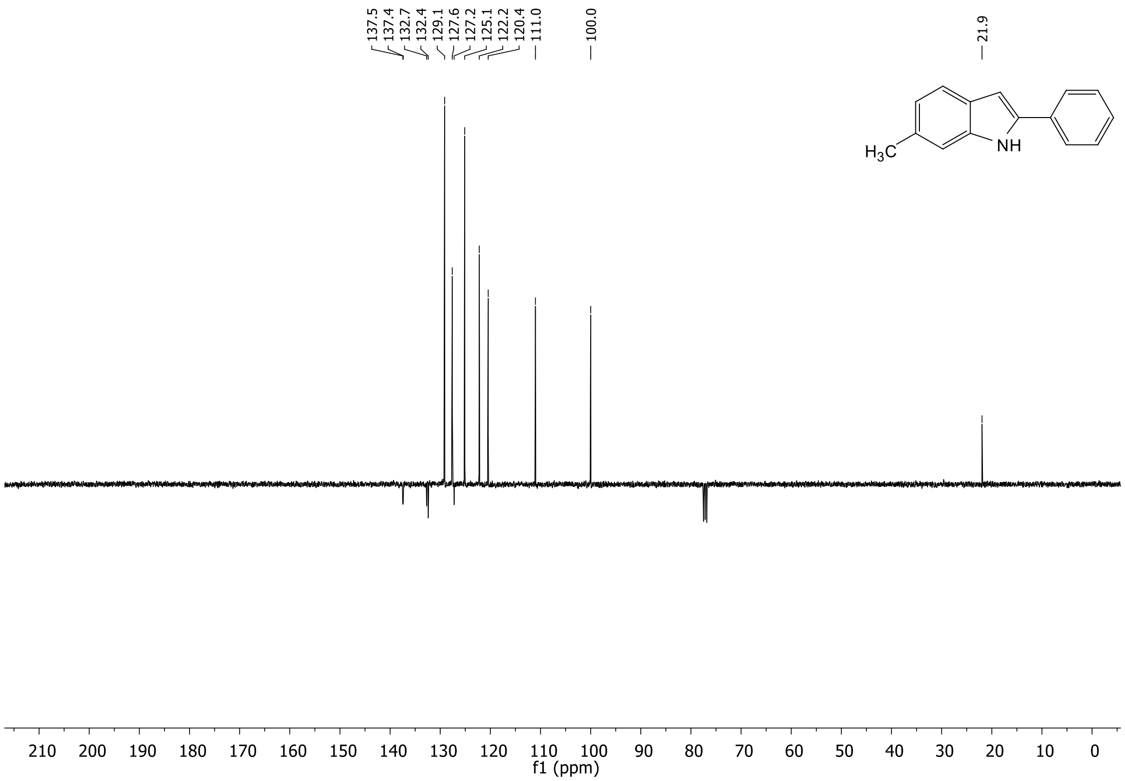

^{1}H NMR (400 MHz, $CDCl_3$) of 2g

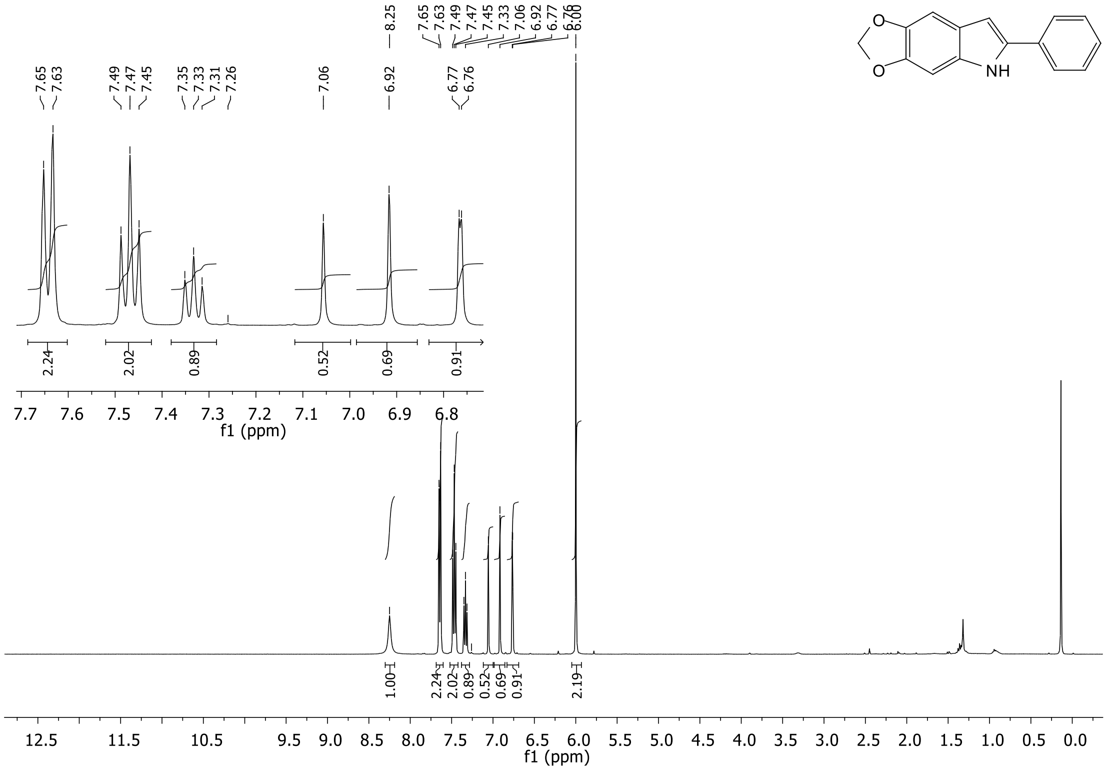

^{13}C {^{1} H} NMR (100 MHz, $CDCl_3$) of 2g

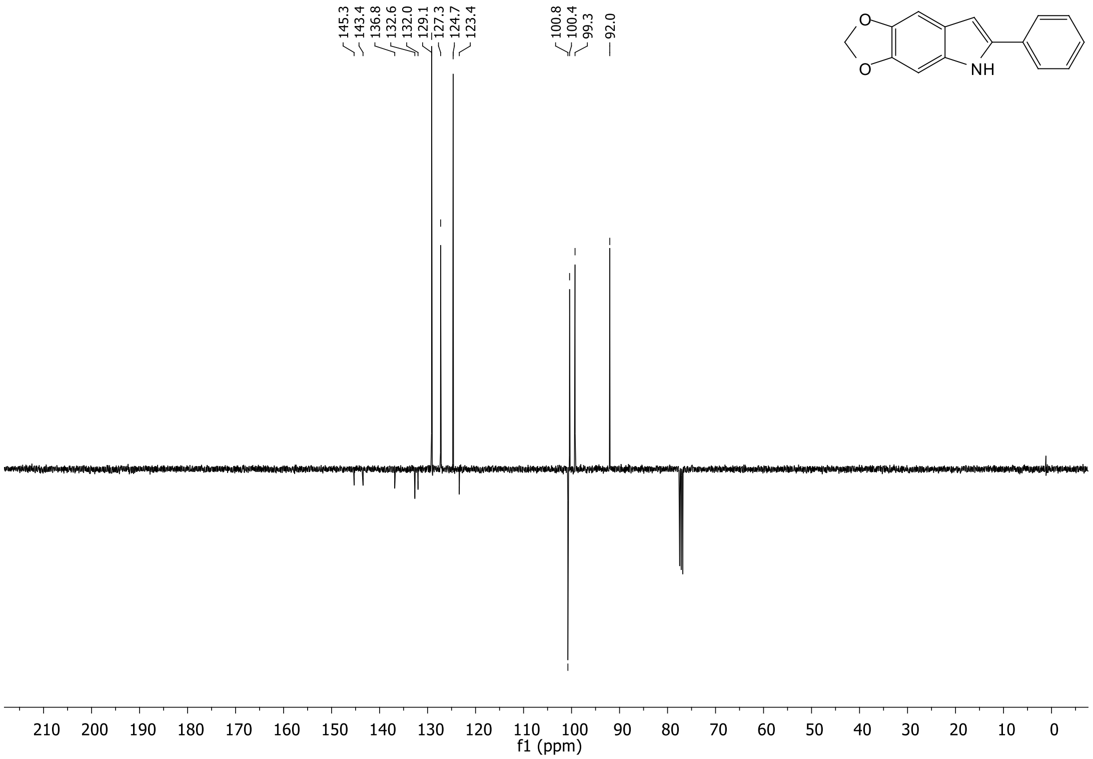

^{1}H NMR (400 MHz, DMSO-d_6) of 2h

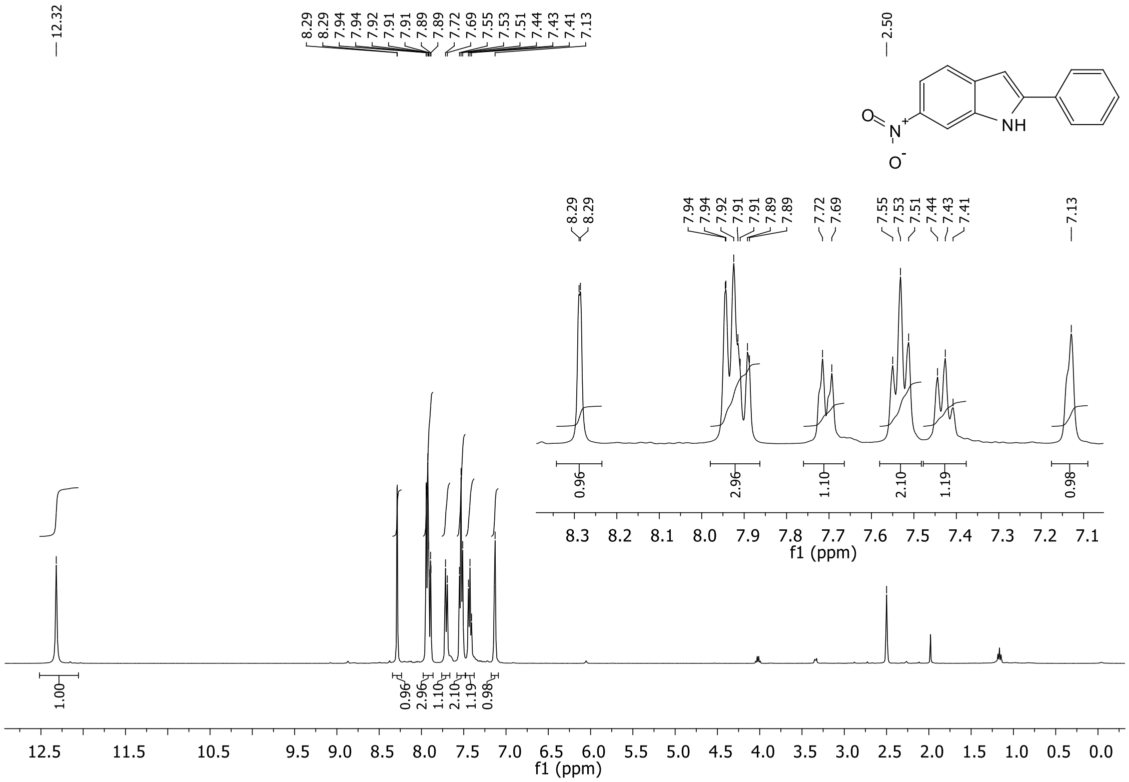

^{13}C {^{1}H} NMR (100 MHz, DMSO-d_6) of 2h

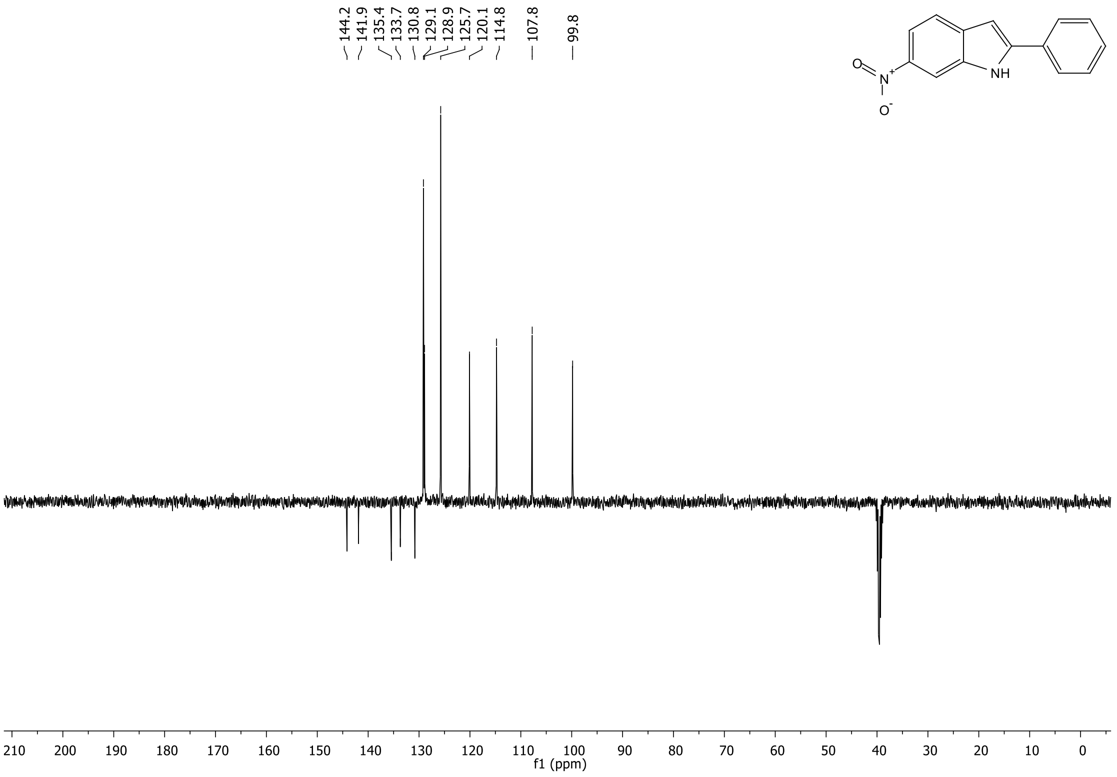

^{1}H NMR (400 MHz, DMSO-d_6) of 2i

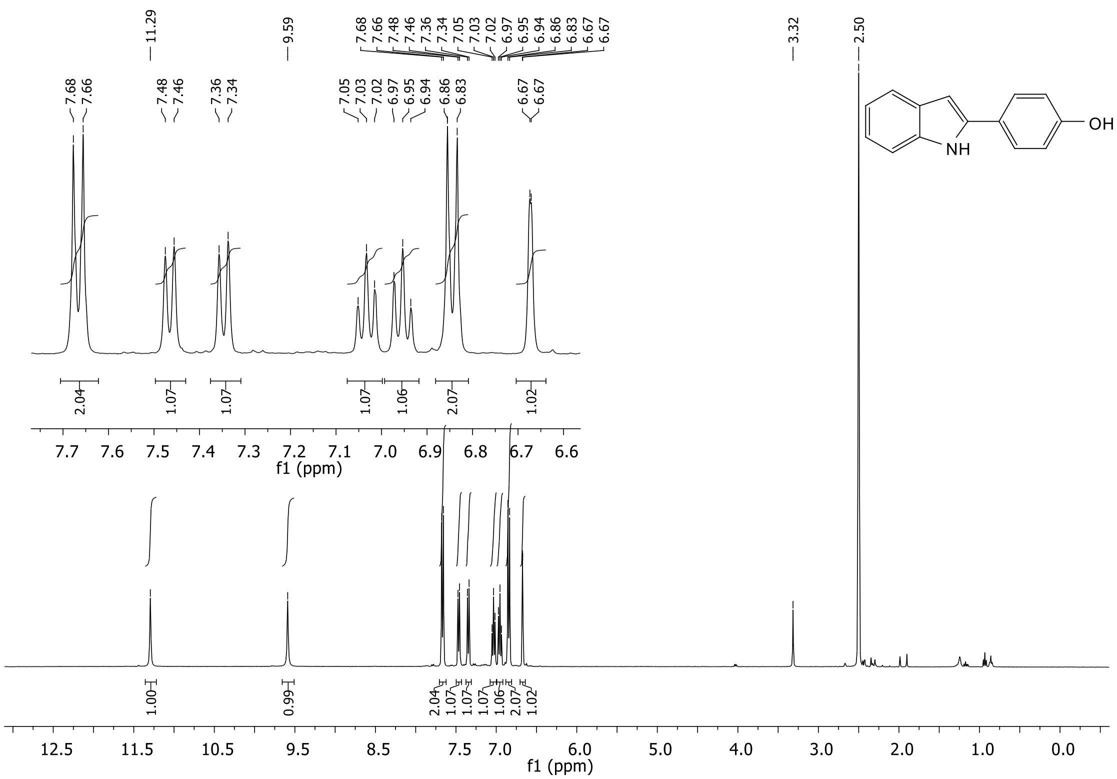

^{13}C {^{1} H} NMR (100 MHz, DMSO-d_6) of 2i

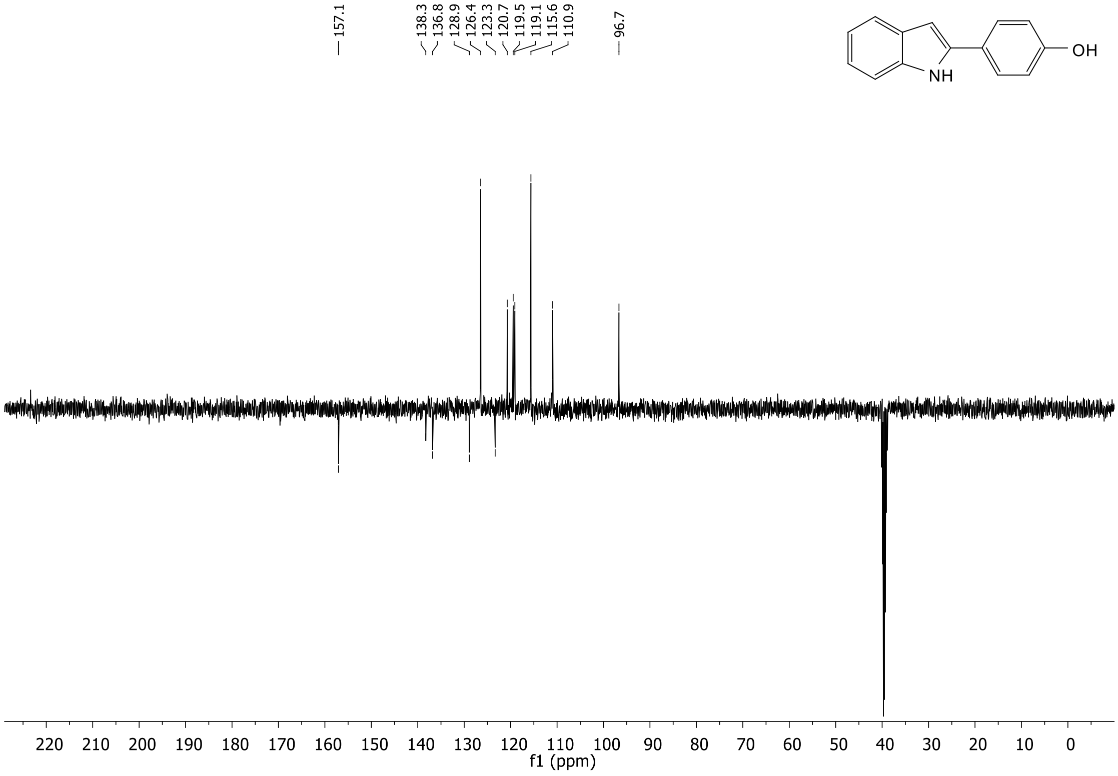

^{1}H NMR (400 MHz, DMSO-d_6) of 2i'

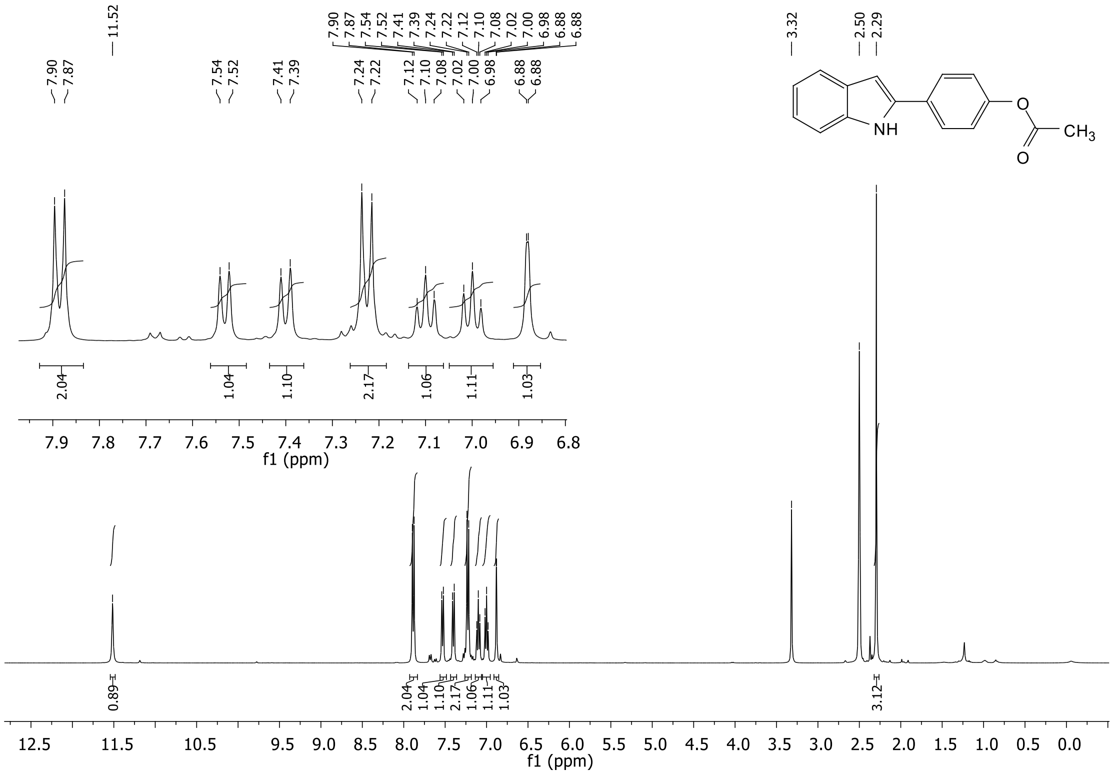

^{13}C {[1] H} NMR (100 MHz, DMSO-d_6) of 2i'

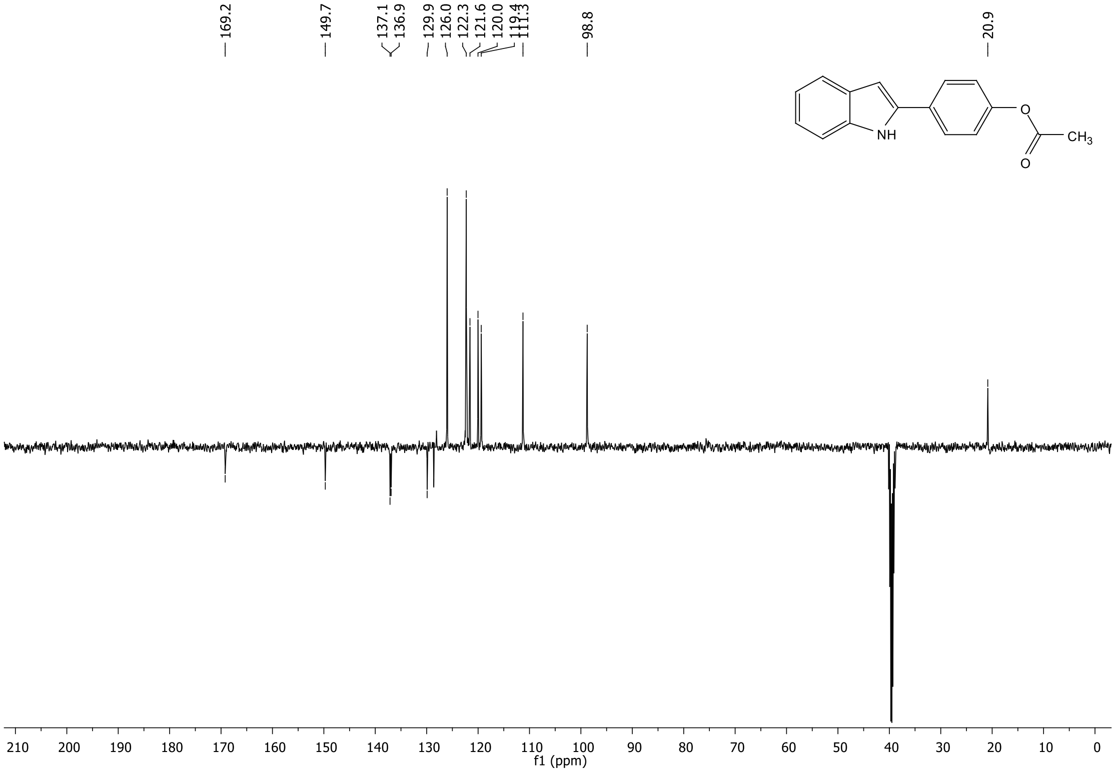

^{1}H NMR (400 MHz, DMSO-d_6) of 2j

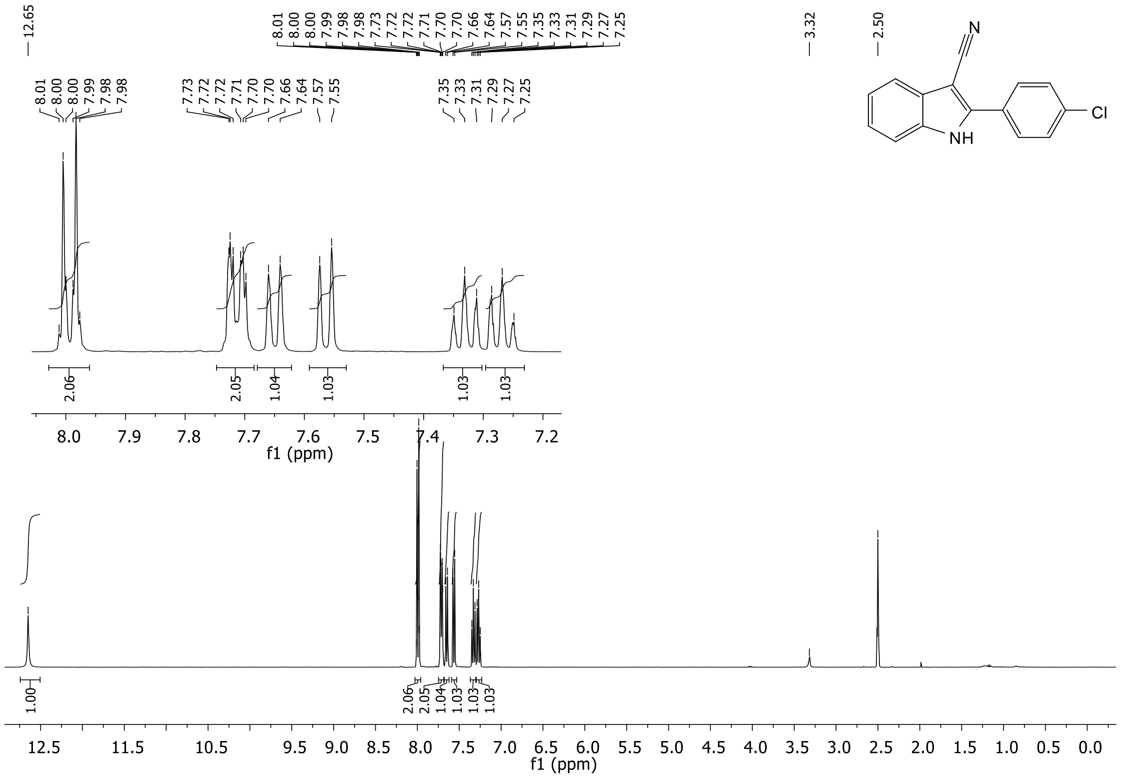

^{13}C {[1] H} NMR (100 MHz, DMSO-d_6) of 2j

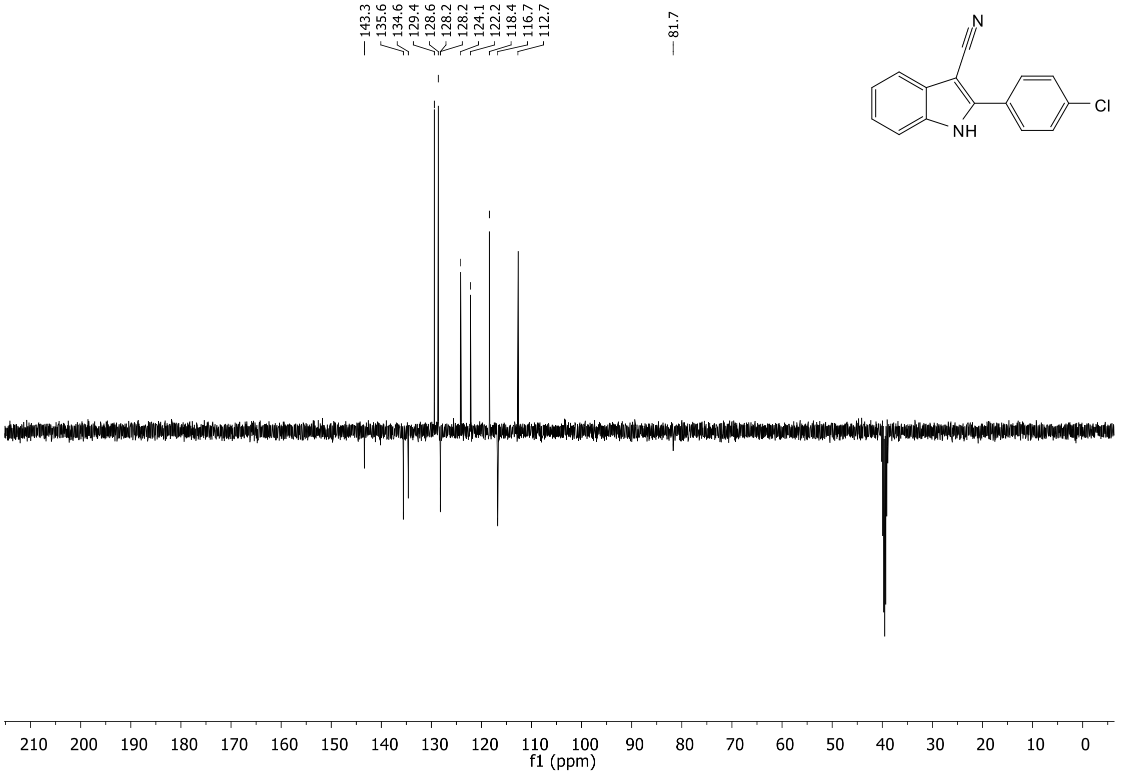

^1H NMR (400 MHz, DMSO-d_6) of 2k

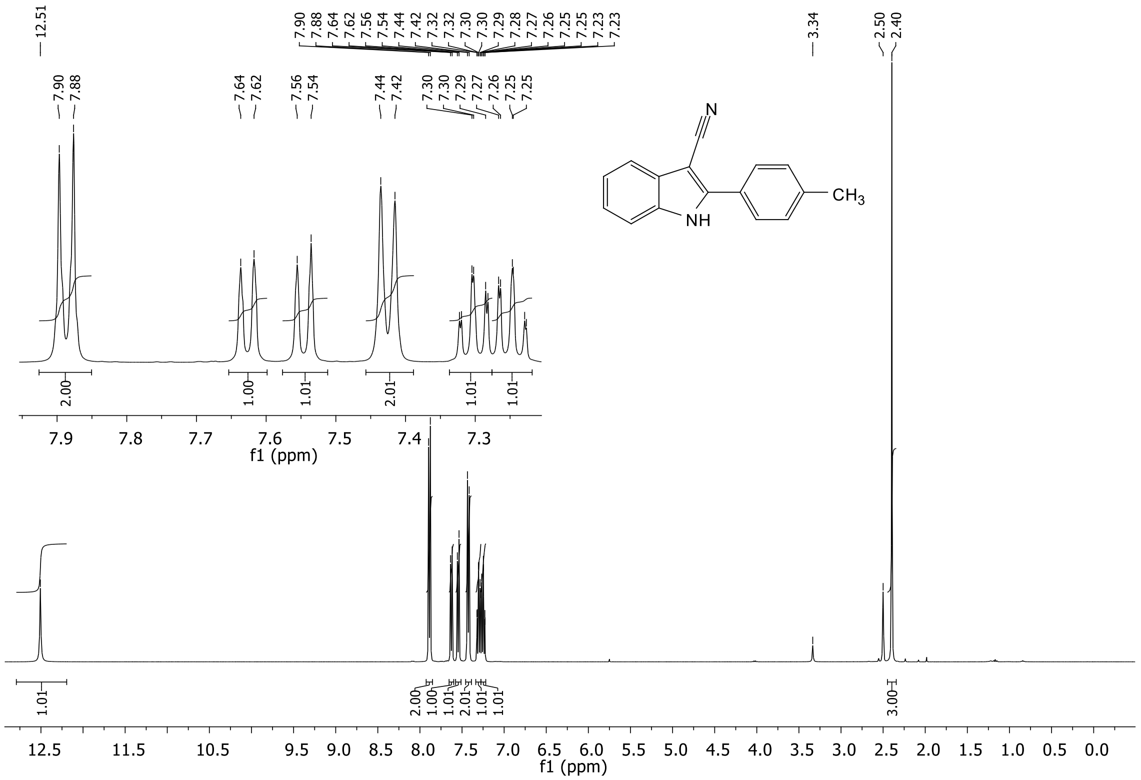

$^{13}C\{^1H\}$ NMR (100 MHz, DMSO-d_6) of 2k

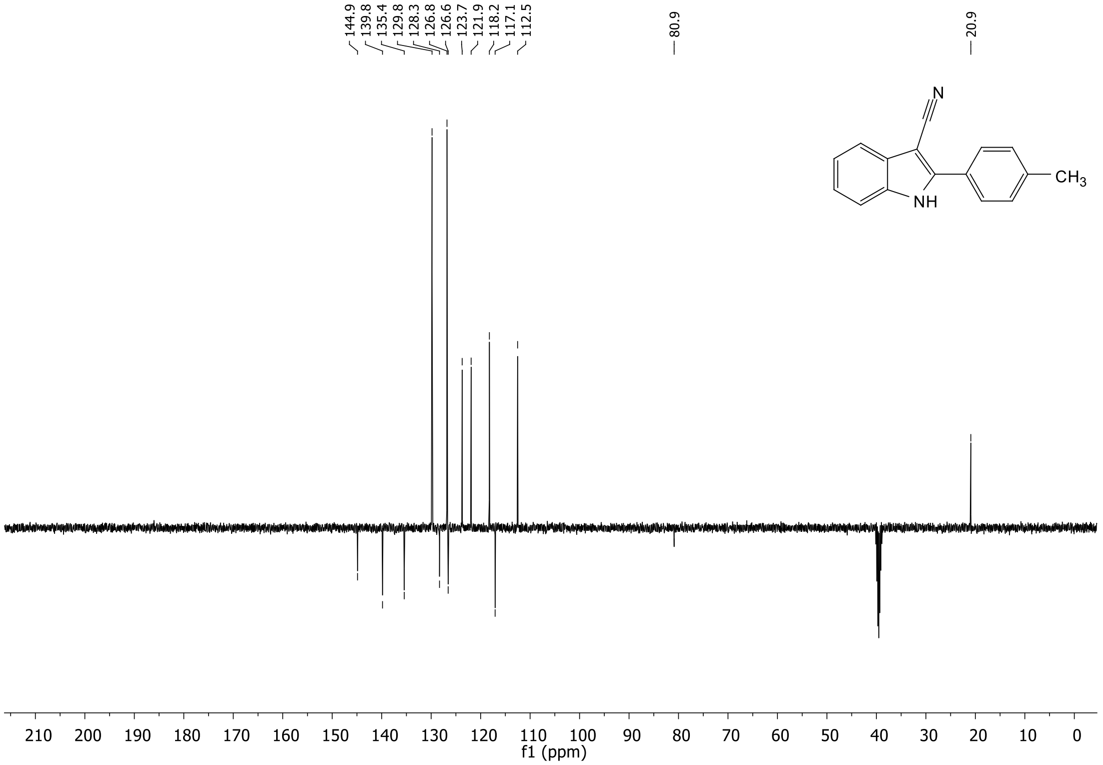

^{1}H NMR (400 MHz, DMSO-d_6) of 2l

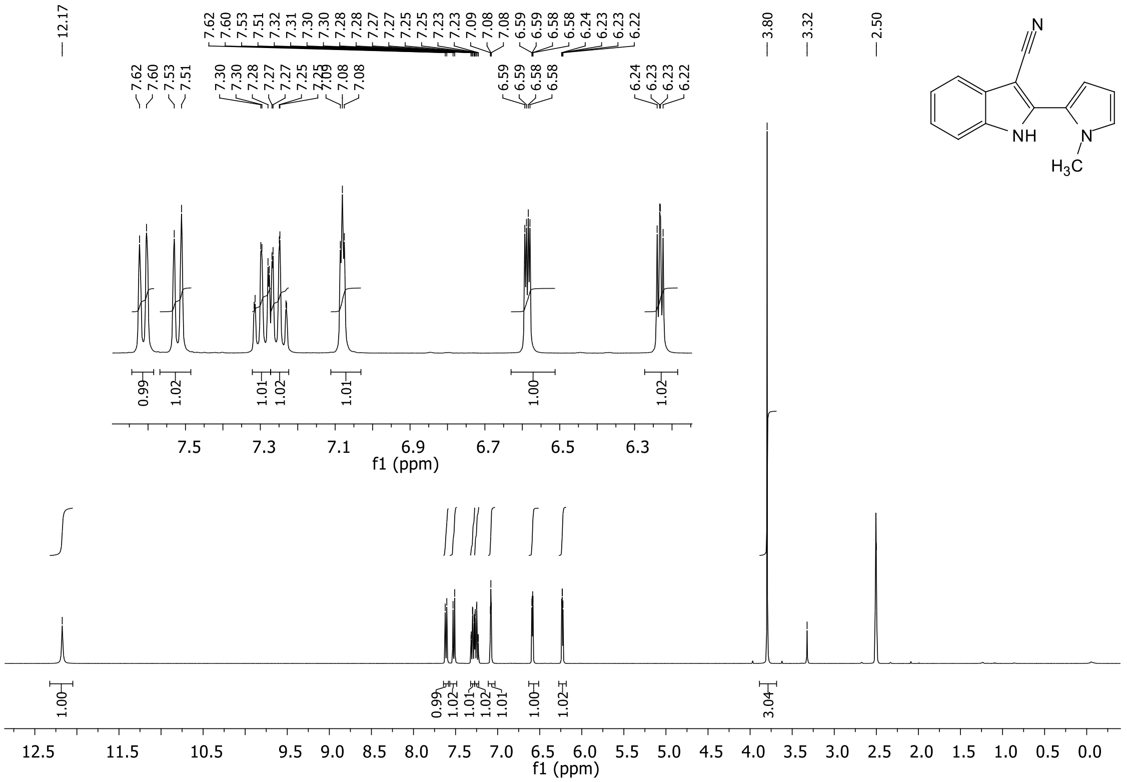

^{13}C {^{1}H} NMR (100 MHz, DMSO-d_6) of 2l

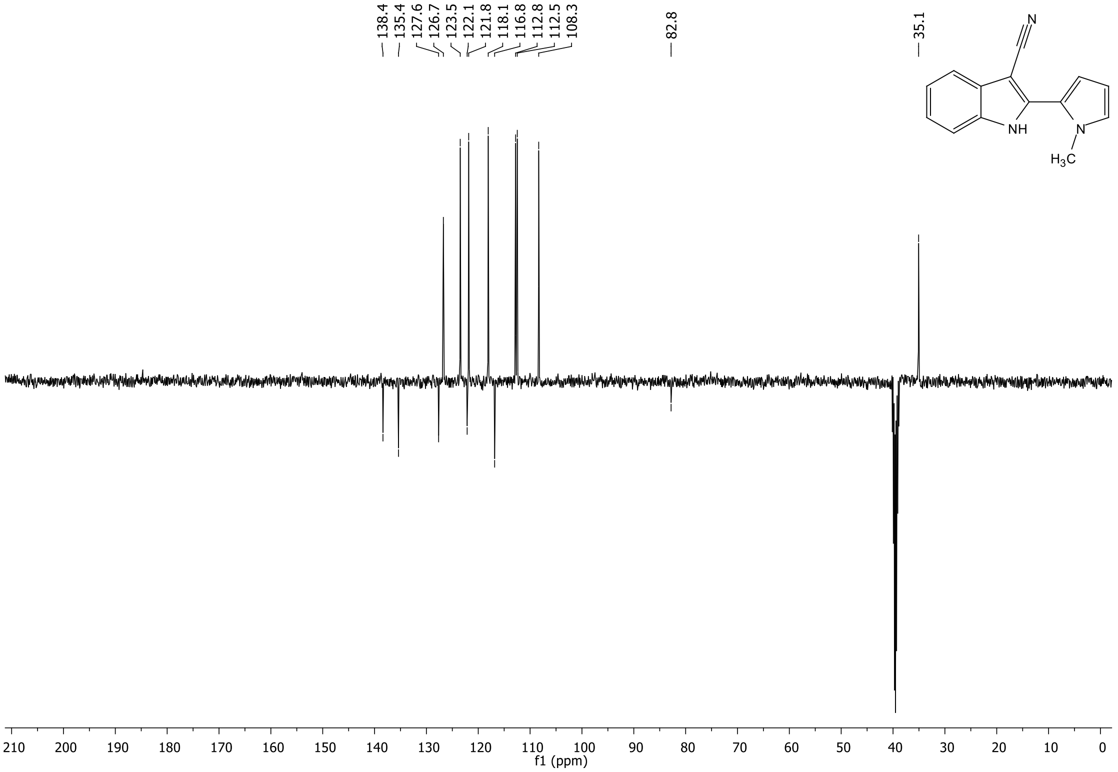

^{1}H NMR (400 MHz, DMSO-d_6) of 2m

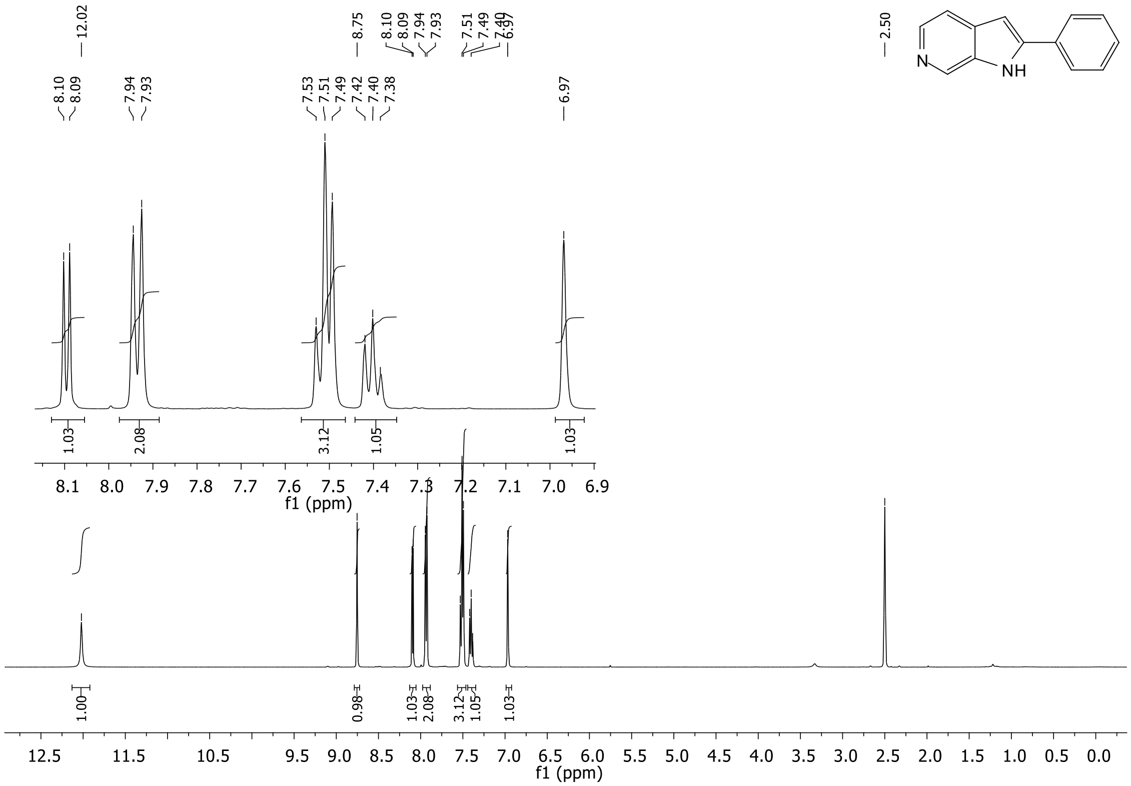

^{13}C {^{1} H} NMR (100 MHz, DMSO-d_6) of 2m

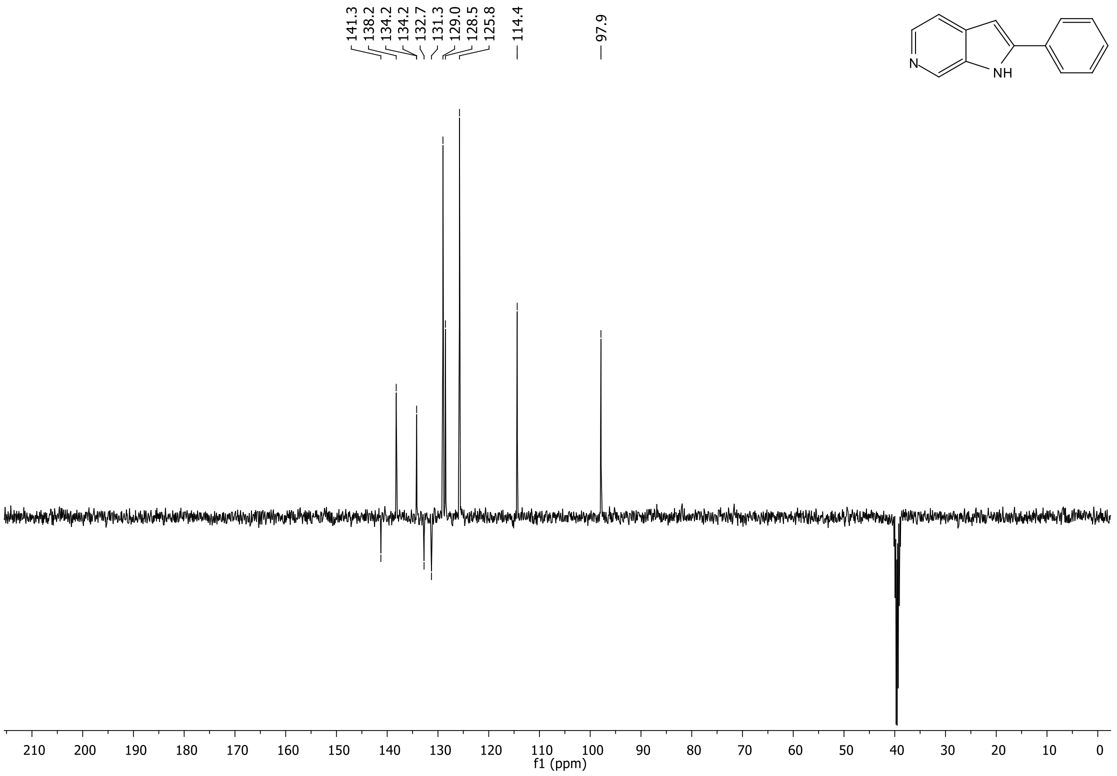

^{1}H NMR (400 MHz, DMSO-d_6) of 2n

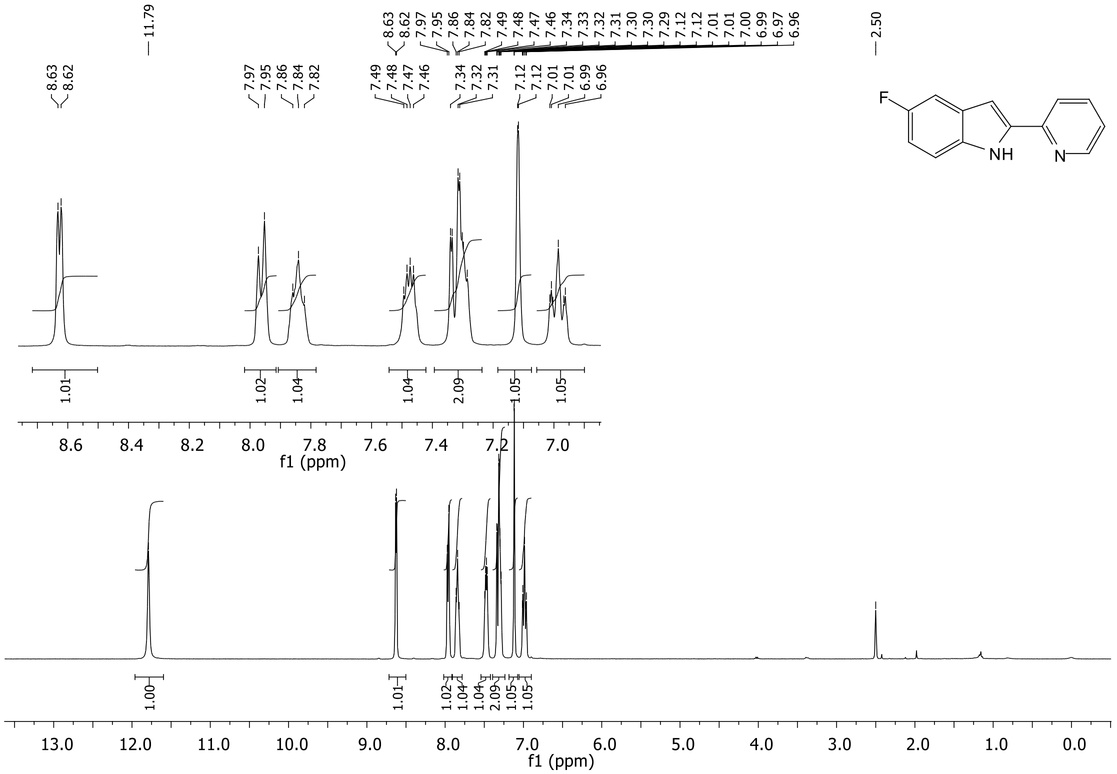

^{13}C {^{1} H} NMR (100 MHz, DMSO-d_6) of 2n

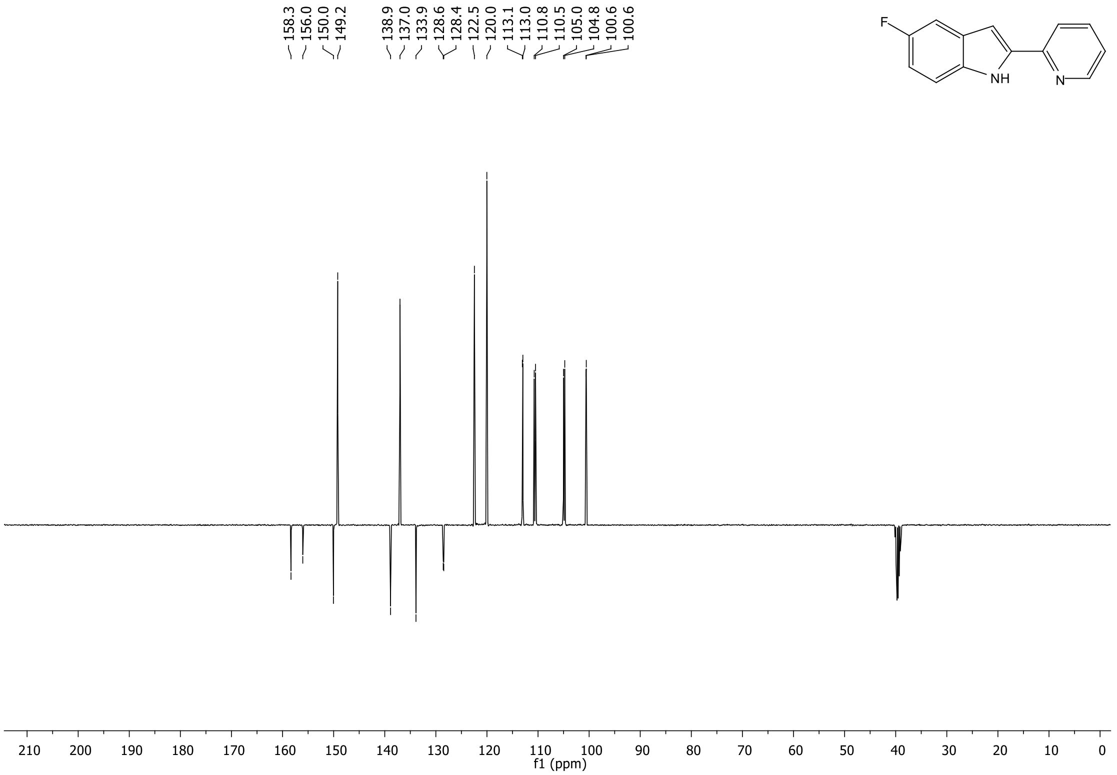

^{19}F NMR (376 MHz, DMSO-d_6) of 2n

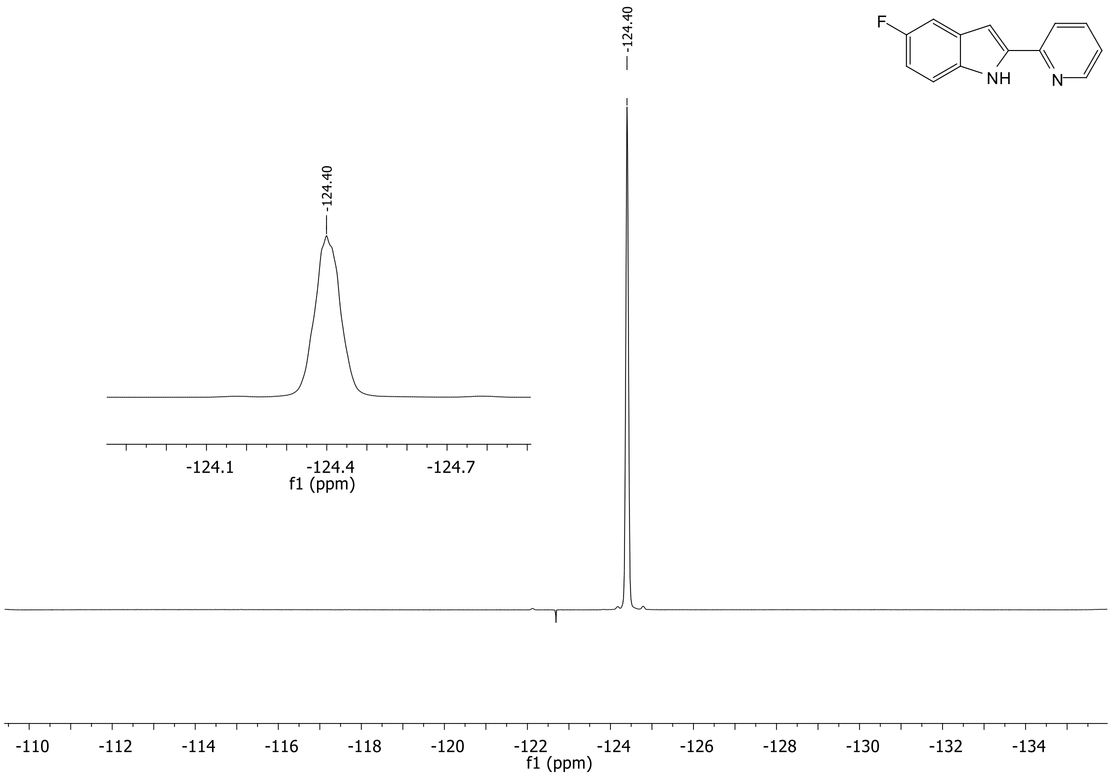

^{1}H NMR (400 MHz, DMSO-d_6) of 2o

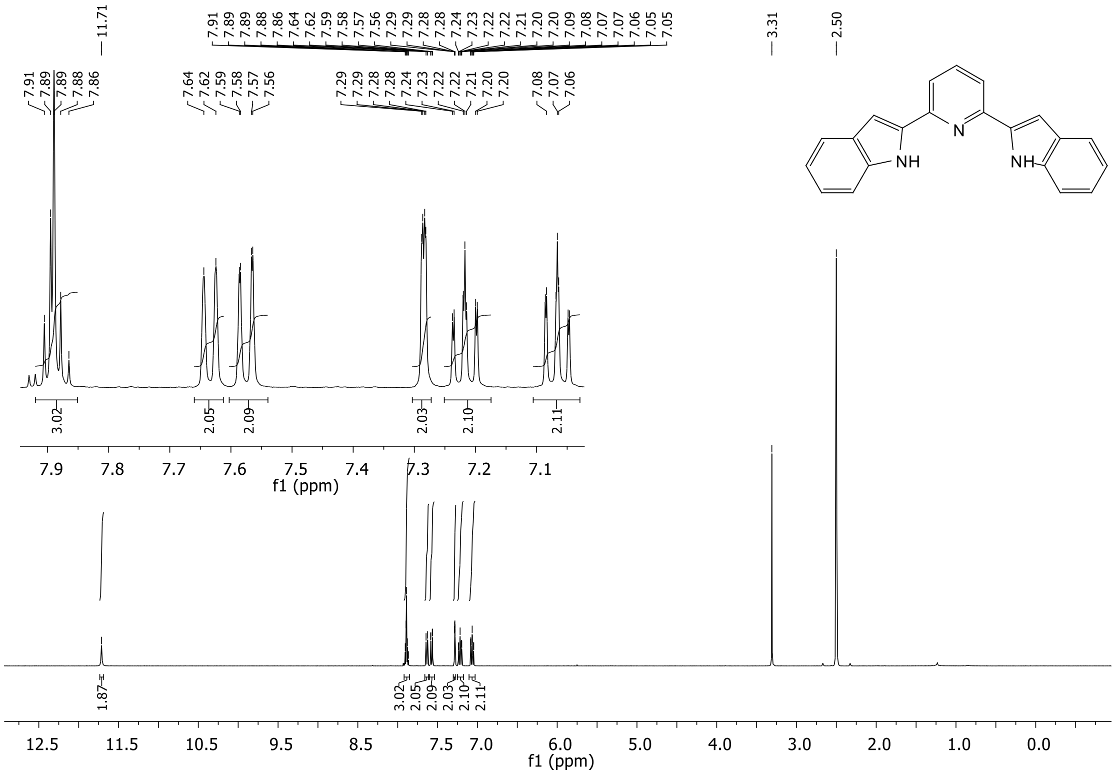

^{13}C {^{1} H} NMR (100 MHz, DMSO-d_6) of 2o

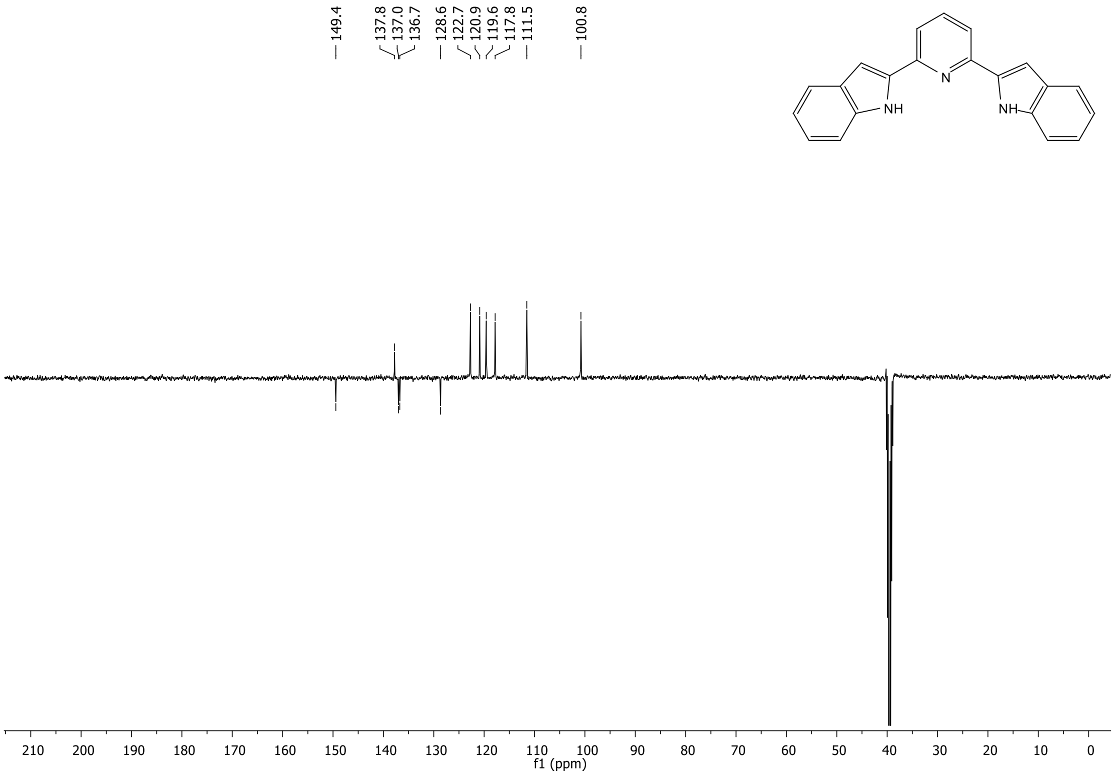

^{1}H NMR (400 MHz, $CDCl_3$) of 2p

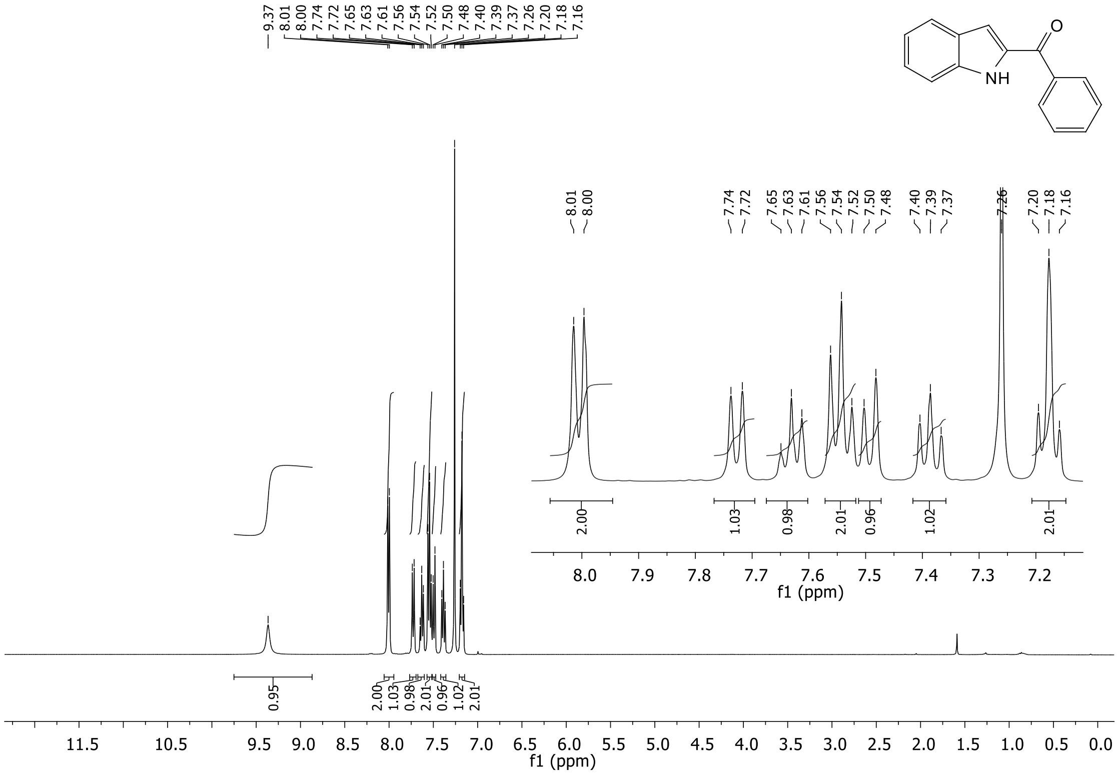

^{13}C {^{1} H} NMR (100 MHz, $CDCl_3$) of 2p

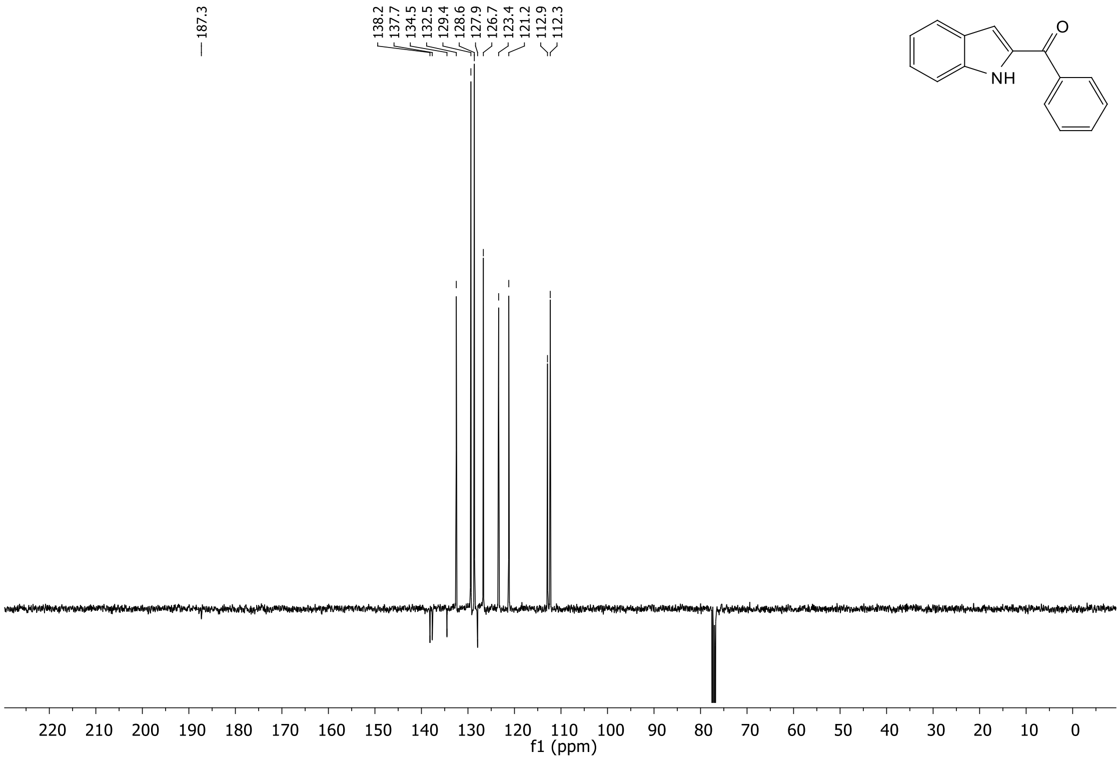

^{1}H NMR (400 MHz, $CDCl_3$) of 2q

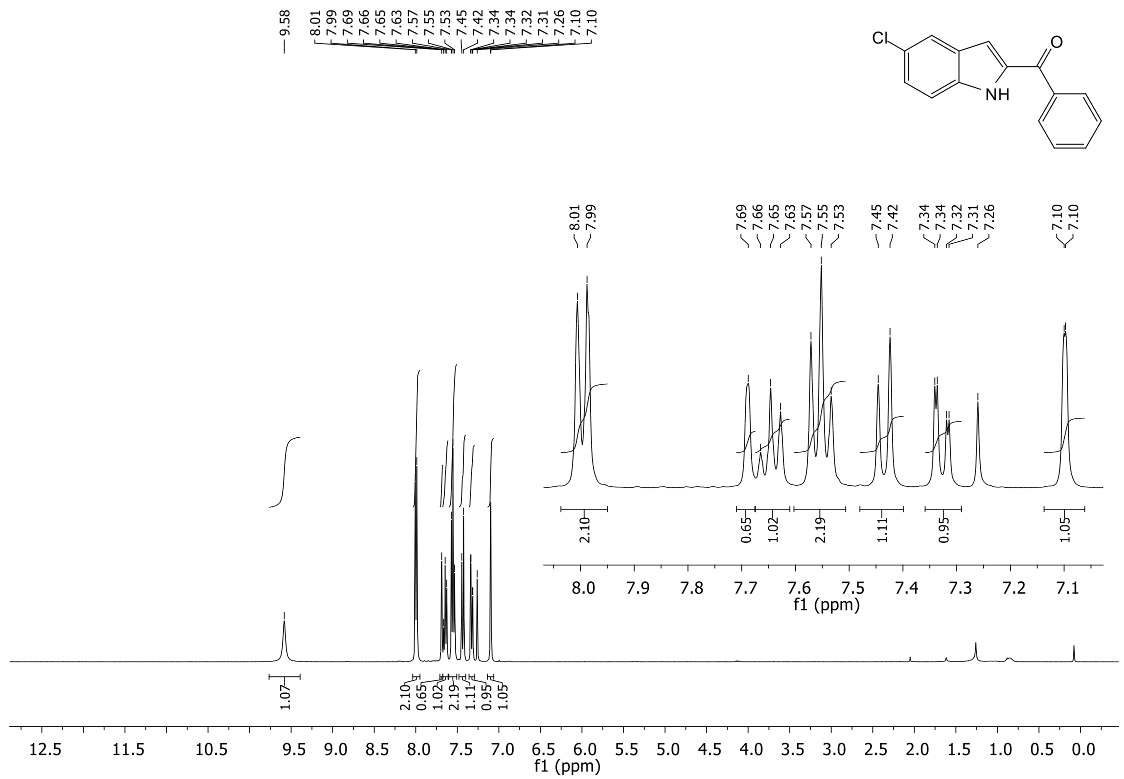

^{13}C {[1] H} NMR (100 MHz, $CDCl_3$) of 2q

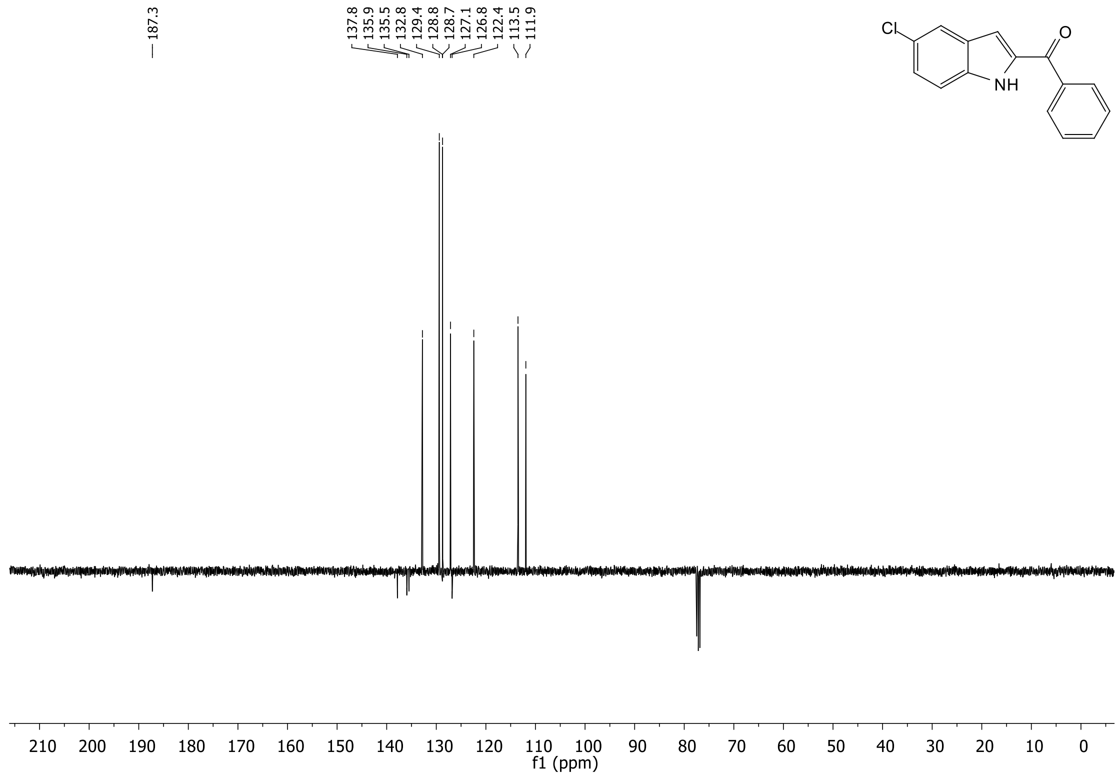

^{1}H NMR (400 MHz, DMSO-d_6) of 2r

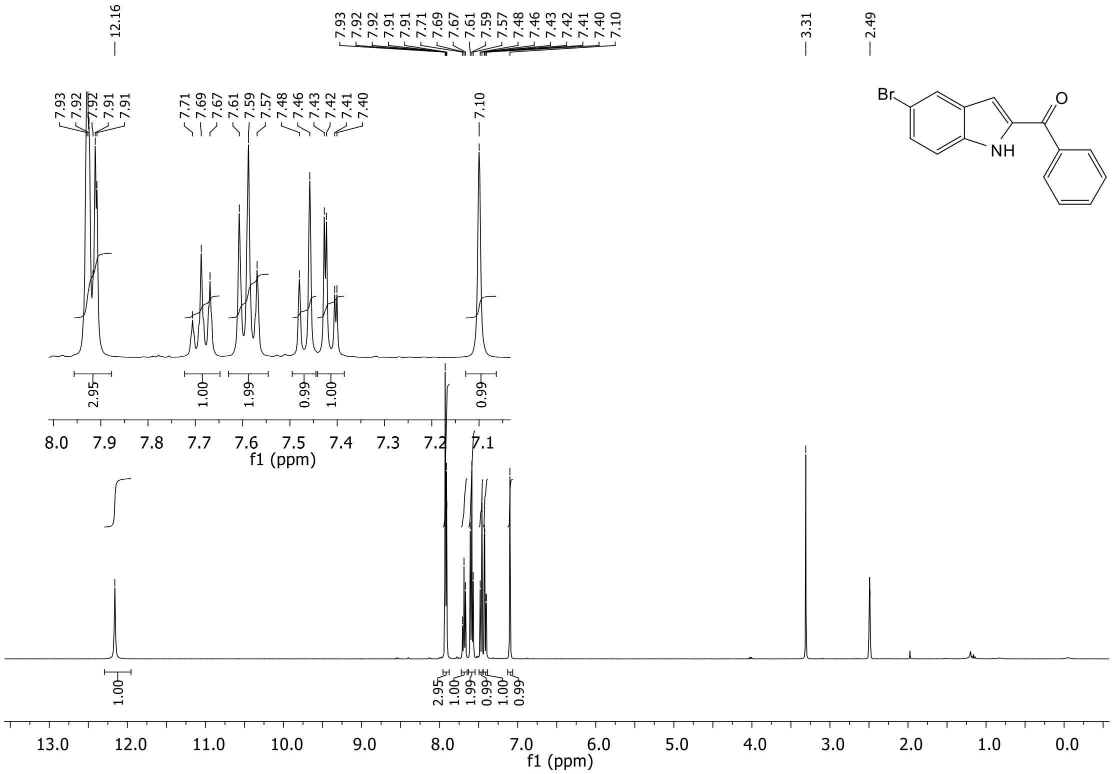

$^{13}C\ \{^{1}H\}$ NMR (100 MHz, DMSO-d_6) of 2r

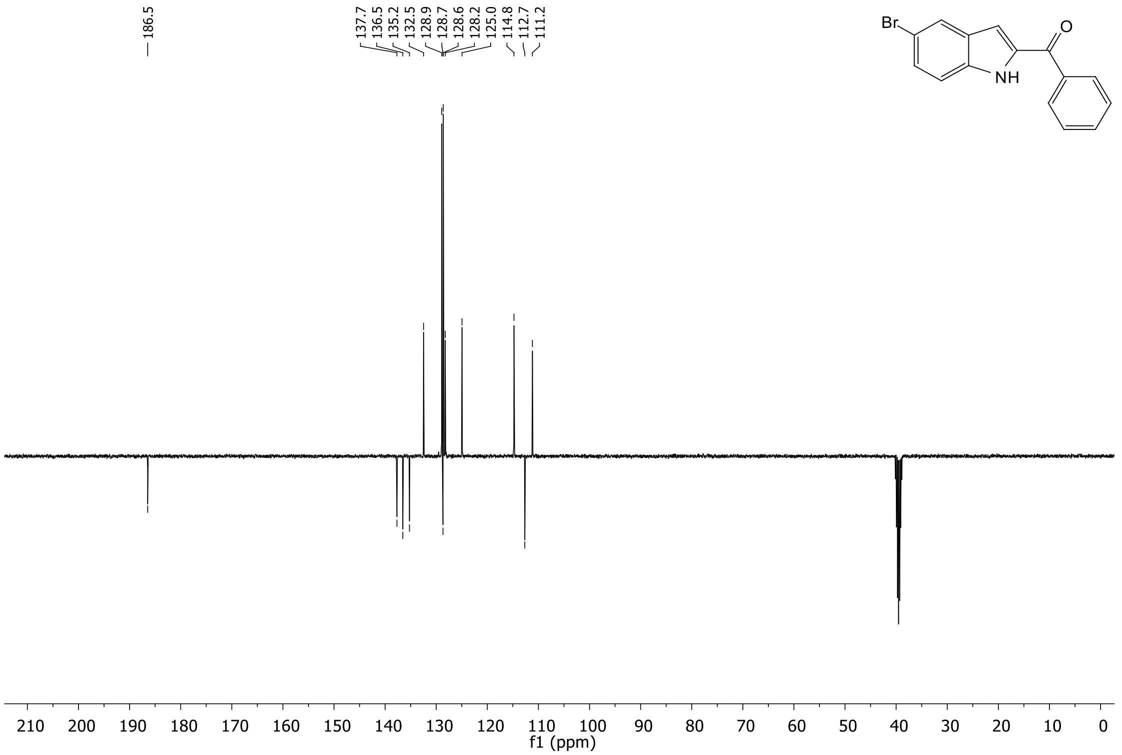

^{1}H NMR (400 MHz, $CDCl_3$) of 2s

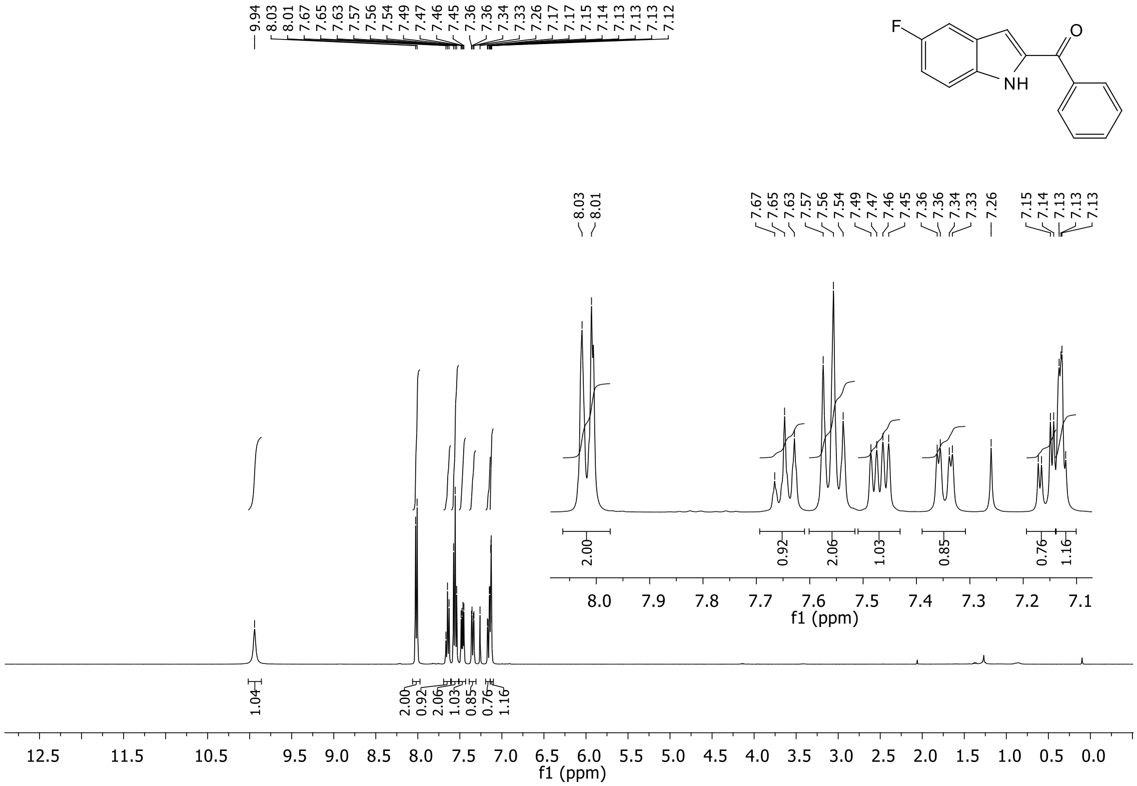

^{13}C {^{1}H} NMR (100 MHz, $CDCl_3$) of 2s

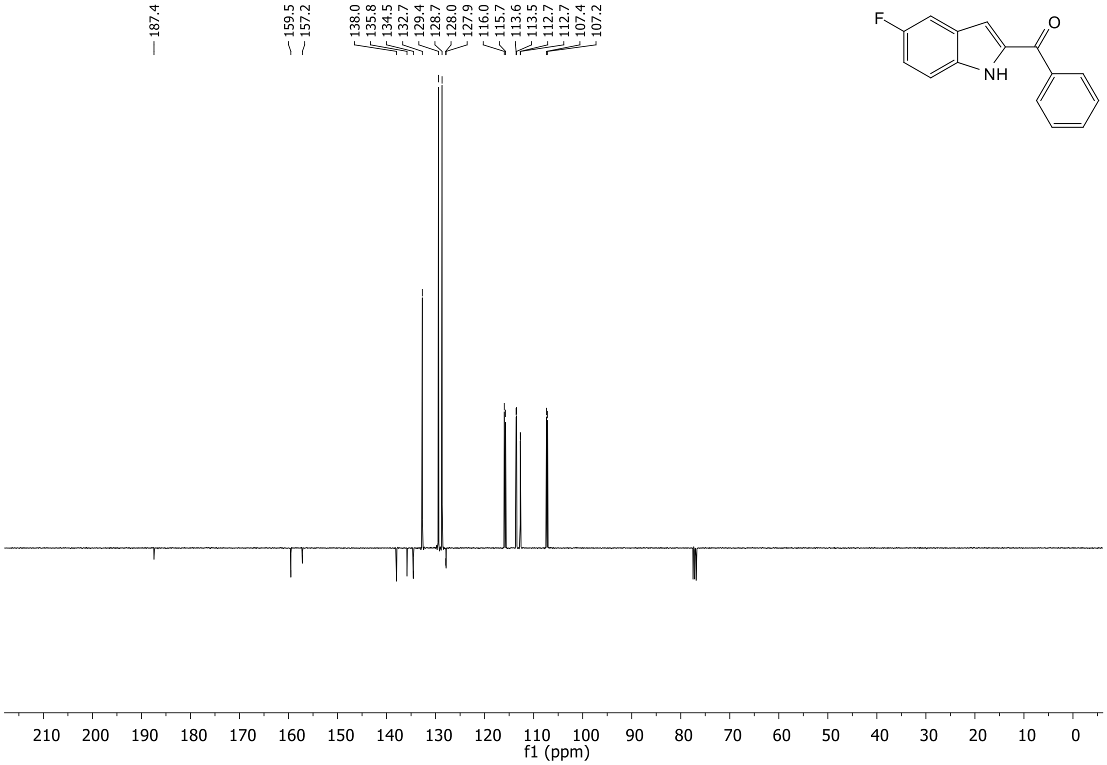

^{19}F NMR (376 MHz, $CDCl_3$) of 2s

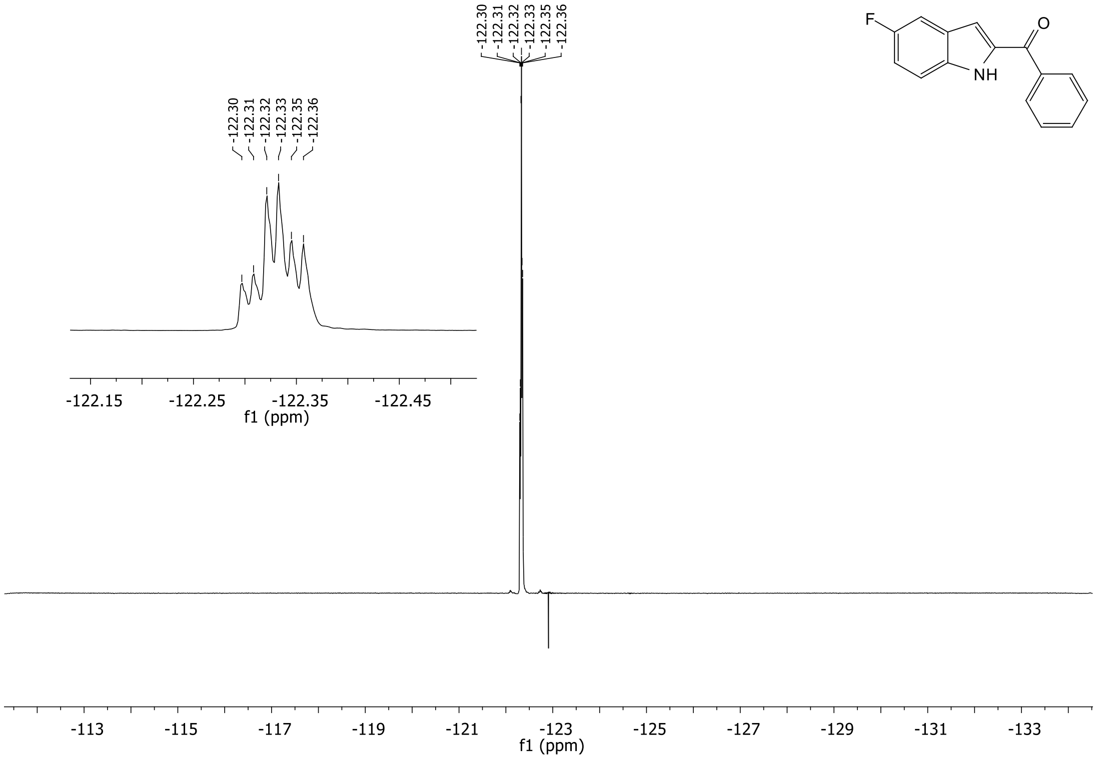

^{1}H NMR (400 MHz, $CDCl_3$) of 2t

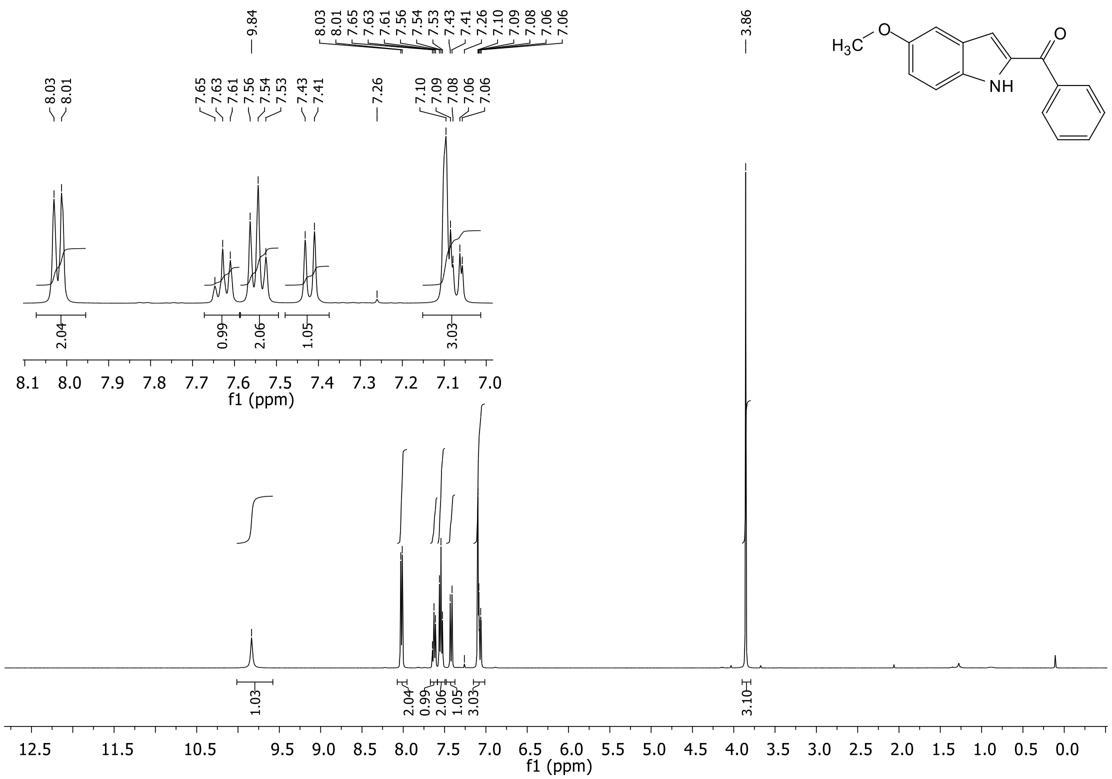

^{13}C {^{1} H} NMR (100 MHz, $CDCl_3$) of 2t

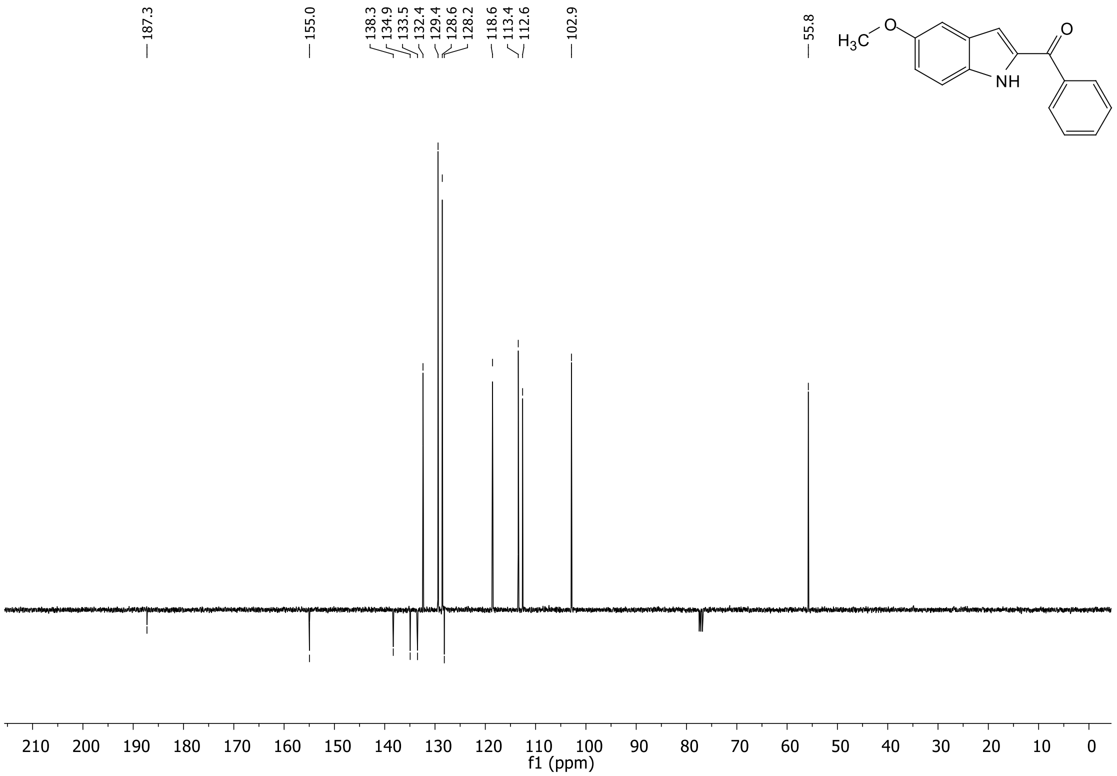

^{1}H NMR (400 MHz, DMSO-d_6) of 2u

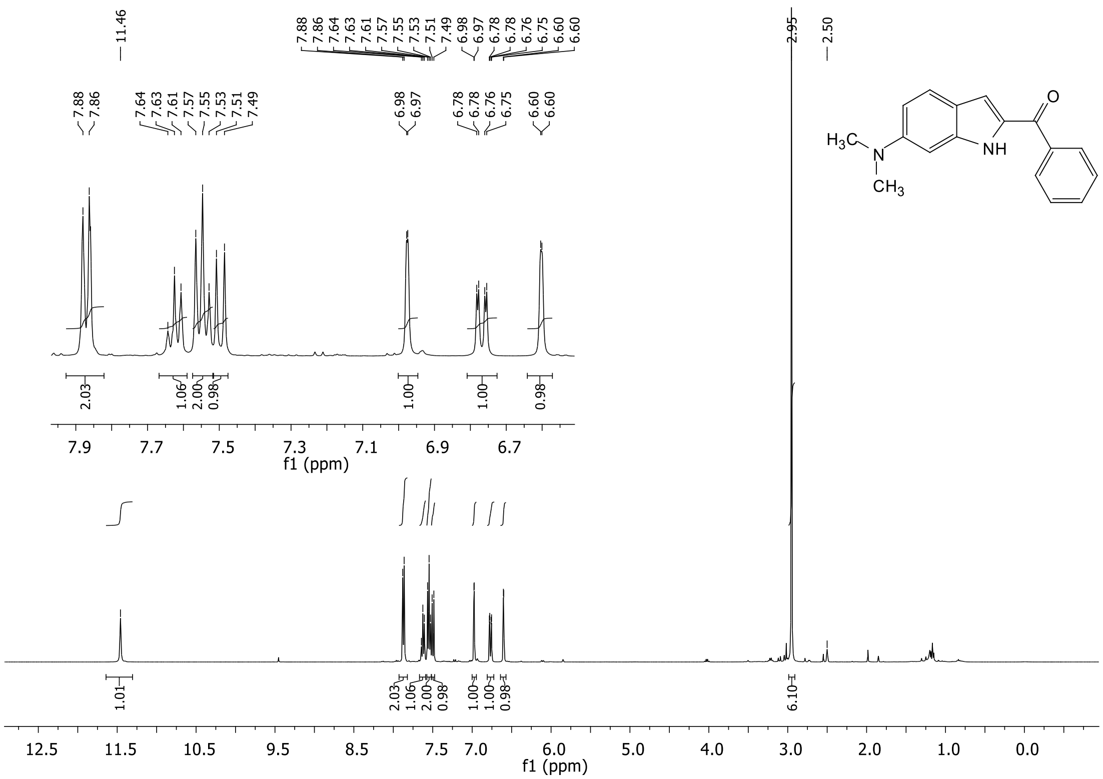

^{13}C {^{1} H} NMR (100 MHz, DMSO-d_6) of 2u

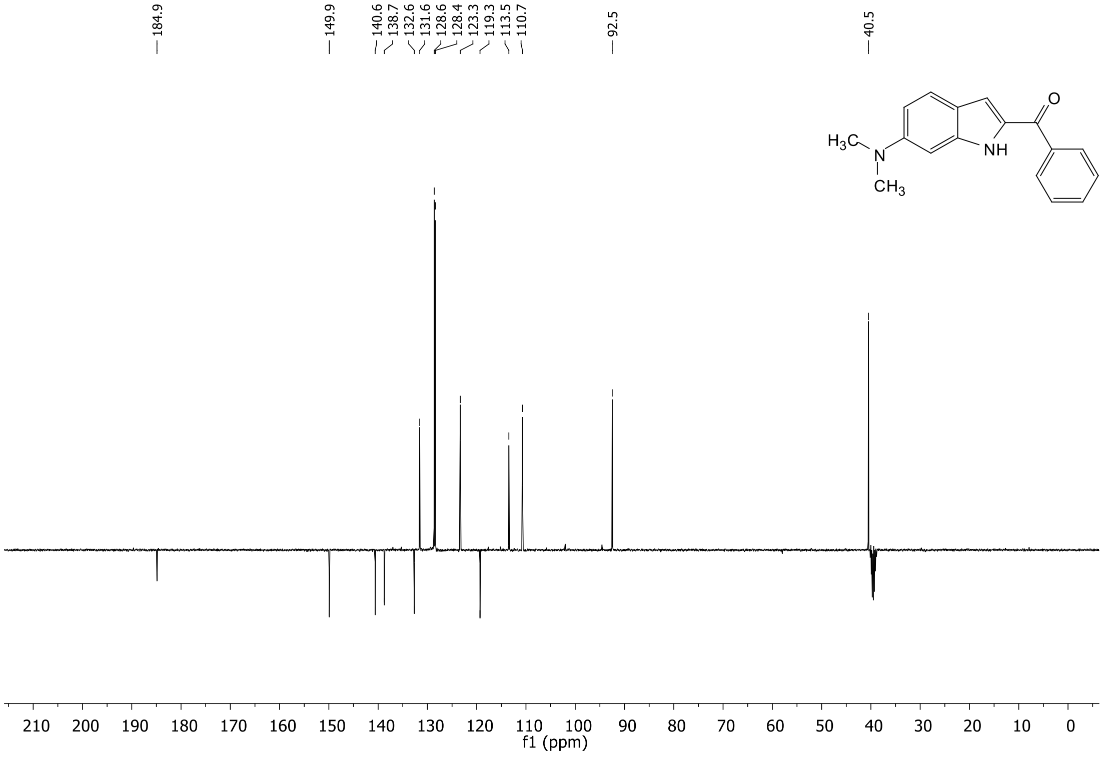

^{1}H NMR (400 MHz, DMSO-d_6) of 2v

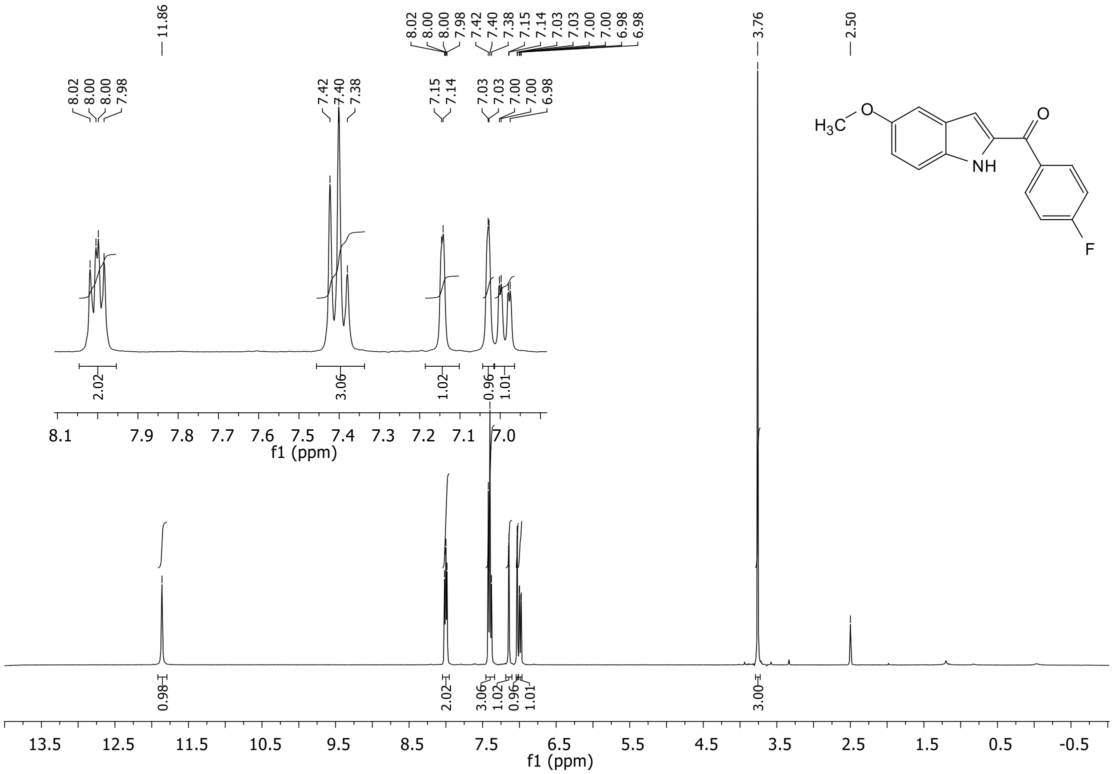

^{13}C {^{1} H} NMR (100 MHz, DMSO-d_6) of 2v

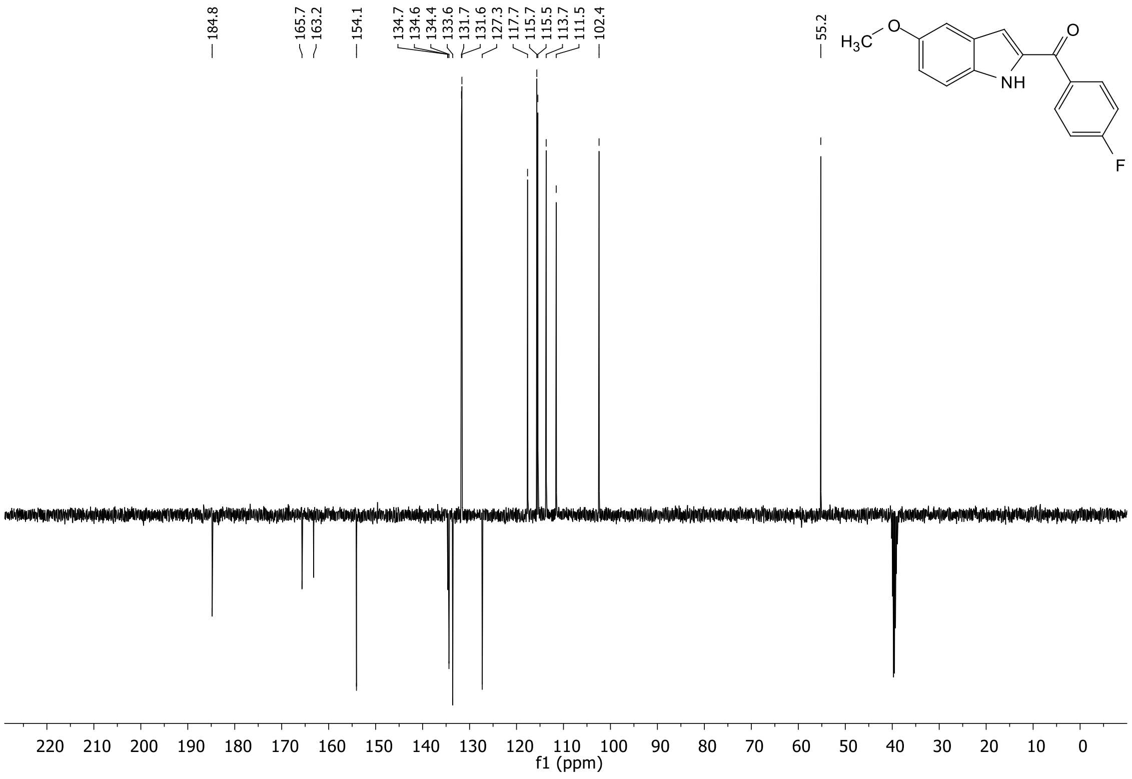

^{19}F NMR (376 MHz, DMSO-d_6) of 2v

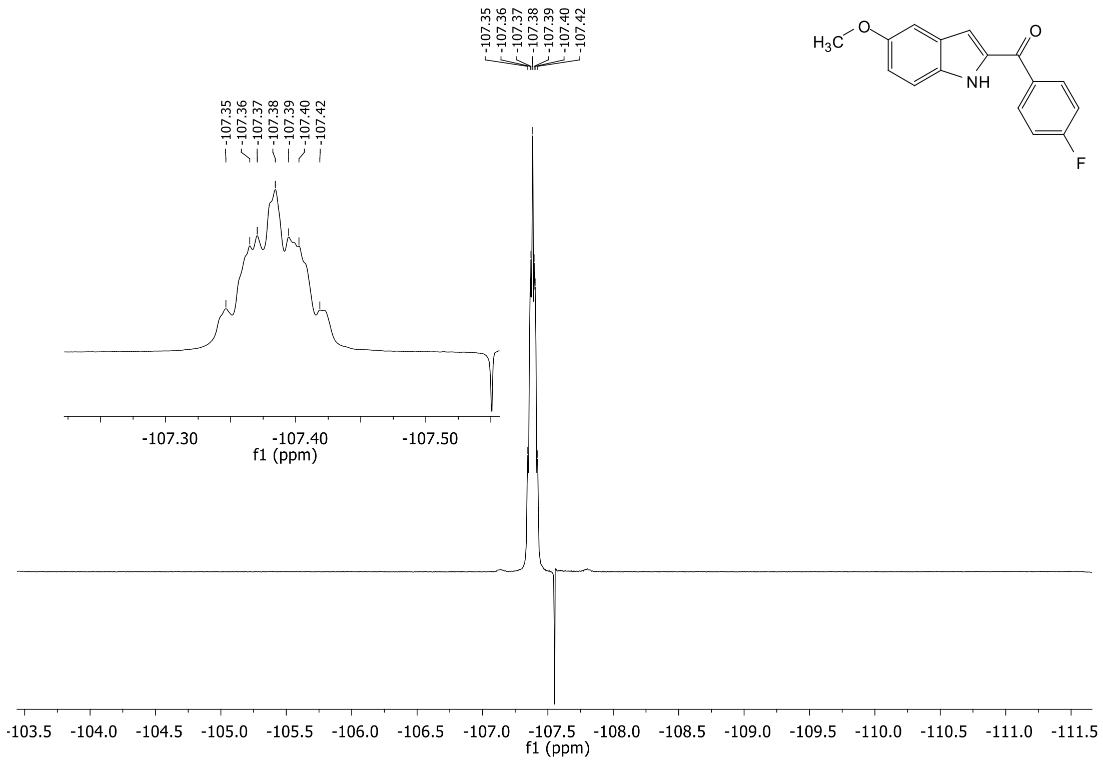

^{1}H NMR (400 MHz, DMSO-d_6) of 2w

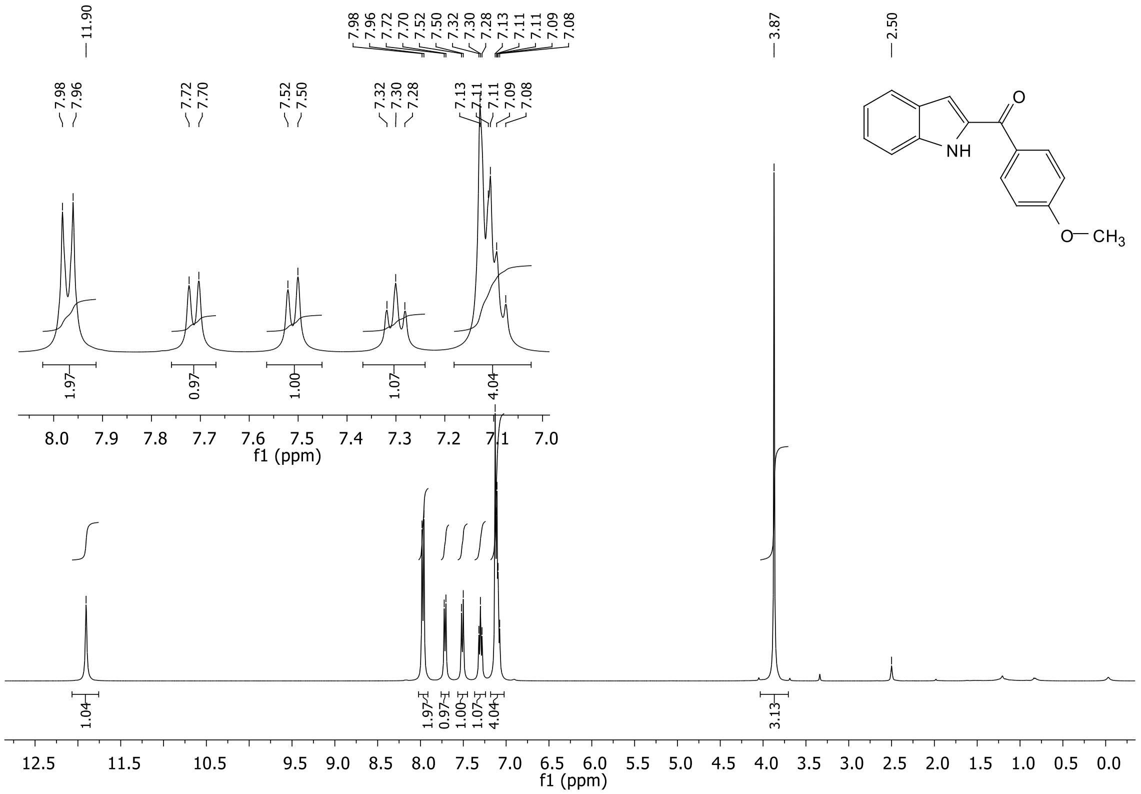

^{13}C {^{1}H} NMR (100 MHz, DMSO-d_6) of 2w

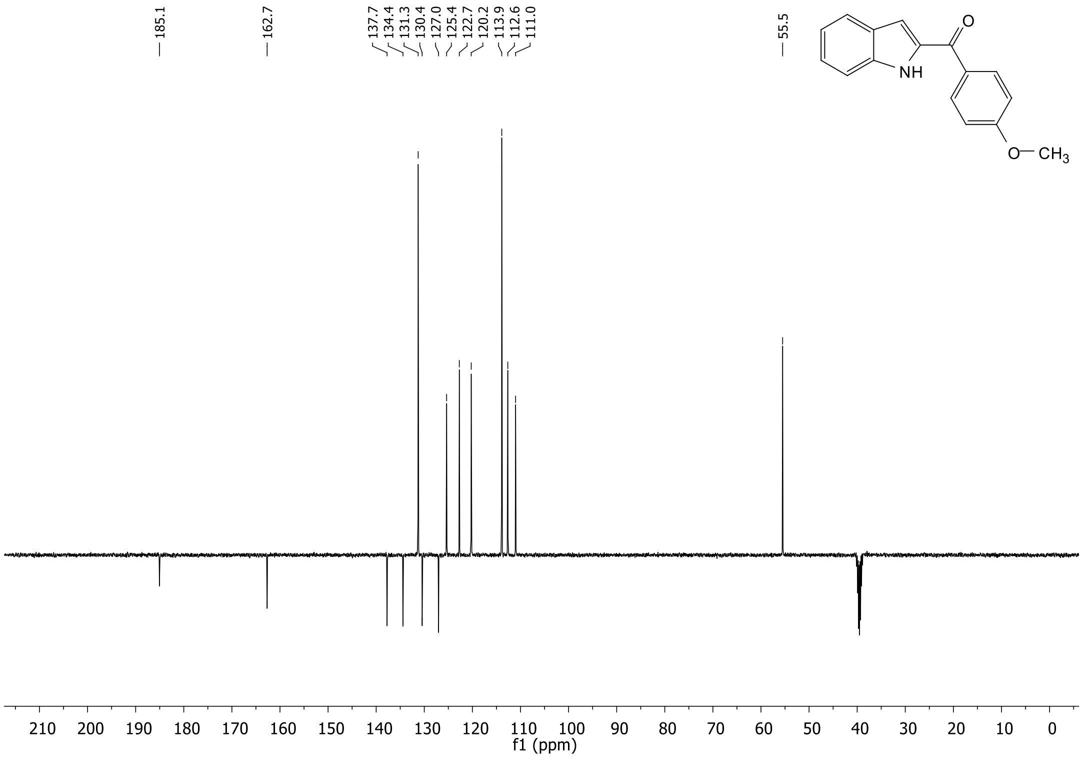

^{1}H NMR (400 MHz, DMSO-d_6) of 2x

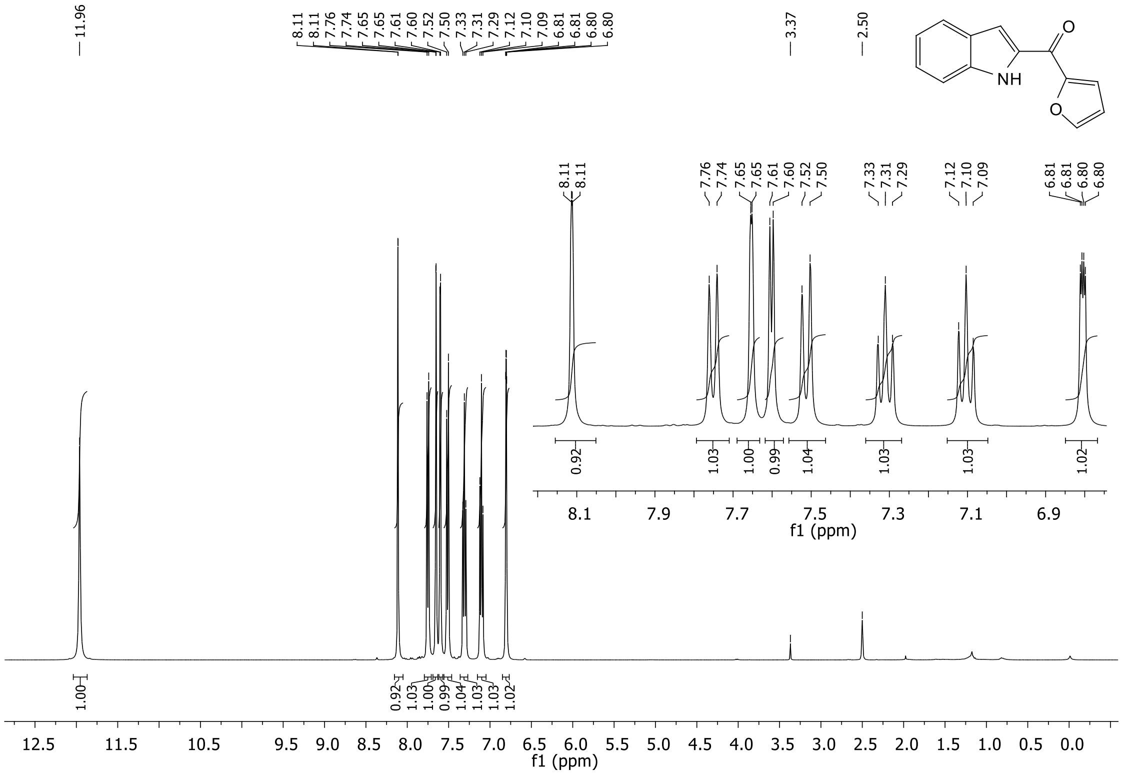

^{13}C {^{1} H} NMR (100 MHz, DMSO-d_6) of 2x

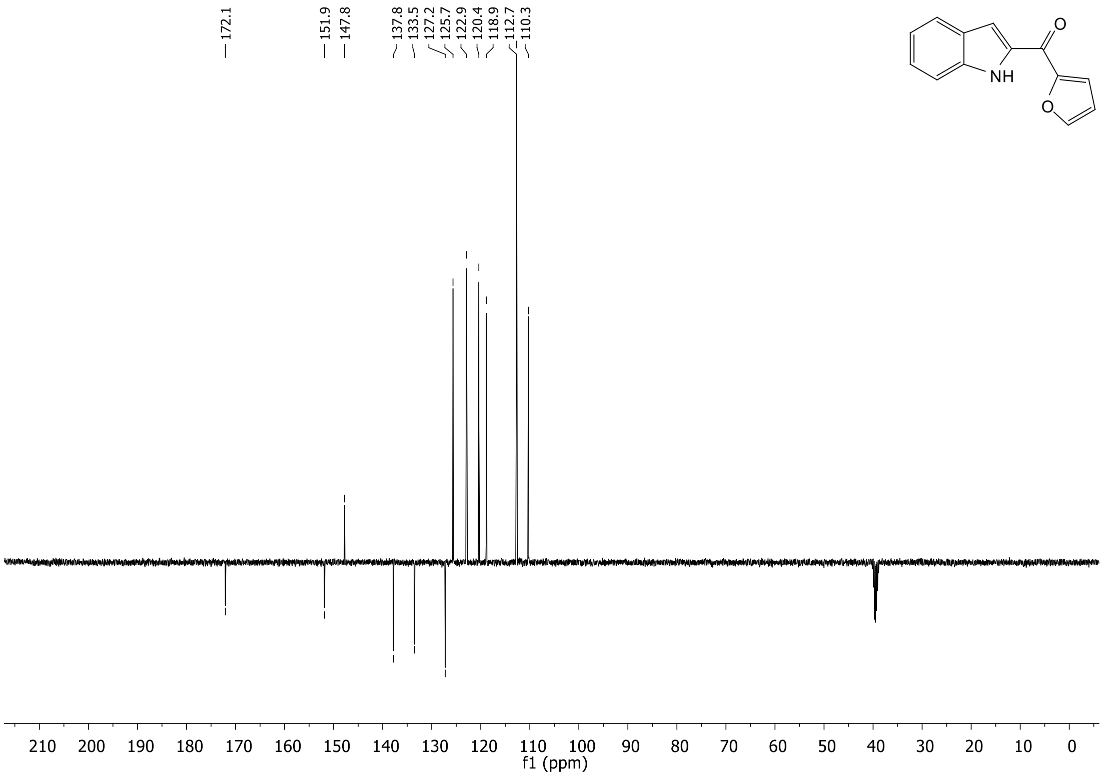

^{1}H NMR (400 MHz, $CDCl_3$) of 2y

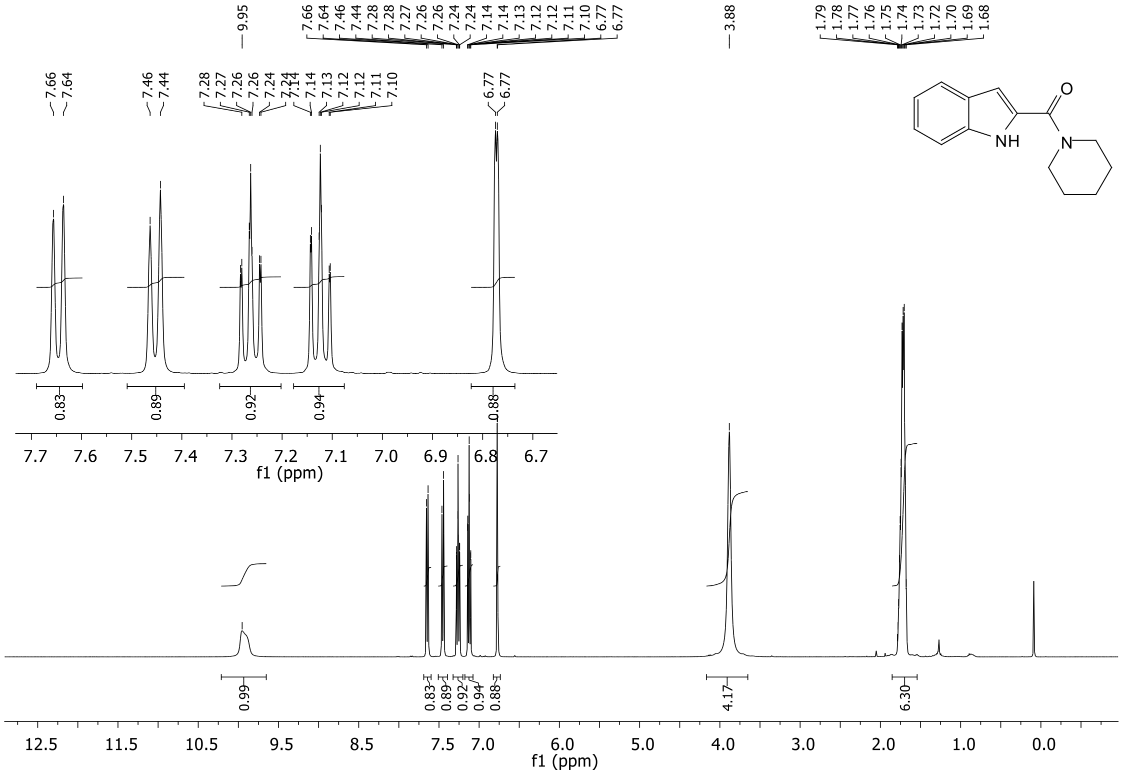

^{13}C {^{1}H} NMR (100 MHz, $CDCl_3$) of 2y

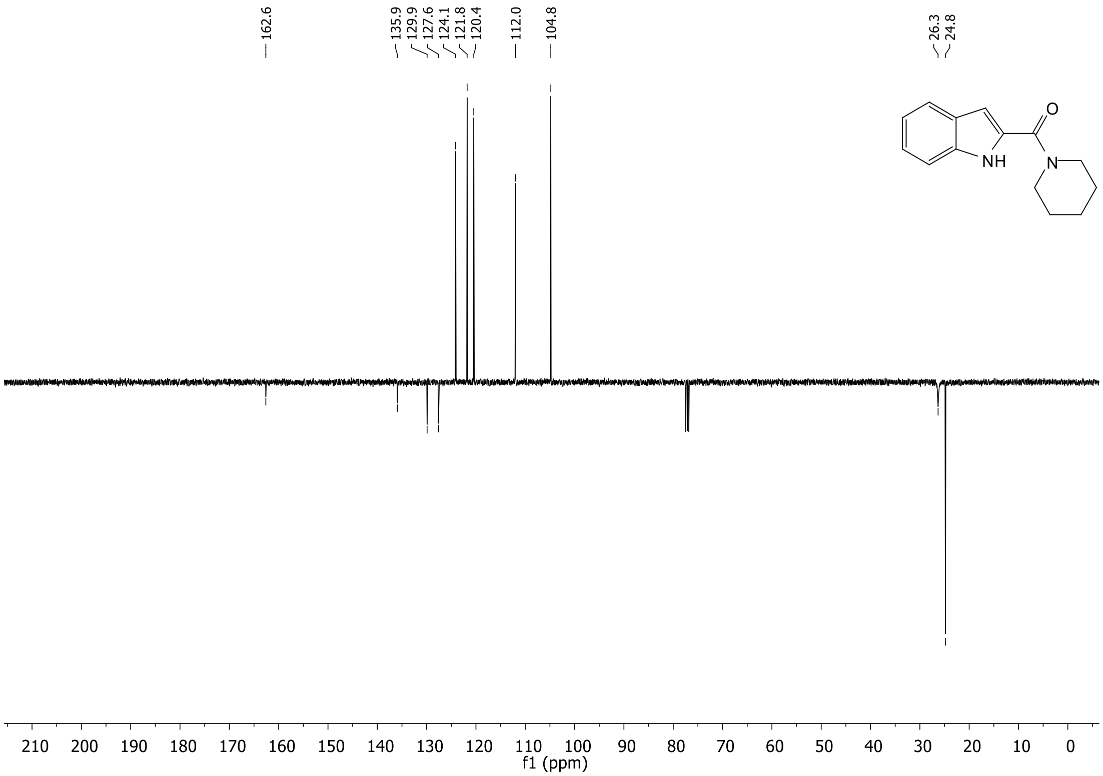

^{1}H NMR (400 MHz, DMSO-d_6) of 2z

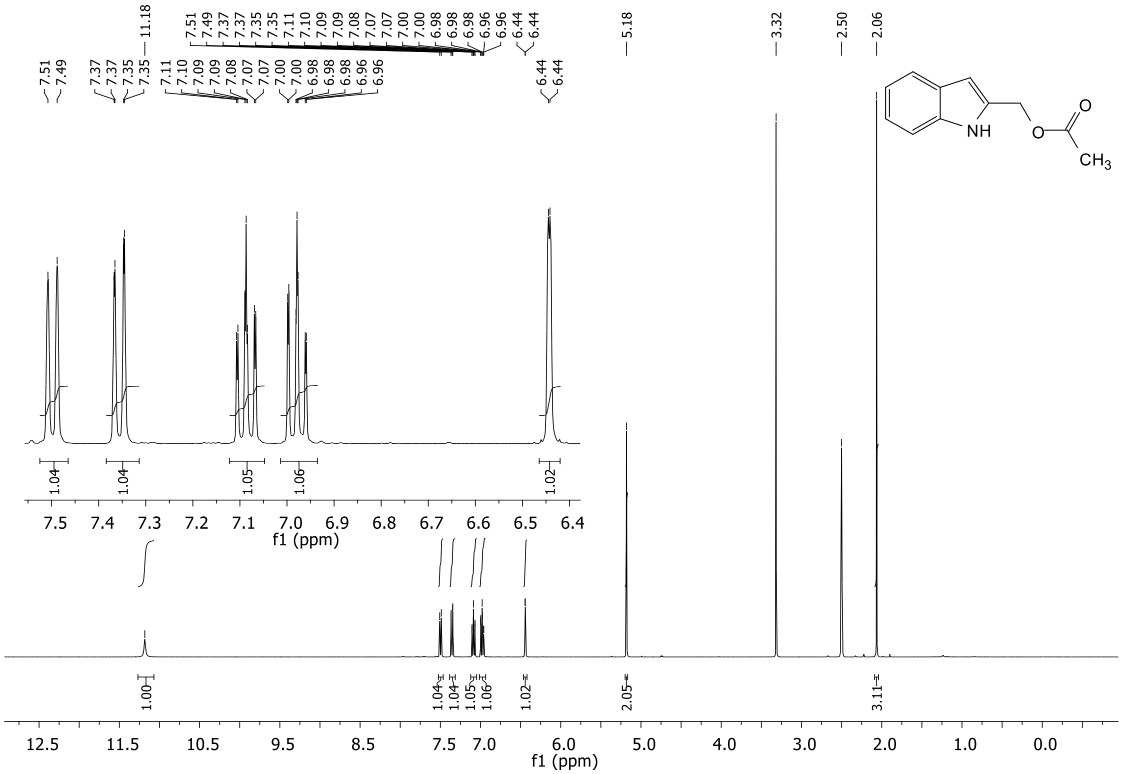

^{13}C {[1] H} NMR (100 MHz, DMSO-d_6) of 2z

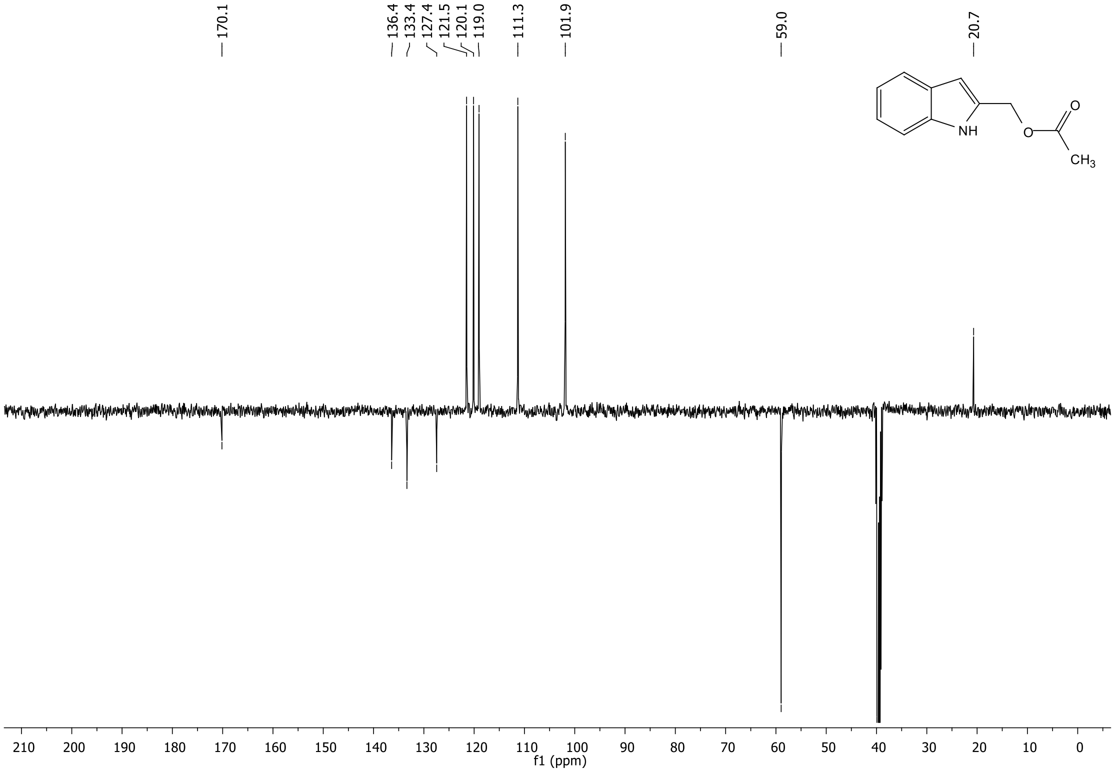

^{1}H NMR (400 MHz, DMSO-d_6) of 2aa

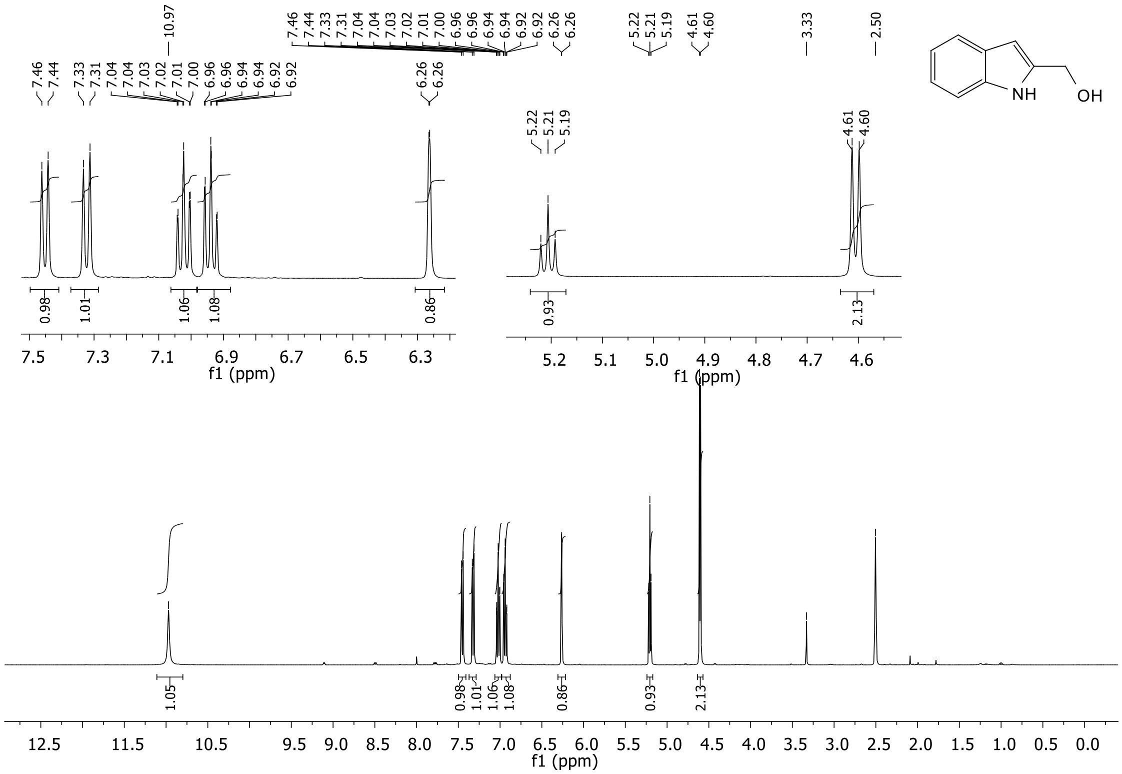

^{13}C {^{1}H} NMR (100 MHz, DMSO-d_6) of 2aa

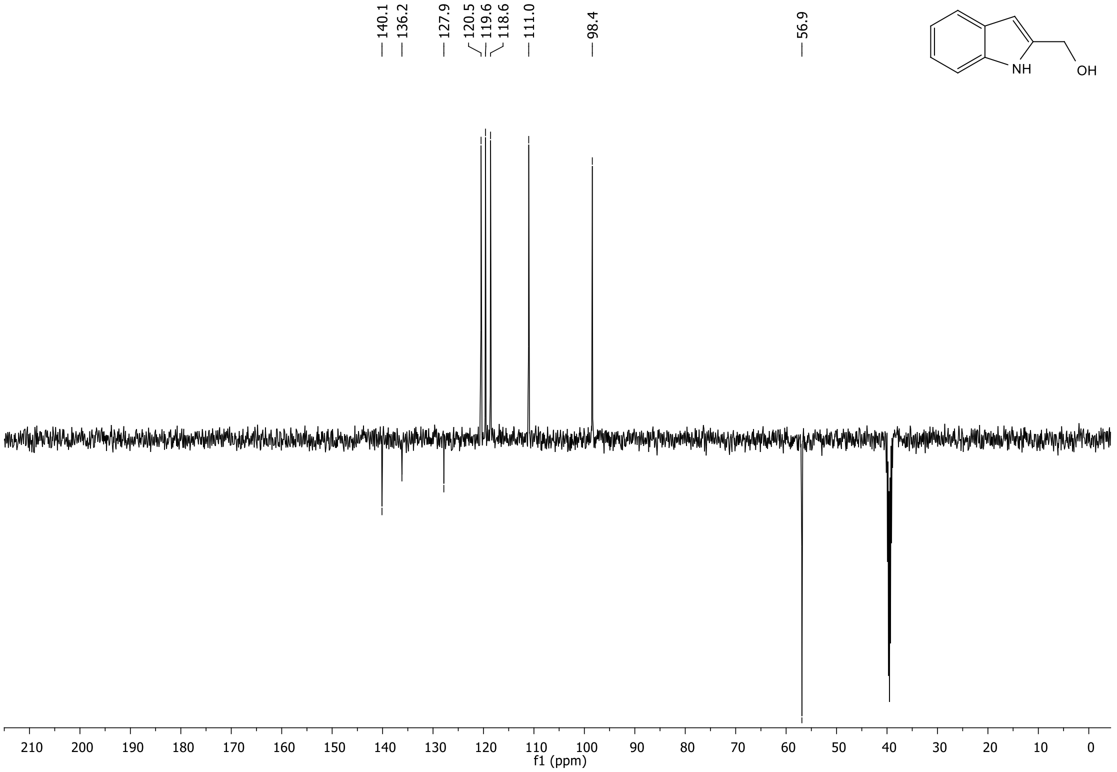

^{1}H NMR (400 MHz, $CDCl_3$) of 2ab

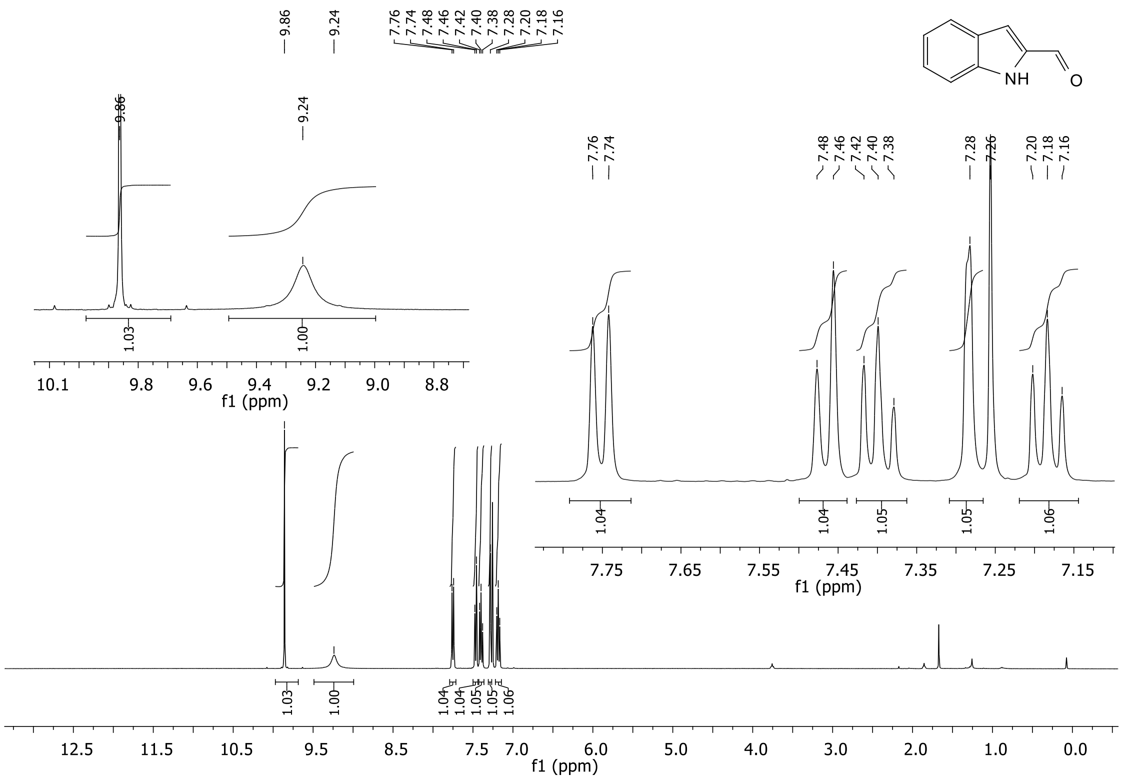

^{13}C {^{1} H} NMR (100 MHz, $CDCl_3$) of 2ab

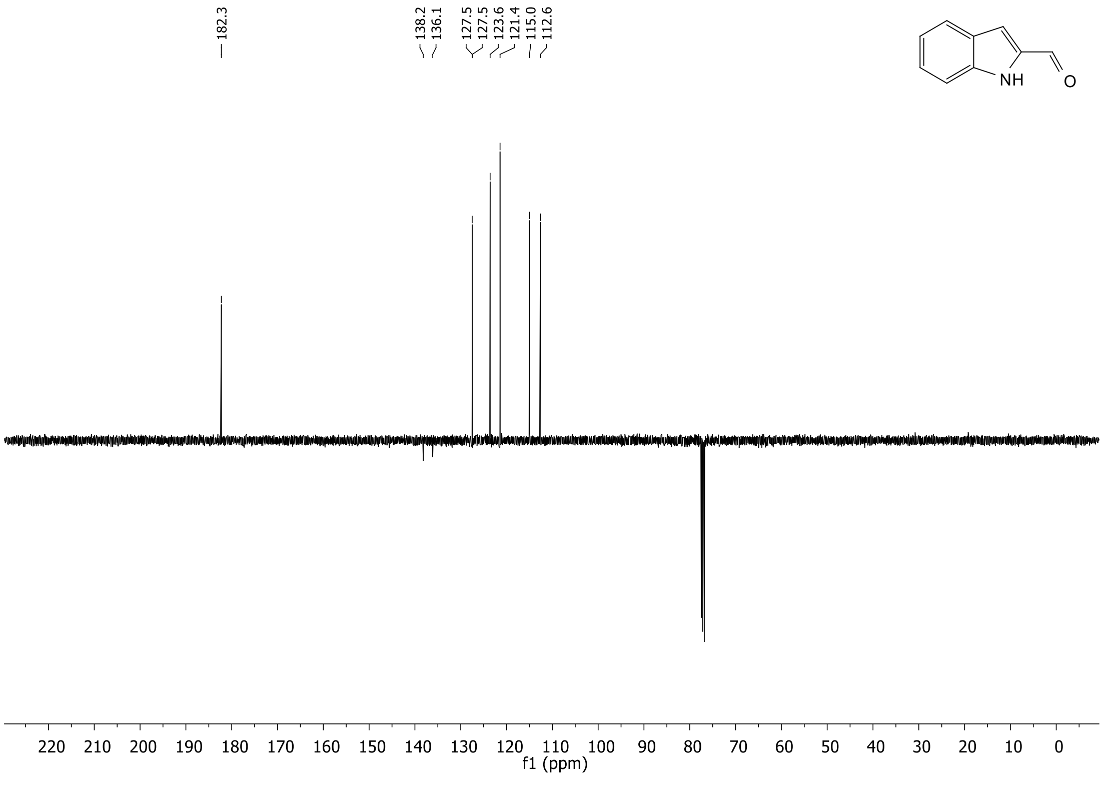

Supplement: Supplementary file 1 — jo2c02613_si_001.pdf [file jo2c02613_si_001.pdf]
