# Supplementary figures and images for: Formic Acid as Carbon Monoxide Source in the Palladium-Catalyzed N-Heterocyclization of o-Nitrostyrenes to Indoles
Source: J Org Chem. 2023 Jan 19;88(8):5108–17. doi: 10.1021/acs.joc.2c02613 (PMC10127278; doi:10.1021/acs.joc.2c02613)

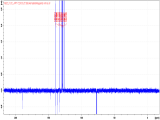

Supplement: Supplementary file 2 — jo2c02613_si_002.zip [file jo2c02613_si_002.zip › FID for Publication/1q/1q - 13C/pdata/1/thumb.png]

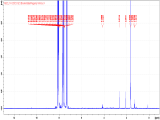

Supplement: Supplementary file 2 — jo2c02613_si_002.zip [file jo2c02613_si_002.zip › FID for Publication/1q/1q - 1H/pdata/1/thumb.png]

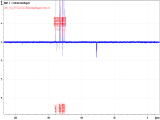

Supplement: Supplementary file 2 — jo2c02613_si_002.zip [file jo2c02613_si_002.zip › FID for Publication/1r/1r - 13C/pdata/1/thumb.png]

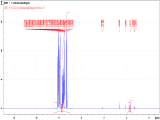

Supplement: Supplementary file 2 — jo2c02613_si_002.zip [file jo2c02613_si_002.zip › FID for Publication/1r/1r - 1H/pdata/1/thumb.png]

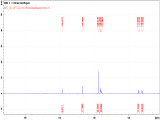

Supplement: Supplementary file 2 — jo2c02613_si_002.zip [file jo2c02613_si_002.zip › FID for Publication/1s/1s - 13C/pdata/1/thumb.png]
